# Neural crest cell signatures drive tumorigenesis in tuberous sclerosis complex and lymphangioleiomyomatosis

**DOI:** 10.1172/jci.insight.195013

**Published:** 2026-06-02

**Authors:** Uchenna J. Unachukwu, Enio B. Garcia, Nooralam Rai, Jeanine M. D’Armiento

**Affiliations:** 1 Center for LAM and Rare Lung Disease, Department of Anesthesiology, College of Physicians and Surgeons, Columbia University Medical Center, New York, New York, USA.; 2 Department of Pediatrics (Pulmonary), Columbia University Medical Center, New York, New York, USA.

**Keywords:** Cell biology, Clinical Research, Oncology, Biomarkers, Cancer, Stem Cells

## Abstract

Tuberous sclerosis complex (TSC) and lymphangioleiomyomatosis (LAM) lack well-defined cellular origins, limiting treatment options. In this report, scRNA-seq of *Tsc2^+/–^* mouse renal cystadenomas revealed an 80-fold increase in a tumor cell subpopulation with neural crest features, expressing known cranial neural crest genes as SRY box transcription factor 9 (*Sox9*), transcription factor activator protein (*Tfap2a*), and candidate neurocristopathy markers, osteopontin (*Spp1*), lipocalin-2 (*Lcn2*), clusterin (*Clu*), and cytokeratin 18 (*Krt18*). These signatures were validated in mouse tumors and LAM patient lesions and serum, identifying a tumor phenotype distinct from traditional VEGFD detection. Pathway analysis indicated activation of WNT/SHH signaling, nephric duct formation, and protumorigenic signals, with transcription factor 7 (*Tcf7*) and ephrin-A ligands as key upstream regulators. *Spp1* KO in cranial neural crest cells (CNCCs) significantly reduced proliferation (28%–33%), migration (54%–76%), and invasion (29%–64%) without affecting viability, while *Tsc2* KO increased viability 3- to 6-fold with minimal effect on chemotaxis. Elevated serum levels of SPP1 and KRT18 in 1 subset of patients with LAM, decreased LCN2 in nearly all cases, and distinct increases in VEGFD in a separate subset suggest complementary roles for these biomarkers. Overall, findings support a neurocristopathic model of tumor development in TSC and LAM and identify potential biomarkers and therapeutic targets beyond mTOR inhibition.

## Abstract 2

We identified neural crest cell lineages and the collaborative mechanisms used to drive tumor growth in tuberous sclerosis and lymphangioleiomyomatosis, revealing potential biomarkers and promising therapies.

## Graphical abstract

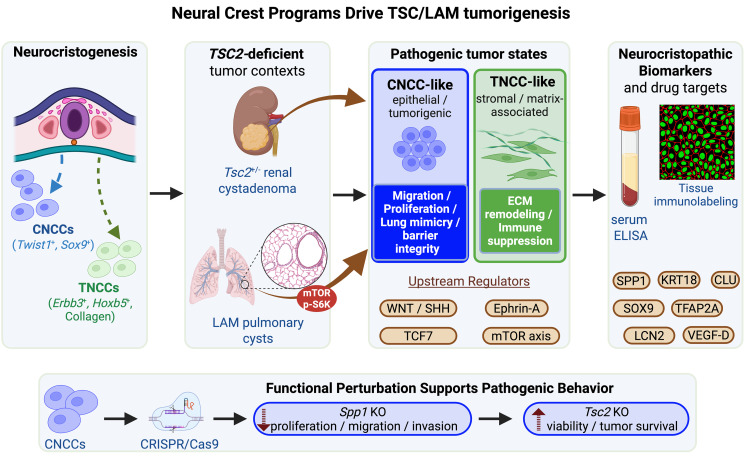

## Introduction

Tuberous sclerosis complex (TSC) is a multisystem neurocutaneous disorder resulting from loss-of-function mutations in *TSC1/TSC2* genes and dysregulation of the mTOR pathway (1). mTOR inhibitors can impede tumor growth and improve certain neurological symptoms (2, 3). However, complete eradication remains challenging (4), requiring ongoing treatment, which is associated with marked toxicity and drug interactions (5). In lymphangioleimyomatosis (LAM), the main lung manifestation of TSC, the disease often progresses after cessation of therapy, leading to functional decline (6).

Establishing the cellular origin of TSC and LAM tumors is essential for the development of targeted therapies. Although previous studies have suggested the pleural mesothelium, uterine smooth muscle, and embryonic neural crest cells (NCCs) (7, 8), a definitive lineage origin has not been established. Genetic fate mapping in the *Tsc2^+/–^* mouse model (9) using myelin protein zero-Cre–driven (*Mpz*-Cre–driven) reporters in our laboratory demonstrated increased accumulation of Cd57-expressing neural crest–derived cells in renal tumors, accompanied by increased tumor burden (10). These findings demonstrated that NCCs could contribute to TSC-related tumor development, though the precise mechanisms and functional properties involved remained unclear.

Deciphering NCC-specific pathogenic programs is complicated by the overlap between neurocristogenesis gene signatures and those of epithelial-to-mesenchymal transition (EMT), a process linked to cancer progression (11). To distinguish genuine neural crest lineage from EMT-driven mimicry and uncover therapeutic targets beyond mTOR inhibition, scRNA-seq of renal cystadenomas from *Tsc2^+/–^* mice was integrated with an analysis of human LAM pulmonary tumors, prior genetic lineage-tracing data (12), and functional CRISPR perturbation of key neurocristopathic markers in NCCs. This multimodal strategy allowed high-confidence cell type prediction, identification of cranial (CNCC) and trunk NCC (TNCC) subpopulations, definition of lineage-specific biomarkers, and delineation of signaling pathways that drive neurocristopathic tumorigenesis in both TSC and LAM.

## Results

### NCCs are key drivers of Tsc2^+/–^ mouse renal tumor development.

**Figure 1:**
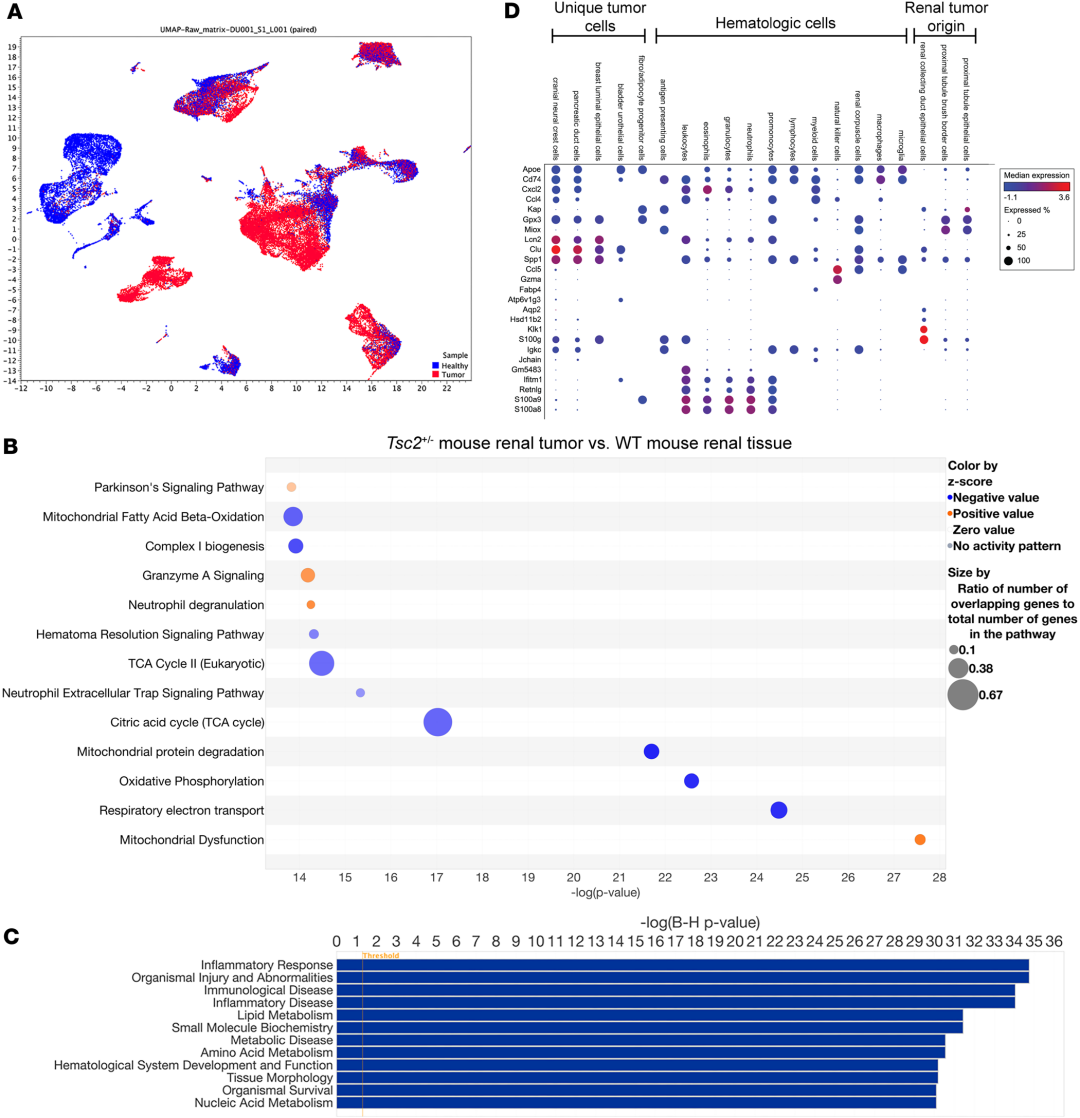
scRNA-seq analysis of renal tumorigenesis in the tuberous sclerosis (*Tsc2^+/–^*) mice. (**A**) UMAP clustering of cells from 8-month-old WT kidneys (*n* = 3 mice; 19,078 cells) and age-matched *Tsc2^+/–^* renal cystadenomas (*n* = 4 mice; 26,861 cells). (**B**) Top canonical pathways predicted to be activated (red) or inhibited (blue) in *Tsc2^+/–^* tumors versus WT kidney and ranked by significance (–log_10_ *P* value, Benjamini-Hochberg [B-H]). Bubble size indicates the proportion of significant dataset genes relative to all pathway genes. (**C**) Top 12 diseases/biofunctions predicted for *Tsc2^+/–^* tumors versus WT tissue, based on B-H corrected *P* values. (**D**) Median expression of the 25 most variable genes from scRNA-seq across 3 main cell categories — unique tumor cells, hematologic cells, and renal tissue cells. Circle size represents the percentage of gene-expressing cells, with red-to-blue hues indicating up- to downregulation. Complete cell-type expression data are shown in Supplemental Figure 1. Analyses were performed using Qiagen CLC Genomics Workbench v23.0.4 and Ingenuity Pathway Analysis (IPA) v68752261.

scRNA-seq of WT renal cortex (*n* = 3 mice; 19,078 cells) versus *Tsc2*^+/–^ renal cystadenomas (*n* = 4 mice; 26,861 cells) identified shared renal and hematopoietic cell clusters, as well as tumor-restricted populations (Figure 1A). Pathway analysis revealed activation of cell death processes — mitochondrial dysfunction (*z* = 4.62; *P* ≤ 2.7 × 10^–28^; ratio = 0.17), decreased TCA cycle activity (*z* = –3.74; *P* ≤ 9.45 × 10^–18^; ratio = 0.67) — and inflammatory responses, including Granzyme A signaling (*z* = 3.64; *P* ≤ 6.54 × 10^–15^; ratio = 0.27), and neutrophil degranulation (*z* = 4.04; *P* ≤ 5.63 × 10^–15^; ratio = 0.10) in *Tsc2*^+/–^ mice tumors (Figure 1B), indicating tissue necrosis. Gene ontology enrichment supported these findings, highlighting active nucleic acid, lipid, and amino acid metabolism processes associated with tumor cell survival and proliferation (Figure 1C).

Differential expression of the 25 most variable genes across 3 broad cell categories: renal tissue cells, hematologic/immune cells, and unique tumor cells (Figure 1D), revealed low expression of renal epithelial markers in a small percentage of cells of renal origin, reflecting tumor necrosis, as predicted in Figure 1B. Robust expression of *S100* and *Retnlg* in myeloid cells is consistent with active inflammation (Figure 1D) (13). Five unique tumor cell types — CNCCs, pancreatic duct cells, breast luminal epithelial cells, bladder urothelial cells, and fibro/adipocyte progenitors — display gene signatures atypical for the kidney and were enriched in tumor-specific clusters (Figure 1D). Expression data for all 55 resolved cell types are available in Supplemental Figure 1 (supplemental material available online with this article; https://doi.org/10.1172/jci.insight.195013DS1). Based on their high expression of key tumor-related genes (Figure 1D), we hypothesized that among these unique tumor cells, CNCCs were most likely to drive renal tumor development in *Tsc2^+/–^* mice.

**Figure 2:**
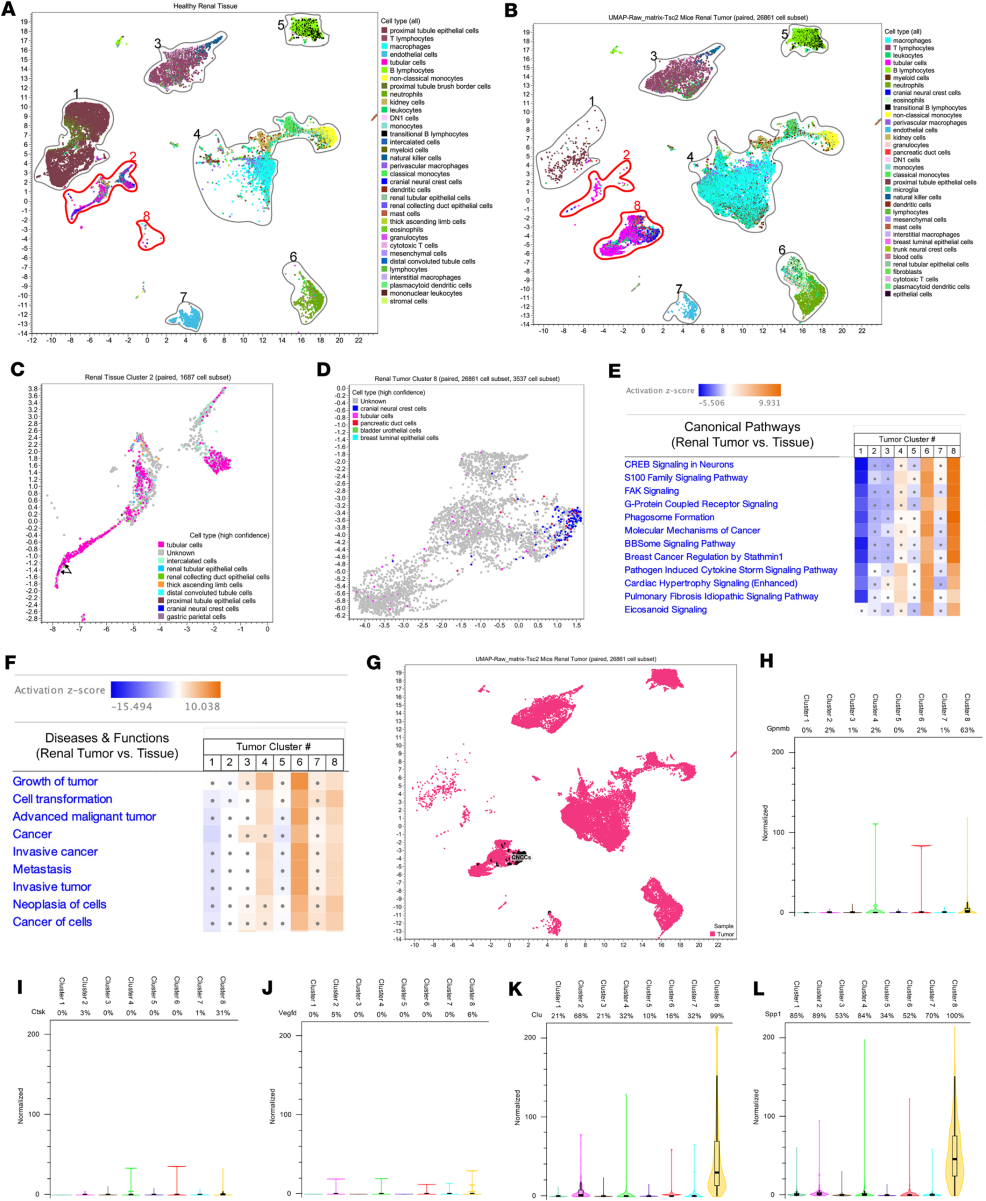
Pathogenic NCCs in *Tsc2^+/–^* mouse renal tumors. (**A** and **B**) Cell type clustering of scRNA-seq data from WT mice (**A**) and *Tsc2^+/–^* mice (**B**) based on the Platt-scaled probabilistic fit between DEGs and canonical cell type signatures curated in the Qiagen CLC gene ontology database. Cell types annotated with ≤ 50% confidence are ranked by abundance as ‘Cell Type (all)’ in the legends for **A** and **B**; cell clusters 1-8 were also predicted with high confidence (>50%; Table 1). (**C** and **D**) Magnified images show CNCCs (dark blue arrows) in Cluster 2 of WT mouse kidney (**C**), and as dark- blue hued cells in Cluster 8 of Tsc2^+/–^ renal tumors (**D**), identified with high confidence. (**E** and **F**) Intercluster analyses reveal activation of tumorigenic pathways (**E**) and biofunctions (**F**) mainly in Clusters 6 and 8, with orange and blue hues representing up- and downregulation, and gray indicating insignificant pathways. (**G**) CNCCs occur mostly in *Tsc2^+/–^* Cluster 8 (*n* = 796 low confidence; *n* = 165 high confidence). (**H**–**L**) Expression of canonical renal tumor markers — *Gpnmb* (**H**), *Ctsk* (**I**), *Vegfd* (**J**) — and neurocristopathic markers — *Clu* (**K**), S*pp1* (**L**) — with the percentage of gene-expressing cells per cluster indicated. Analyses were performed using Qiagen CLC Genomics Workbench v23.0.4 and Ingenuity Pathway Analysis (IPA) v68752261.

To test our hypothesis, we employed UMAP clustering and cell type prediction to identify differences in cell populations between *Tsc2^+/–^* mice tumors and WT tissues. Using high-confidence cell-type predictions with > 50% probability estimates via Platt scaling (14) (Supplemental Methods), decreasing populations of proximal tubule epithelial and brush border cells (Cluster 1), collecting duct and intercalated cells (Cluster 2), and endothelial cells (Cluster 7) were observed in tumors compared with the WT kidney (Table 1 and Figure 2, A and B), consistent with earlier findings (Figure 1D). Conversely, macrophages (Cluster 4) and proliferating granulocytes/agranulocytes (Cluster 6) increased in renal tumors (Table 1 and Figure 2, A and B), linked to immune and inflammatory responses in *Tsc2^+/–^* mouse renal tumors (13). Cluster 8 in renal tumors sequesters CNCCs (4.5% of cluster 8), pancreatic duct cells (0.6%), and an “unknown” group of heterogeneous cell types (93%) whose identity cannot be confidently confirmed (>50% probability) using curated cell type classifier elements in the Qiagen Cell Ontology knowledge base (Table 1 and Figure 2B). Since small populations of endogenous CNCCs exist in healthy kidneys within cluster 2 (Figure 2C), they could transform, multiply approximately 80-fold, and induce neighboring cells via paracrine signaling, to contribute to tumor development in cluster 8 (Figure 2D). This is evident when comparing CNCC fluxes in clusters 2 and 8 between renal tissue and tumors (Figure 2, A and B). Even when confidence thresholds are not considered in cell type predictions (Cell Type - All), CNCCs still rank as the second most abundant cell type in cluster 8 of *Tsc2^+/–^* tumors, with CNCC-derived mesenchymal stem cells also represented (Supplemental Figure 2).

Subsequent analysis confirmed Cluster 6 and Cluster 8 as key tumorigenic groups selectively activating classical tumorigenesis pathways, including focal adhesion kinase (FAK) signaling (6.6 ≤ *z* ≥ 9.3), molecular mechanisms of cancer (7.11 ≤ *z* ≥ 8.85), and S100 family signaling (6.9 ≤ *z* ≥ 9.1) (Figure 2E). Analyzing the downstream effects of these cancer-related gene signatures further implicates cell clusters 4, 6, and 8 in driving tumor phenotypes in *Tsc2^+/–^* mice (Figure 2F). Given the established identity and inflammatory roles of cells in Cluster 4 and Cluster 6, these findings further support our postulate that CNCCs in cluster 8 drive tumor formation in *Tsc2^+/–^* mice. Furthermore, CNCCs are selectively enriched in Cluster 8 of *Tsc2^+/–^* tumors (Figure 2G) — being among the most abundant annotated NCC lineage in this cluster, with and without probabilistic thresholds (Supplemental Figure 2) — where most cells expressed markers of mouse renal tumorigenesis, including glycoprotein NMB (*Gpnmb*) (Figure 2H), cathepsin K (*Ctsk*) (Figure 2I), and *Vegfd* (Figure 2J). Additionally, highly variable CNCC genes in *Tsc2^+/–^* mouse renal tumors (Figure 1D), including clusterin (*Clu*) (Figure 2K) and osteopontin (*Spp1*) (Figure 2L), were primarily expressed by Cluster 8 tumor cells. Pancreatic duct cells demonstrated a 25-fold increase in Cluster 8 (Figure 2D); future studies will explore their role in tumor development in *Tsc2^+/–^* mice and in TSC disease.

### Identification of neurocristopathic markers in Tsc2^+/–^ mouse renal cystadenomas.

**Figure 3:**
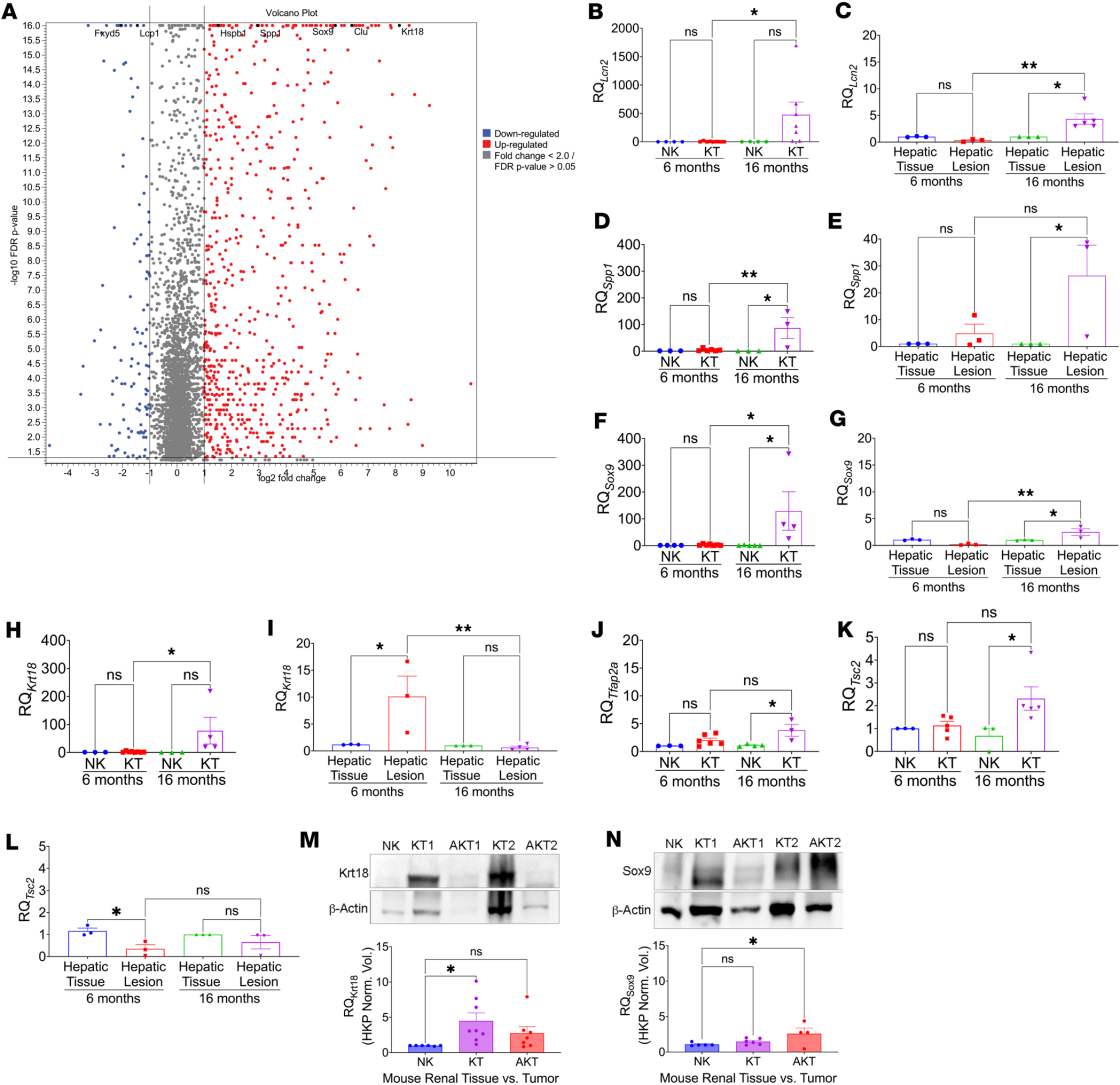
Neurocristopathic markers of renal tumorigenesis in *Tsc2*^+/–^ mice. (**A**) DEGs between CNCCs and other cell types in *Tsc2^+/–^* mouse renal tumors, highlighting highly regulated neural crest markers. (**B**–**I**) The complete list of 824 DEGs in CNCCs is provided in Supplemental Table 2. qPCR validation of CNCC markers in renal and hepatic tumors from *Tsc2^+/–^* mice versus age-matched WT tissues at 6 and 16 months, including *Lcn2* (**B** and **C**), *Spp1* (**D** and **E**), *Sox9* (**F** and **G**), and *Krt18* (**H** and **I**). (**J**) *Tfap2a* expression in *Tsc2^+/–^* tumors versus WT kidneys. (**K** and **L**) Tuberin mRNA in renal (**K**) and hepatic (**L**) tissues/tumors over the same period. Each data point represents the sample mean from 3–7 mice. (**M** and **N**) Immunoblot analysis of Krt18 (**M**) and Sox9 (**N**) in kidney tumors (KT) and adjacent tissues (AKT) from 16-month-old *Tsc2^+/–^* mice (*n* = 4–8) compared with WT mice kidney (NK) (*n* = 5–6), normalized to housekeeping proteins (HKP). Each data point on graphs is a biological replicate. One-way ANOVA with Dunnett’s post hoc test; *P < 0.05, **P < 0.005.

Differential gene expression analysis of renal tumors distinguished 824 genes between CNCCs and other tumor cell types (Figure 3A) (111 downregulated, 713 upregulated), which included 30% of the top 100 genes used in annotating CNCCs (Supplemental Table 1). These include known CNCC markers and regulators of neurocristogenesis, such as SRY Box transcription factor 9 (*Sox9*), transcription factor activating protein B (*Tfap2b*), cytokeratins (*Krt18*, *Krt8*, *Krt7*), and heat shock protein family B Member 1 (*Hsbp1*) (Figure 3A and Table 2) (12). Other highly regulated genes that distinguish CNCCs from other tumor cells include *Spp1*, *Clu*, and lipocalin 2 (*Lcn2*) (Figure 3A and Table 2), which also markedly influence *Tsc2^+/–^* mouse tumor development (Figure 1D) and are thus potential candidate markers of TSC-associated neurocristopathy. The top 30 differentially expressed genes (DEGs) in CNCCs, ranked by FDR *q* and fold-change, are listed in Table 2, with a complete list in Supplemental Table 2.

The expression of selected canonical and noncanonical CNCC genes was validated in renal tumors and hepatic lesions of aging *Tsc2^+/–^* mice. *Lcn2* (Figure 3B), *Spp1* (Figure 3D), *Sox9* (Figure 3F), *Krt18* (Figure 3H), and *Tfap2a* (Figure 3J) all increased with age in these mice, exhibiting a 2- to 120-fold elevation in renal tumors between 6 and 16 months compared with healthy tissues, correlating with tumor growth (10). In hepatic lesions — less common in these mice — CNCC marker expression increased 2- to 25-fold at 16 months compared with both 6 months and age-matched healthy liver tissues (Figure 3, C, E, and G). *Krt18* was an exception, showing lower levels in hepatic lesions at 16 months (Figure 3I). *Tsc2* mRNA levels were higher in 16-month-old renal tumors (Figure 3K) but unchanged between hepatic tissues and lesions (Figure 3L). Additionally, Krt18 (Figure 3M) and Sox9 (Figure 3N) protein levels were markedly elevated in kidney tumors (KT) and tumor-adjacent tissue (AKT), respectively.

### Uncovering multilineage NCCs in LAM pulmonary tumors.

**Figure 4:**
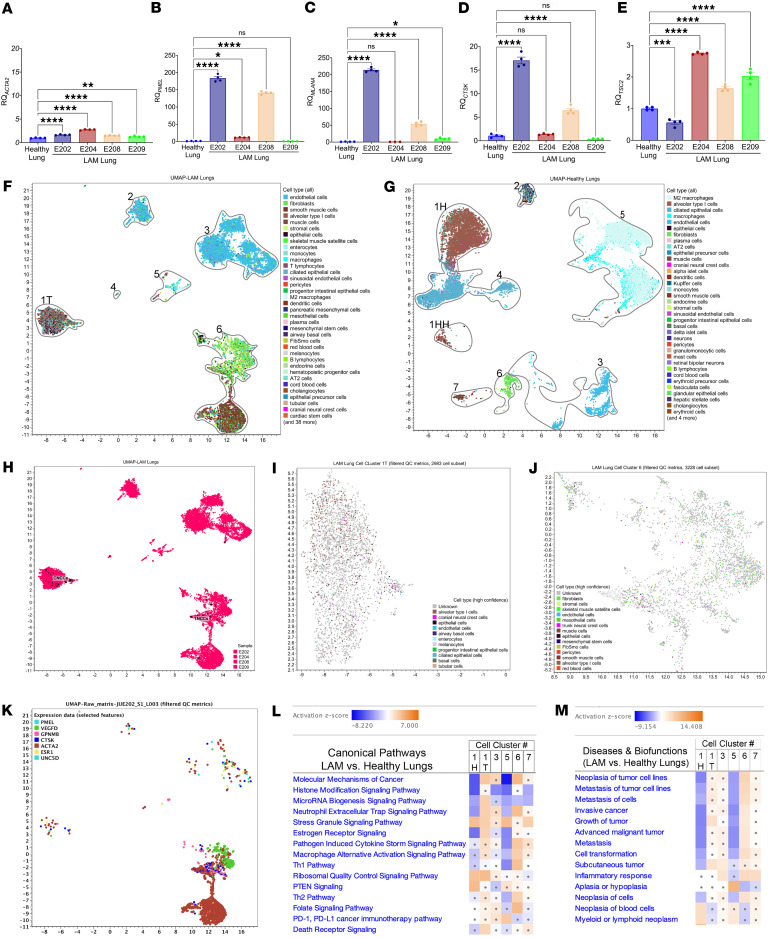
Multilineage NCCs in LAM pulmonary tumors. (**A**–**E**) qPCR validation of known LAM markers: *ACTA2* (**A**), *PMEL* (**B**), *MLANA* (**C**), *CTSK* (**D**), and *TSC2* (**E**), in fresh pulmonary tumor samples from 4 deidentified patients with LAM (E202, E204, E208, E209). (**F** and **G**) Cell type identification in pooled LAM tumors from scRNA-seq (17,551 cells) (**F**), and healthy lung data from LungMAP (Accession #LMEX0000004396; LungMAP.net) (4 donors, 27,093 cells) (**G**) based on gene expression linked to cell type signatures curated in the Qiagen CLC knowledge base. Legends rank “all cell types” identified with a confidence probability ≤ 50% by decreasing tissue abundance. (**H**) CNCCs occupy LAM tumor cluster #1T (*n* = 124 low confidence; *n* = 30 with high confidence), while TNCCs predominate Cluster 6 (*n* = 44 low confidence; *n* = 17 high confidence). (**I** and **J**) Magnified views of CNCCs in Cluster 1T (**I**) and TNCCs in Cluster 6 (**J**) in magenta. (**K**–**L**) Greater marker diversity and distribution observed in Clusters 1T and 6, which harbor NCC subtypes (**K**), are predicted to facilitate the activation of prooncogenic signaling pathways (**L**) and biological functions (**M**). Orange and blue hues represent up- and downregulation, respectively, and gray dots indicate insignificance. Analyses were performed using the Qiagen CLC Genomics Workbench v23.0.4 and Ingenuity Pathway Analysis (IPA) v68752261.

Pulmonary neoplasms from 4 de-identified patients with LAM (E202, E204, E208, E209) (Supplemental Table 3) sourced from the National Disease Research Interchange (NDRI), were processed within 48 hours of surgery and validated for LAM markers (8). All tumors exhibited elevated α-smooth muscle actin (*ACTA2*) compared with healthy lung tissue (Figure 4A), and selectively expressed premelanosome (*PMEL*), melanoma antigen recognized by T cells (*MLANA*), and *CTSK* (Figure 4, B–D). Notably, E202’s lung tumor exhibited markedly reduced *TSC2* mRNA levels (Figure 4E), indicating variability in mTOR pathway activation among tumors, consistent with prior findings (15).

To identify tumor-initiating NCCs and their signaling pathways, LAM lung single-cell data were integrated with healthy lung data from the LungMAP.net repository (*n* = 4 female donors; 27,093 cells; accession#: LMEX0000004396) (16). Transcriptomic analysis showed minor clustering differences between healthy and LAM lungs, particularly in the spatial distribution of cell types within Cluster 1T (LAM tumors), Cluster 1H, and Cluster 1HH (healthy lungs), likely due to sample heterogeneity and varying protocols. Nonetheless, the same cell types were present in both healthy lungs and LAM neoplasms (Figure 4, F and G, and Supplemental Table 4). Two NCC subtypes — CNCCs (Figure 4, H and I) in Cluster 1T, and TNCCs (Figure 4, H and J) in Cluster 6 — were identified in LAM tumors based on lineage signature genes (*Wnt1*^TOM+^ CNCCs, Supplemental Table 1; *Sox10*^TOM+^ TNCCs, Supplemental Table 5) (12), though their numbers were up to 5 times lower than in *Tsc2^+/–^* mouse renal tumors. Markers of the recently identified LAM-core cells — proposed as the cells of origin (8) — such as *PMEL*, *VEGF-D*, and the Unc-5 netrin receptor D (*UNC5D*), showed greater diversity in clusters 1T and 6, associated with CNCCs and TNCCs, respectively (Figure 4K). These findings link LAM pathogenicity to the NCC subtype distributions within tumors.

High-confidence cell type predictions determined a substantial reduction in M2-type macrophages (Cluster 5) and alveolar type 1 epithelial cells (Cluster 1H and Cluster 1HH versus Cluster 1T) as well as an absence of detectable alveolar type 2 cells in LAM pulmonary tumors compared with healthy lungs (Figure 4, F and G, and Supplemental Table 4). Resident healthy lung CNCCs (72 cells in Cluster 1H and Cluster 4; Figure 4G) decreased in LAM tumors (30 cells in Cluster 1T and Cluster 4; Figure 4, F and I). These CNCCs, which persist into adulthood, give rise to pulmonary neurons that innervate airway smooth muscle, mucus glands, and myofibroblasts (17) and serve as progenitors for neuroendocrine cells and neuroepithelial bodies involved in chemo-sensing and repair (18). The reduction of CNCCs in Cluster 1 of LAM tumors suggests potential oncogenic transformation of this lineage. Conversely, cell types such as endothelial cells (Cluster 3), fibroblasts, stromal cells, skeletal muscle satellite cells, and TNCCs (Cluster 6), as well as smooth muscle cells (Cluster 7), increased in LAM tumors (Figure 4F and Supplemental Table 4).

To identify cell clusters with oncogenic potential, we compared intercluster differential gene expression between LAM pulmonary neoplasms and healthy lung tissue. Results were gated by *z* scores (*z* ≤ –2.0 or *z* ≥ 2.0) reflecting concordance between observed gene expression states and curated activating or inhibitory gene networks in Qiagen’s Ingenuity Pathway Analysis (IPA) knowledge base. A right-tailed Fisher’s exact test (*P* < 0.05) assessed enrichment of signaling pathways and biofunction gene sets in healthy versus LAM samples. The heatmap in Figure 4L reveals activation of classical tumorigenic pathways such as the Molecular Mechanisms of Cancer (*z* = 2.56–3.32) and Neutrophil Extracellular Trap Signaling (*z* = 2.22–3.11) in Cluster 1T (CNCCs) and Cluster 6 (TNCCs), indicating a proinflammatory tumor microenvironment. In Cluster 1T, activation of Estrogen Receptor Signaling (*z* = 2.19, *P* = 0.02) and MicroRNA Biogenesis (*z* = 2.2, *P* = 0.001) (Figure 4L) suggests estrogen-driven CNCC transformation. Cluster 6 dictates immune responses, symbolized by Th2 activation (*z* = 2.9, *P* = 0.03), while Folate Signaling (*z* = 3.21, *P* < 0.0001), indicates enhanced proliferation and survival in stem-like cells (Figure 4L). Upregulation of PTEN in healthy lung Cluster 1H reflects its tumor-suppressor role and the baseline state of normal lung tissue (Figure 4L) (19). Signaling pathways linked to tumor formation, invasion, and metastasis are primarily active in Cluster 6, then Cluster 1T (Figure 4M), supporting the postulate that NCCs induce tumorigenesis in LAM lungs.

### Pathogenicity of KRT18-expressing NCCs in LAM pulmonary tumors.

**Figure 5:**
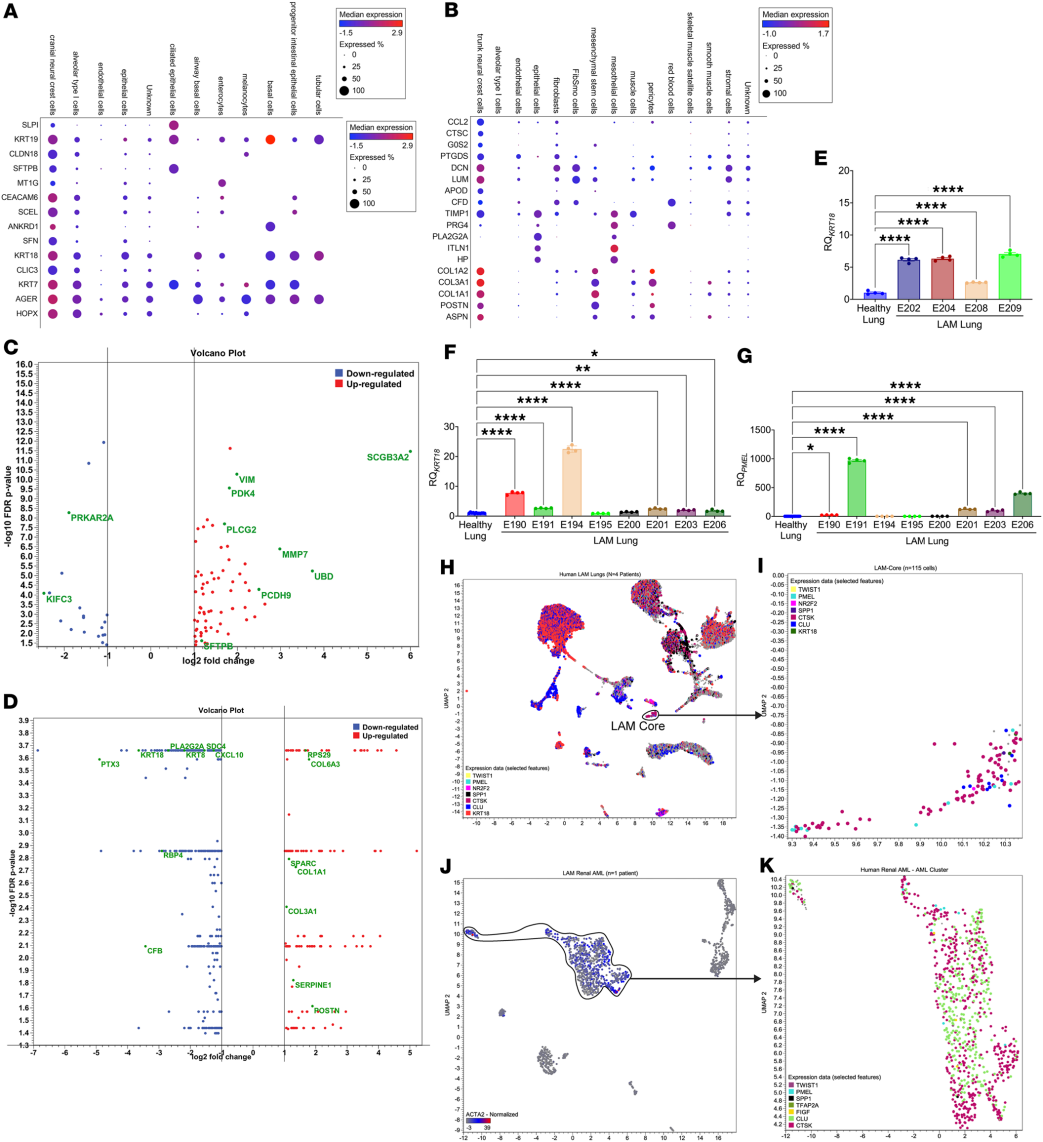
Neurocristopathic markers of LAM pulmonary neoplasms. (**A** and **B**) Median expression of the most variable genes in confidently predicted cell types (probability > 50%) within cluster #1T harboring CNCCs (**A**) and cluster #6 containing TNCCs (**B**), of LAM neoplasms (*n* = 4 patients; 17,551 cells). Dot plots rank genes by variance from highest to lowest, and circle size denotes the percentage of cells expressing each gene; colors from blue to red indicate down- to up-regulation. (**C** and **D**) Volcano plot distributions of DEGs between CNCCs and other cells in Cluster 1T (**C**) and TNCCs versus other cells in Cluster 6 (**D**). Some neurocristopathic genes of significance (FDR *q* < 0.05; log_2_FC ≥ 1.0; mean ≥ 0.5) are sampled in green. Complete lists of DEGs in CNCCs (90: 70 upregulated, 20 downregulated) and in TNCCs (472: 126 upregulated, 346 downregulated) are available in Supplemental Tables 6 and 7, respectively. (**F** and **G**) qPCR validation of KRT18 as a neurocristopathic biomarker in pulmonary neoplasms from 2 LAM patient cohorts, E202–E209 (*n* = 4) (**E**) and E190–E206 (*n* = 8) (**F**), compared with the canonical LAM marker PMEL (**G**). Statistics were performed using a 1-way ANOVA and Dunnett’s post hoc test for assays run in quadruplicate; *P < 0.05, **P < 0.005, ****P < 0.0001. (**H** and **I**) Recapitulative analysis of scRNA-seq data culled from the LAM Cell Atlas (34) and Gene Omnibus (GSE135851), comparing healthy lung (*n* = 6) to LAM pulmonary neoplasms (*n* = 4), reveal coexpression of LAM neurocristopathic markers (*SPP1*, *CLU*, *KRT18*) along with classical LAM (*PMEL*, *CTSK*, *VEGF-D/FIGF*, *MLANA*), and NCC (*TWIST1*, *NR2F2*, *TFAP2A*) markers. (**J** and **K**) Analogous marker representation in healthy kidney (*n* = 1) versus LAM renal AML (*n* = 1). Identification of LAM-Core cells (*n* = 115) (**I**) and *ACTA2^+^* pathogenic subpopulations (*n* = 853 cells) (**K**). Analyses utilized Qiagen CLC Genomics Workbench v23.0.4.

To understand how NCC lineages contribute to neoplastic transformation in LAM, we compared gene expression between CNCCs and TNCCs and other cell types within their respective clusters. CNCCs in Cluster 1T exhibit a distinctive gene signature characterized by increased mechano-sensitivity (*ANKRD1*) (20), epithelial plasticity and progenitor/alveolar-intermediate programs (*KRT19*, *KRT18*, *KRT7*, *HOPX*, *CEACAM6*, *AGER*) (21–23), alongside reduced differentiation and alveolar epithelial barrier integrity (*CLDN18*, *SFTPB*, *SCEL*, *CLIC3*) (24) (Figure 5A). This transcriptional pattern describes lineage infidelity and alveolar mimicry as the key pathogenic mechanisms of CNCCs in *Tsc2*-dependent LAM tumorigenesis. CNCC-derived tumor cells thus utilize epithelial-mesenchymal plasticity to adopt a “pseudo-alveolar” identity, supporting survival and lung niche remodeling. Conversely, TNCCs in Cluster 6 (Figure 5B) show coordinated downregulation of genes of the immune microenvironment, creating a “cold tumor” with suppressed or polarized macrophage and immune cell recruitment (*CCL2*, *PTGDS*, *CFD*, *G0S2*, *APOD)* (25, 26), and unchecked protease activity (*CTSC*, *TIMP2*) (27, 28). Concurrently, genes for collagen biosynthesis (*COL1A2*, *COL3A1*, *COL1A1*) (29), matrix-remodeling proteoglycans (decorin [*DCN*], lumican [*LUM*]) (30, 31), and desmoplasmic drivers (periostin [*POSTN*], asporin [*ASPN*]) (32, 33) were all upregulated.

After filtering for DEGs with significance thresholds (FDR *q* < 0.05, |Log2Fold Change|≥1, Max. Group Mean ≥0.5), 90 CNCC genes (70 upregulated, 20 downregulated; Supplemental Table 6) and 472 TNCC-specific genes (126 upregulated, 346 downregulated; Supplemental Table 7) were identified. Spatial distribution of genes distinguishing CNCCs in Cluster 1T (Figure 5C) and TNCCs in Cluster 6 (Figure 5D) is shown in volcano plots, which facilitated the identification of lineage-specific neurocristopathic markers involved in LAM tumorigenesis — highlighted in green. Notably, the high variability in *KRT18* expression was a defining feature of CNCCs within cell cluster 1T (Figure 5A). The gene also ranked fifth among DEGs in TNCCs as captured in Figure 5D (FDR-q = 2.18 × 10^–4^, Fold Change = –12.48, Max. Group Mean = 7.01 Supplemental Table 7). Given that *Krt18* was also implicated in CNCC-induced growth of aging *Tsc2^+/–^* mouse tumors, akin to aging LAM pathology, its endemic role in these disorders is apparent.

To gauge the pathogenicity of this neurocristopathic marker in TSC and LAM, its expression was validated in LAM pulmonary tumors. *KRT18* significantly increased across all 4 LAM samples (E202, E204, E208, E209) compared with healthy lung tissue (Figure 5E). In 8 additional de-identified LAM cases, 6 showed higher *KRT18* levels (Figure 5F), and 5 had elevated *PMEL* (Figure 5G). These results identify KRT18 as a neurocristopathic tumor marker in both TSC and LAM. scRNA-seq data from the LAM Cell Atlas, the central hub for LAM patient data in the regional United States (34), validated the expression of *KRT18*, *SPP1*, and *CLU* in LAM pulmonary tumors and their LAM-core cell subpopulation (Figure 5, H and I) (8). *ACTA2*^+^ cells in renal AML, linked to LAM pathogenicity, also expressed these markers (Figure 5, J and K). Moreover, the tandem expression of canonical LAM markers (*PMEL*, *CTSK*, *FIGF* [*VEGF-D*], *MLANA*) and neural crest markers (*TWIST1*, *NR2F2*, *TFAP2A*) strongly supports a neurocristopathic component of TSC and LAM.

### Evidence of neurocristopathy in LAM pulmonary tumors.

**Figure 6:**
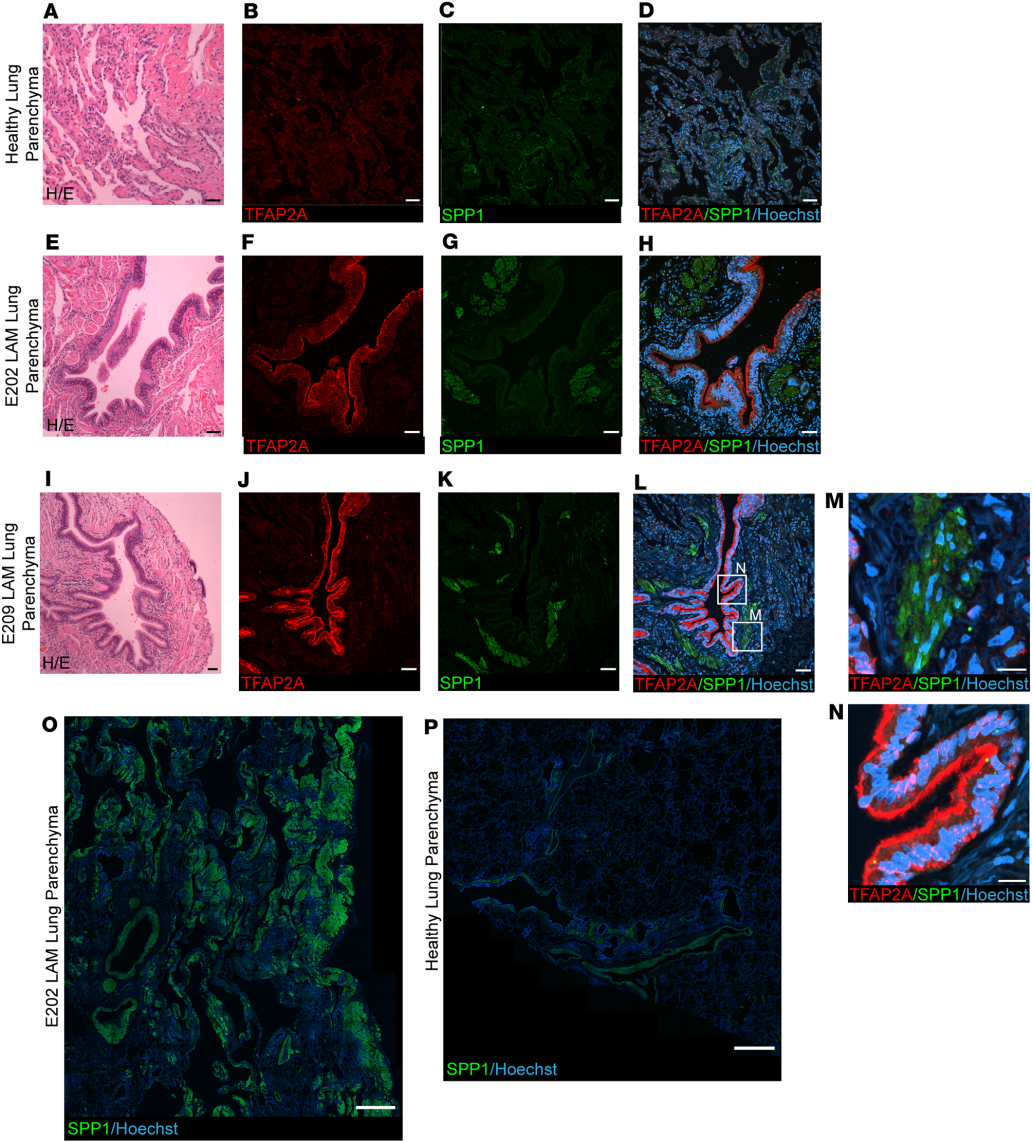
Evidence of neurocristopathy in LAM pulmonary neoplasms (SPP1 and TFAP2A). (**A**–**D**) Healthy lung parenchyma expresses minimal SPP1 restricted to smooth muscle regions bordering airway epithelia, and expression of the neural crest marker TFAP2A was not observed. (**E**–**P**) By contrast, TFAP2A is expressed at the apical membrane and in cilia of airway epithelia in patients with LAM E202 (**E**–**H**) and E209 (**I**–**N**), while SPP1 expression extends into the lung parenchyma and alveoli, as revealed by wide-area confocal imaging of E202’s pulmonary neoplasm (**O**) versus healthy donor lung (**P**). Boxed regions in **L** are enlarged in **M** and **N**. IHC results for patients E204 and E208 are provided in Supplemental Figure 3, while control slides can be visualized in Supplemental Figure 14. Hoechst 33342 served as a nuclear counterstain. Scale bars: 20 μm (magnified images [**M**, **N**]); 50 μm (**A**–**N**); 500 μm (wide-area confocal images [**O**, **P**]). Imaging was performed using the Nikon TiE Eclipse or AXR MP resonant scanning (wide-area imaging) confocal microscopes.

IHC for established and candidate neurocristopathic markers in pulmonary neoplasms from the 4 NDRI patients with LAM (E202, E204, E208, and E209) versus age-matched healthy lungs showed minimal SPP1 and no TFAP2A expression (Figure 6, A–D). By contrast, neoplasms from patients E202 (Figure 6, E–H) and E209 (Figure 6, I–N) exhibited strong TFAP2A localization at the apical membrane and cilia of airway epithelial cells, with moderate cytoplasmic expression. SPP1 expression was markedly increased in tumor-associated smooth muscle, and in E202, extended throughout the lung parenchyma and alveoli (Figure 6O), unlike healthy lungs (Figure 6P), as revealed by wide-area confocal microscopy.

**Figure 7:**
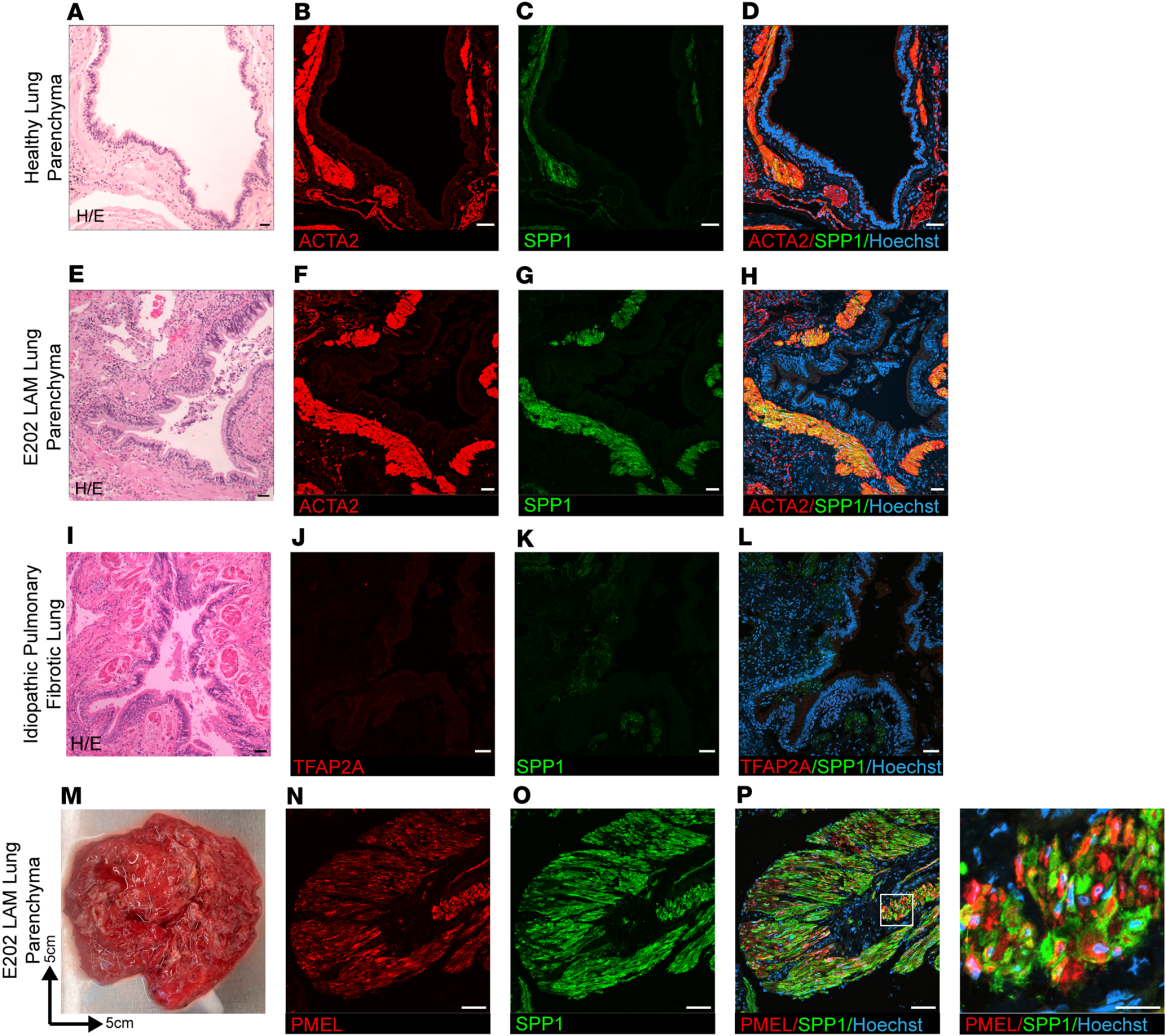
Evidence of neurocristopathy in LAM pulmonary neoplasms (SPP1 and ACTA2, PMEL). (A–D) SPP1 colocalizes with ACTA2 in healthy lung tissue. (**E**–**H**) Marked increase in expression of both markers in E202’s LAM lung neoplasm. (**I**–**L**) Limiting SPP1 expression and no TFAP2A was observed in age-matched idiopathic pulmonary fibrotic (IPF) lungs, akin to expression patterns in healthy lungs. (**M**) Sample of E202’s neoplastic lung. (**N**–**P**) Colocalization of SPP1 with PMEL, a premelanosome LAM marker, in the smooth muscle layer. Boxed region in **P** is magnified in the adjacent panel. SPP1/ACTA2 double-labeling IHC results for patients E204, E208, and E209 is provided in Supplemental Figures 4 and 5 while SPP1/PMEL results can be visualized in Supplemental Figure 6. Control tissue slides for comparison are collated in Supplemental Figure 15. Hoechst 33342 served as a nuclear counterstain. Scale bar: 20 μm (magnified images), 50 μm (**A**–**P**). Images were captured using a Nikon TiE Eclipse confocal microscope.

These findings were confirmed by the colocalization of ACTA2 and SPP1 at low levels in healthy lungs (Figure 7, A–D), contrasted with marked overexpression in overlapping regions within patient E202’s lungs (Figure 7, E–H). Similar results were validated in tumors from patients E204, E208, and E209 by TFAP2A/SPP1 IHC (Supplemental Figure 3), and ACTA2/SPP1 IHC (Supplemental Figures 4 and 5). Specificity for LAM neurocristopathy was further supported by the lack of TFAP2A and relatively low SPP1 expression, restricted to bronchiolar smooth muscle in an idiopathic pulmonary fibrotic (IPF) lung (Figure 7, I–L). SPP1 also colocalized with PMEL in tumor smooth muscle from E202 (Figure 7, M–P) and E208 (Supplemental Figure 6). Although PMEL was undetectable in cystic lungs from patients E204 and E209 (Supplemental Figure 6), consistent with qPCR results (Figure 4B), SPP1 and ACTA2 were elevated across all LAM patient tumors.

**Figure 8:**
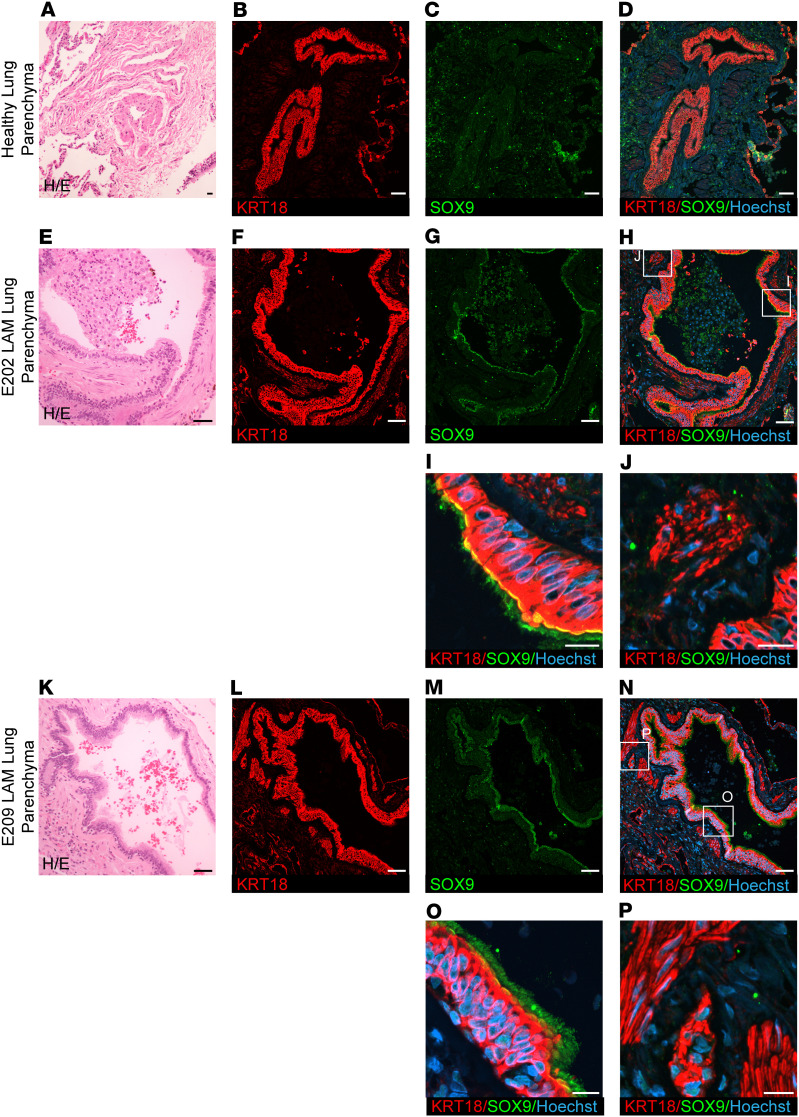
Evidence of neurocristopathy in LAM pulmonary neoplasms (KRT18 and SOX9). (**A**–**D**) KRT18 expression was observed only in airway epithelia in healthy lung parenchyma, while SOX9 was absent. (**E**–**P**) By contrast, KRT18 expression extended from the airway epithelia to adjacent smooth muscle and beyond in patients E202 (**E**–**J**) and E209 (**K**–**P**) LAM lungs, while SOX9 localized to the apical membrane and cilia. Boxed regions in **H** and **N** are magnified in panels **I**, **J**, **O**, and **P**, respectively. IHC results for patients E204 and E208 can be visualized in Supplemental Figure 7 and control tissue slides in Supplemental Figure 15. Hoechst 33342 served as a nuclear counterstain. Scale bars: 20 μm (magnified images [**I**–**J**; **O**–**P**]); 50 μm (**A**–**H**; **K**–**N**). Images were captured using a Nikon TiE Eclipse confocal microscope.

**Figure 9:**
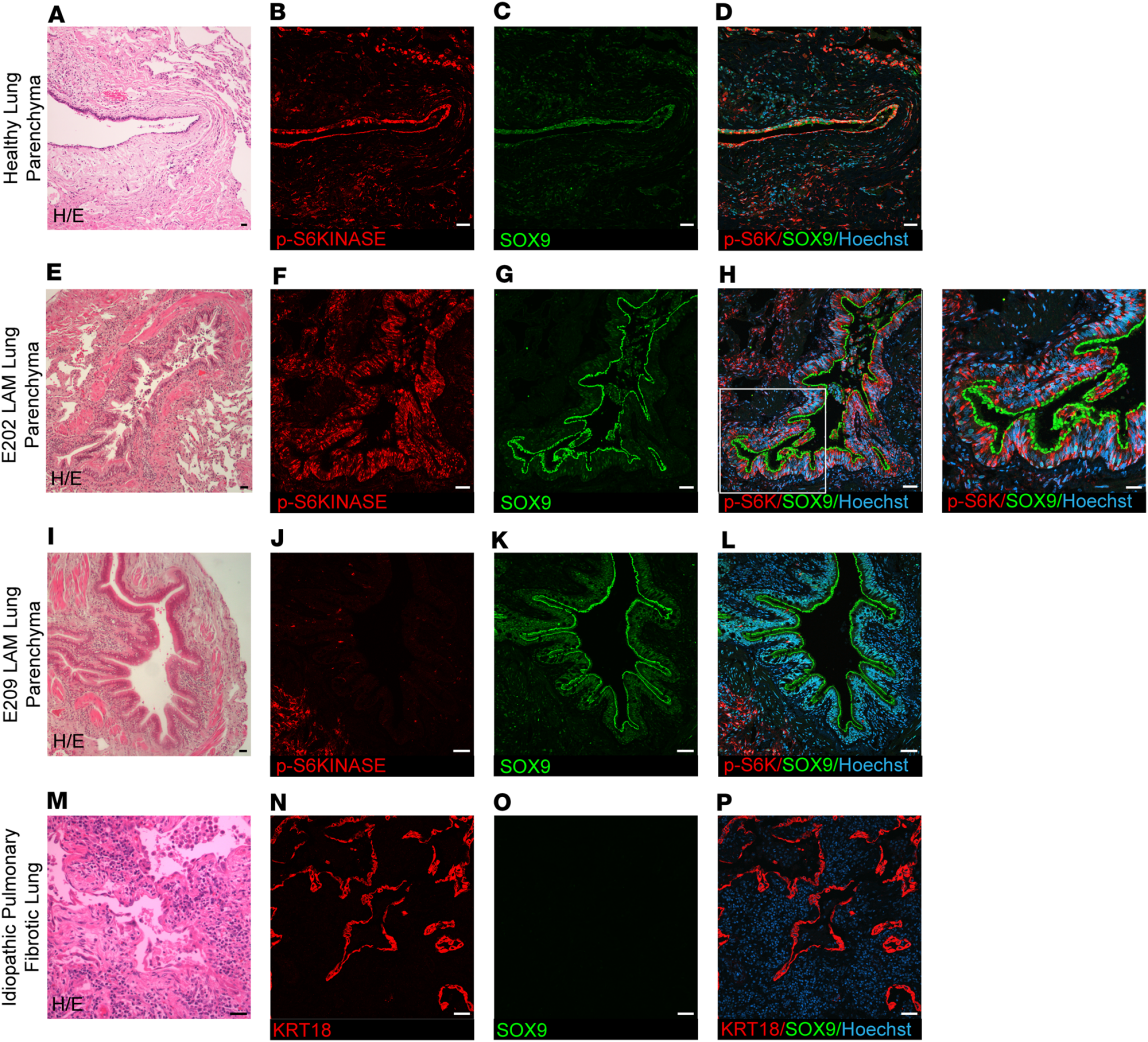
Evidence of neurocristopathy in LAM pulmonary neoplasms (SOX9 and p-S6K). (**A**–**D**) Healthy lungs lack SOX9 but minimally express phospho-p70S6Kinase (T389) (p-S6K), an indicator of mTOR pathway activation. (**E**–**H**) SOX9 localizes to the apical membrane and cilia in E202’s LAM lung with widespread p-S6K overexpression. (**I**–**L**) E209 LAM lungs similarly coexpress both markers but show less active mTORC1. Double immunolocalization results for SOX9 and p-S6K in patients E204 and E208 are provided in Supplemental Figure 8. The E209 LAM lung parenchyma field shown in panel 9I is identical to that in Figure 6A and was reused here to illustrate double-label IHC for SOX9 and p-S6K. Independent sections from all 4 patients with LAM showed comparable SOX9/p-S6K colocalization patterns (Supplemental Figure 8). (**M**–**P**) Comparative KRT18/SOX9 double immunolabeling in an age-matched IPF lung. The boxed region in **H** is magnified in the adjacent panel. Control tissue slides are shown in Supplemental Figure 15. Hoechst 33342 served as a nuclear counterstain. Scale bars: 20 μm (magnified images), 50 μm (**A**–**P**). Images were captured using a Nikon TiE Eclipse confocal microscope.

In healthy lung parenchyma, KRT18 expression was confined to bronchiolar airway epithelial cells and SOX9 was undetectable (Figure 8, A–D). In contrast, pulmonary neoplasms from patients, E202 (Figure 8, E–J), E209 (Figure 8, K–P), and E204 and E208 (Supplemental Figure 7) exhibited moderate ciliary SOX9 colocalized with KRT18 in airway epithelia and adjacent smooth muscle. Healthy lung tissue showed minimal SOX9 and phosphorylated p70S6K (p-S6K) colocalization (Figure 9, A–D), indicating low-level mTOR pathway activation, whereas LAM lungs from E202 (Figure 9, E–H), E209 (Figure 9, I–L), and E204 and E208 (Supplemental Figure 8) exhibited moderate to high SOX9 expression at the membrane and cilia of cystic and bronchiolar airway epithelia, colocalized with p-S6K. These expression patterns distinguished LAM from healthy and IPF lungs (Figure 9, M–P), and paralleled PMEL/SPP1 colocalization in cystic LAM lungs (Figure 7).

### Evidence of neurocristopathy in renal neoplasms.

Analogous expression of neurocristopathic and canonical NCC markers was observed in *Tsc2^+/–^* mouse renal cystadenomas (Supplemental Figures 9 and 10) and LAM renal angiomyolipoma (AML) (Supplemental Figures 11 and 12).

### Candidate neurocristopathic markers show promise for LAM diagnostics.

**Figure 10:**
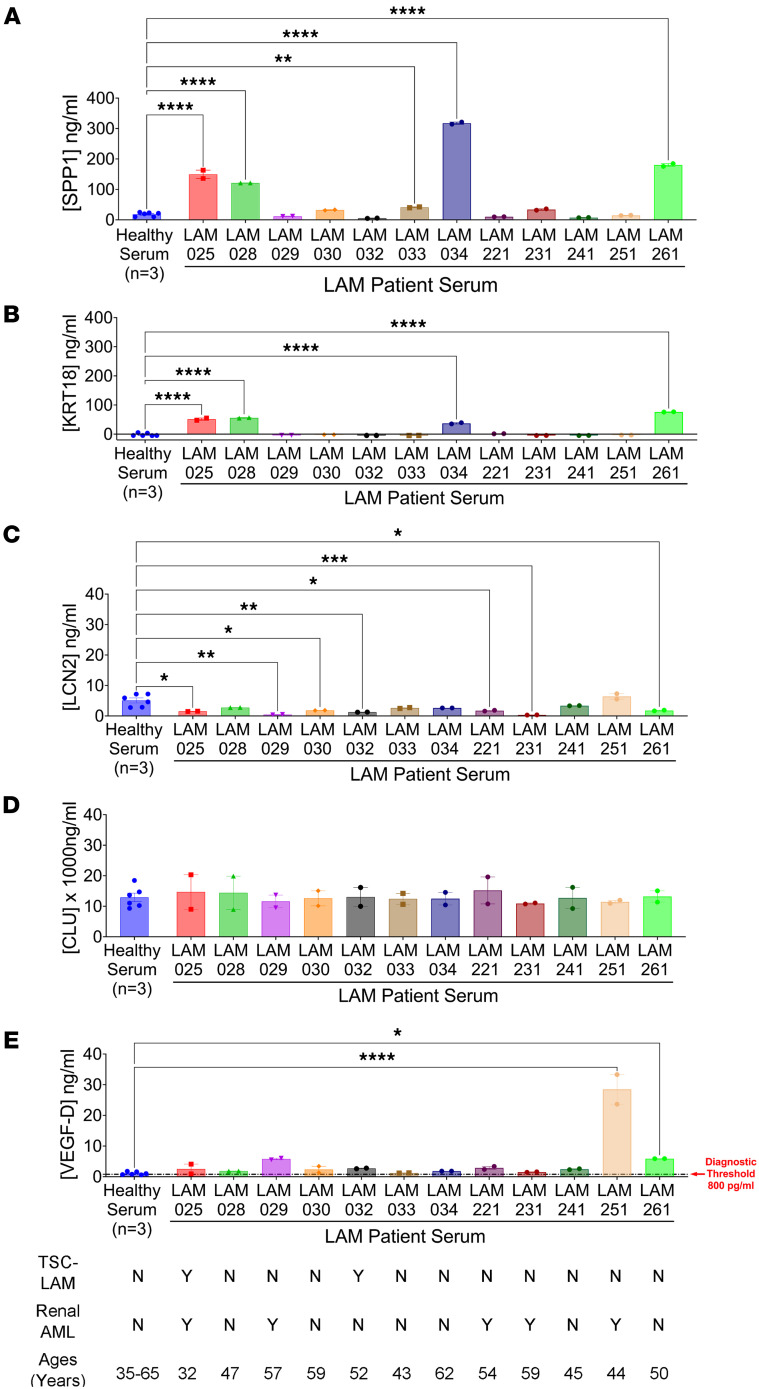
ELISA detection of neurocristopathic markers in LAM patient serum. (**A**–**E**) Serum concentrations of SPP1 (**A**), KRT18 (**B**), LCN2 (**C**), CLU (**D**), and VEGFD (**E**) in deidentified patients with LAM (*n* = 12) were compared with their levels in healthy donor serum (*n* = 3) via ELISA. In **E**, the diagnostic threshold for VEGFD is indicated with a dashed line. Incidence of *TSC2* mutations and renal AML per patient is tabulated. Assays were performed in duplicate per sample. One-way ANOVA with Dunnett’s post hoc test was used for statistics; *P < 0.05, **P < 0.005, ***P < 0.001, ****P < 0.0001.

ELISA of serum samples from 12 patients diagnosed with TSC-LAM or sporadic LAM, versus 3 age-matched healthy controls showed a 10- to 18-fold higher serum SPP1 in 5 patients (Figure 10A), with KRT18 elevated in 4 of these 5 patients (Figure 10B), supporting their diagnostic value for specific LAM phenotypes. Conversely, lower LCN2 identified disease in 11 patients, reaching statistical significance in 7 (Figure 10C), consistent with immunohistochemical findings (Supplemental Figure 12), whereas CLU levels remained unchanged (Figure 10D). Although VEGFD exceeded the 800 pg/mL diagnostic threshold in most patients (Figure 10E) (35), it was significantly elevated in only 2 of the 12 patients and showed limited overlap patients exhibiting increased neural crest markers, suggesting that VEGFD reflects a distinct aspect of the LAM pathology.

### Genetic recombination studies recapitulate NCC physiology associated with tumorigenesis.

**Figure 11:**
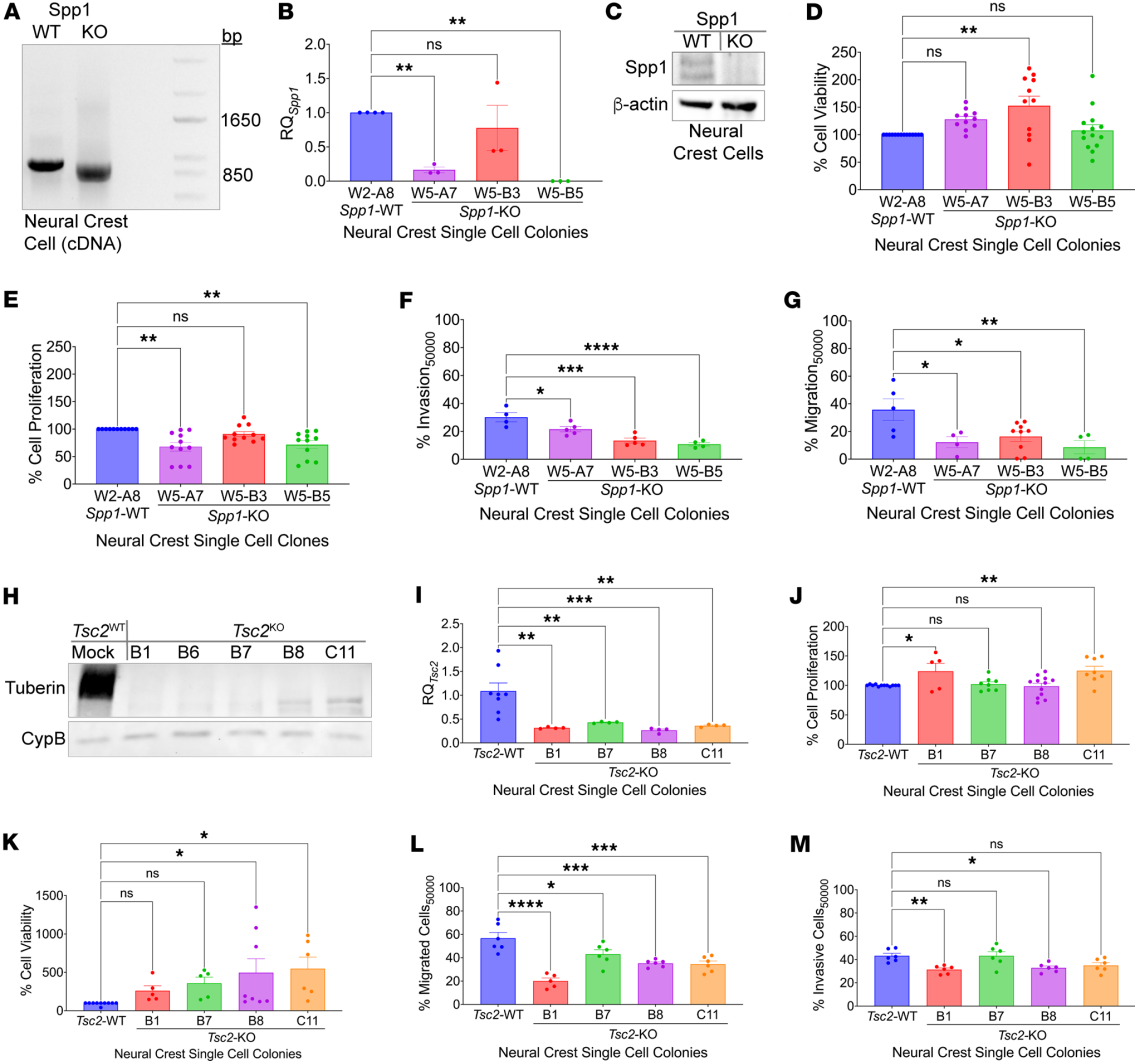
Tumorigenic effects of inhibiting *Spp1* and *Tsc2* expression in mouse CNCCs. (**A**) CRISPR-based edits targeting exon 4 of *Spp1* in 09-1 mouse CNCCs were confirmed by a PCR product size change at 935 bp. (**B**) qPCR validation of *Spp1* suppression in CNCC clones (W5-A7, W5-B3, W5-B5) with KO-score/(insertion/deletion) (Indel%) of 94/(100%), 46/(48%), and 88/(88%), respectively. (**C**) Protein verification of Spp1-KO in CNCC clone W5-B5. (**D**–**G**) *Spp1*-KO did not affect clone viability, as measured by ATP luminescence (**D**) but significantly reduced CNCC proliferation (28%–33%) (**E**), invasion (29%–64%) (**F**), and migration (54%–76%) (**G**) compared with unedited W2-A8 CNCCs. Boyden chamber assays were initiated with 50,000 CNCCs per well and yielded similar results at lower cell densities (Supplemental Figure 13). (**H**) PCR confirmation results of CRISPR-mediated *Tsc2* KO in exon 6 of 09-1 CNCCs in clones B1, B6, B7, B8, and C11 with KO-score/(Indel%) of 94/(94%), 92/(92%), 93/(93%), 93/(94%), and 92/(92%), respectively. (**I** and **J**) qPCR validation of *Tsc2* downregulation (**I**), leading to a 25% proliferative increase in clones B1 and C11 (**J**). (**K**) ATP levels increased 3- to 6-fold across clones, indicating enhanced viability that may promote tumor survival and resistance. (**L** and **M**) *Tsc2*-KO minimally reduced migration (**L**) and invasion (**M**) in most clones. Results obtained using lower cell densities (10,000 cells/well) are described in Supplemental Figure 13. Each data point on the graphs represents the average across triplicate or quadruplicate wells per experiment, with at least 3 repeats. Statistics were performed using a 1-way ANOVA with Dunnett’s post hoc test; *P < 0.05, **P < 0.01, ***P < 0.0005, ****P < 0.0001.

To assess the role of *Spp1* in NCC physiology, CRISPR-mediated targeting of *Spp1* exon 4 in the 09-1 mouse CNCC line, which express Spp1 up to 106-fold higher than in non-NCC tissues (36), was detected by PCR (Figure 11A). (Full details regarding the origin of this CNCC line are provided in the Methods section and Supplemental Methods subsection of the Supplemental Data.) Gene recombination yielded 3 mutant clones (W5-A7, W5-B3, W5-B5) with KO scores and insertion/deletion percentages (Indel%) of 94/100%, 46/48%, and 88/88%, respectively. qPCR confirmed reduced *Spp1* expression in clones W5-B5 and W5-A7 (Figure 11B), and decreased Spp1 protein in W5-B5 was verified by Western blots (Figure 11C). Functional assays over 24 hours revealed that *Spp1* KO did not affect NCC viability (Figure 11D) but reduced proliferation by 28%–33% only in W5-A7 and W5-B5 (Figure 11E), correlating with ICE scores. All 3 KO clones significantly decreased invasion by 29%–64% (Figure 11F) and migration by 54%–76% (Figure 11G) relative to nonedited W2-A8 NCCs, at both high (50,000 cells/well) and low (10,000 cells/well) (Supplemental Figure 13) seeding densities. Therefore, *Spp1* supports proliferative and motile NCC phenotypes associated with tumorigenesis.

To model NCC transformation leading to LAM and TSC tumors, CRISPR/Cas9-mediated recombination at Tsc2 exon 6 in 09-1 CNCCs generated 5 mutant clones — B1, B6, B7, B8, and C11 — with respective KO-score/indel% of 94/94%, 92/92%, 93/93%, 93/94%, and 92/92%. *Tsc2* downregulation was confirmed by Western blot (Figure 11H) and qPCR (Figure 11I). Only clones B1 and C11 showed a 25% increase in proliferation (Figure 11J), whereas all clones assessed exhibited a pronounced 3- to 6-fold increase in viability over nonedited NCCs (Figure 11K). Effects on NCC chemotaxis and invasion were minimal or modestly inhibitory (Figure 11, L and M). These results suggest that *Tsc2* mutations during neurocristogenesis promote NCC survival where as *Spp1* more strongly drives tumorigenic motility and proliferation, which account for TSC and LAM neurocristopathy by a nonproliferative increase in tumor viability, consistent with the benign yet persistent nature of TSC and LAM tumors.

### Pathway analysis reveals developmental and metabolic reprogramming in CNCC-derived renal tumors.

**Figure 12:**
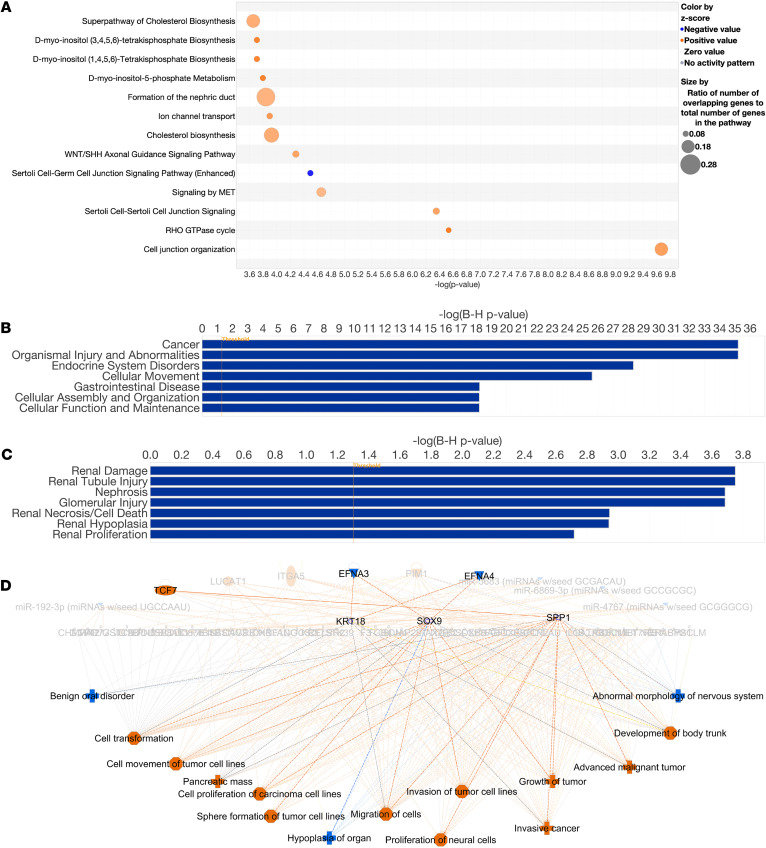
Delineating mechanisms of neurocristopathic tumorigenesis in *Tsc2^+/–^* mouse renal cystadenomas. (**A**) Top signaling and metabolic pathways predicted to be activated (orange) or inhibited (blue) in CNCCs in *Tsc2^+/–^* mouse renal tumors (*n* = 796 cells). Bubble size reflects the ratio of significantly expressed genes in CNCCs relative to all pathway genes. (**B** and **C**) Diseases (**B**) and toxicology apparatus (**C**) predicted to be activated by DEGs unique to CNCCs. (**D**) Excerpt of the third-ranked regulator effect network, with transcription factor 7 (*Tcf7*) as a key upstream regulator of *Spp1* in CNCCs linked to renal tumorigenesis. This network achieved a consistency score of 20,352, which quantifies the alignment between upstream regulators, CNCC DEGs, and their downstream phenotypes (Supplemental Table 8). Other upstream regulators include ephrin-A ligands (*Efna3*, *Efna4*), which indirectly target *Sox9* and *Krt18*, and when downregulated, inducing immune infiltration. Solid lines indicate direct interactions; dashed lines represent indirect ones. Orange and blue symbols denote predict activation and inhibition events, respectively.

DEGs distinguishing CNCCs from other cell types in *Tsc2^+/–^* mouse renal tumors (824 DEGs; Figure 3A and Supplemental Table 2) were analyzed using Qiagen IPA knowledge base. Significance was assessed by right-tailed Fisher’s exact test (*P* < 0.05). CNCC-enriched genes mapped to activated pathways crucial for NCC migration and often reactivated in cancer (37, 38), including the RHO GTPase cycle (*z* = 3.89; *P* = 1.0 × 10^–6.5^), cholesterol biosynthesis (*z* = 2.45; *P* = 1.0 × 10^–3.92^), Sertoli cell-Sertoli cell junction signaling (*z* = 2.71; *P* = 1.0 × 10^–6.35^), and cell junction organization (*z* = 2.83; *P* = 1.0 × 10^–9.7^) (Figure 12A). WNT/SHH axonal guidance signaling (*z* = 2.67; *P* = 1.0 × 10^–4.28^), which supports neural crest specification and migration (39), and nephric duct formation (*z* = 2.24; *P* = 1.0 × 10^–3.84^), an embryonic urogenital developmental program (40), were strongly upregulated. These changes indicate reactivation of developmental programs not characteristic of homeostatic adult renal epithelium (Figure 12A) (40, 41). Consistent with this, most enriched pathways were activated, demonstrating that CNCCs in TSC renal tumors retain core developmental signaling networks governing cytoskeletal dynamics, cell-cell and cell-extracellular matrix communication, migration, epithelial-mesenchymal plasticity (EMT/MET), and metabolic reprogramming (42). Gene ontology enrichment further links CNCC signatures to cancer, hormonal dysfunction, and glomerular injury (Figure 12, B and C). These results provide transcriptomic evidence that CNCCs in TSC renal tumors activate both broad cancer-associated pathways and developmentally unique neural crest programs, supporting their role as true neural crest derivatives.

Next, IPA regulator effect analysis of CNCC DEGs prioritized candidate neurocristopathic markers associated with tumorigenicity and identified regulator-disease/function networks ranked by consistency scores. Figure 12D shows a segment of the third-ranked regulator effect network, centered on *Spp1*, *Sox9*, and *Krt18*. The complete list of regular effect networks, their target molecules in the CNCC DEG dataset, and the predicted downstream effects on diseases and functions — whether activated or inhibited — is provided in Supplemental Table 8. Notably, transcription factor 7 (*Tcf7*) emerged as a positive upstream regulator of *Spp1* linked to tumor malignancy (Figure 12D). Ephrin-A ligands (*Efna3* and *Efna4*) were predicted to negatively regulate *Sox9* and *Krt18*, indirectly promoting tumor growth via *Sox9* (Figure 12D). However, the role of Ephrin A-*Krt18* signaling in TSC renal tumorigenesis remain unexplored and warrant further study.

## Discussion

This study presents genetic, transcriptomic, and functional evidence that tumors associated with TSC and LAM possess cranial (CNCC) and trunk (TNCC) neural crest–derived lineages that bifurcate into distinct neoplastic and stromal tumor compartments. NCC signatures were confirmed by IHC in mouse and human tumors and supported by serum profiling of candidate neurocristopathic markers. Functional links to TSC and LAM pathogenesis were established through CRISPR-mediated manipulation of *Spp1* and *Tsc2* in primary CNCCs. Collectively, the findings support a unified neurocristopathic model whereby *TSC1/2* loss in neural crest lineages drives tumor development in TSC and cystic lung remodeling in LAM by leveraging developmental plasticity and metabolic reprogramming, as well as ECM remodeling and mechano-transduction processes.

The studies demonstrate that CNCCs, rare in healthy kidneys, expand approximately 80-fold in *Tsc2*^+/–^ tumors and cluster with pancreatic duct–like cells and fibro/adipocyte progenitors (10). CNCC marker genes, including *Sox9*, *Krt7/8/18*, *Spp1*, *Clu*, and *Lcn2*, together with *Gpnmb*, *Vegfd*, and *Ctsk*, have been linked to renal tumorigenesis (43–45) and TSC/LAM (8, 46, 47). Pathway analysis of 824 CNCC-specific DEGs reveals activation of both canonical cancer-related pathways — cholesterol biosynthesis, MET signaling, and the RHO GTPase cycle (important for actin remodeling and cell junctions) (48, 49) together with neural crest–specific developmental programs — WNT/SHH and axonal guidance pathways — that govern neural crest migration during embryogenesis (50). These programs are increasingly linked to aggressive neurocristopathies (51, 52). The strong overlap with cancer-associated EMT programs (11) explains why EMT and neural crest signatures are difficult to distinguish by scRNA-seq alone and supports viewing TSC and LAM as disorders with prominent neurocristopathic signatures rather than clearly defined NCC-of-origin diseases. Overall, these pathways and regulatory signatures support a model in which *Tsc2*-deficient CNCCs sustain and co-opt neural crest–specific developmental programs, alongside common oncogenic processes, to drive migratory, invasive, and stem-like properties that promote tumor initiation and progression.

Integrative analysis of LAM lung single-cell data with reference lung atlases (16) confirmed representation of all major lung lineages but identified CNCC- (Cluster 1T) and TNCC-enriched (Cluster 6) populations within LAM nodules. LAM-CORE markers such as *PMEL*, *VEGFD*, and *UNC5D* showed heterogeneous expression across both NCC clusters, indicating that LAM-CORE cells are closely associated with CNCC/TNCC lineages rather than exclusively from lung or uterine epithelium (8). IHC revealed strong expression of TFAP2A, SPP1, SOX9, and KRT18 in airway epithelium and adjacent smooth muscle, along with high levels of ACTA2 and p-S6K. In contrast, healthy and fibrotic lungs lacked TFAP2A and SOX9 and had low SPP1, supporting neurocristopathy and mTORC1 activation in LAM (39, 53). Similar patterns in renal AML included widespread ACTA2, SPP1, and PMEL expression, with low or nonexistent LCN2 compared with normal cortex, highlighting neural crest–derived programs in extracranial TSC lesions.

Our analysis of CNCC and TNCC-associated expression programs offers mechanistic insights into LAM. In LAM lungs, CNCC-enriched clusters upregulate basal keratins (*KRT7*, *KRT8*, *KRT18*, *KRT19*), *HOPX*, and *AGER*, with concomitant loss of *CLDN18* and reduced surfactant gene expression. This transcriptional pattern resembles AT2–AT1 transitional states and *KRT8^+^* alveolar intermediate cells, key progenitors in lung regeneration, fibrosis, and adenocarcinoma initiation (22, 54, 55). Loss of *CLDN18*, a negative regulator of YAP activity in the distal lung (32, 33), removes a structural brake on progenitor expansion and transformation, supporting a model in which CNCC-derived LAM cells are neural crest–biased, alveolar intermediate–like tumor–initiating cells between epithelial differentiation and mesenchymal activation.

By contrast, TNCC-enriched clusters exhibit a stromal program marked by high *COL1A1*, *COL1A2*, and *COL3A1*, and increased proteoglycans (*DCN*, *LUM*, *POSTN*, and *ASPN*). This profile mimics *POSTN^+^/ASPN^+^* cancer-associated fibroblast (CAF) subsets in hepatocellular, kidney, and lung cancers, in which CAFs generate aligned, cross-linked collagen matrices, recruit and polarize tumor-associated macrophages, and exclude cytotoxic T cells, thereby creating stiff, immunosuppressed niches that impair immunotherapy (56). Decorin and lumican regulate collagen fibrillogenesis and growth-factor availability, whereas asporin enhances collagen cross-linking and matrix stiffness, collectively shaping ECM architecture and mechanotransduction (57). Our data suggest that TNCC-derived cells in LAM function as neural crest–derived CAFs that build a periostin- and asporin-rich collagen matrix, amplifying YAP/TAZ and mTORC1 signaling in adjacent CNCC-derived tumor cells, thereby promoting lymphovascular invasion and immune exclusion (58, 59).

The identification and increased expression of neurocristopathic biomarkers, *Spp1*, *Lcn2*, *Krt18*, and *Sox9* (except *Lcn2*), in both renal tumors and hepatic lesions of aging *Tsc2^+/–^* mice, and human LAM neoplasms, provide cross-species validation of the neurocristopathic model and extend our study beyond lineage and the microenvironment. Serum ELISAs identified elevated circulating levels of these neurocristopathic markers, including SPP1 and KRT18, with potential diagnostic value in patients distinct from those expressing VEGFD, and consistently decreased LCN2 levels compared with controls, with superior diagnostic efficacy. This is not unexpected, as VEGFD reflects aberrant lymphangiogenesis secondary to tumor-induced tissue remodeling (60) and is not indicative of NCCs. Our CRISPR/Cas9-mediated KO studies in primary CNCCs support the proposed neurocristopathic mechanisms and highlight the importance of these neurocristopathic biomarkers. *Spp1* deletion markedly decreased proliferation (28%–33%), migration (54%–76%), and invasion (29%–64%) of 09-1 CNCCs derived from the E8.5 *Wnt1*-Cre reporter mouse, aligning with data that identify *Spp1* as a key promoter of tumorigenesis (61), and confirming its role in CNCC physiology and its therapeutic potential.

Conversely, *Tsc2* deletion increased CNCC viability 3- to 6-fold with modest effects on proliferation and chemotaxis, reflecting the benign yet persistent nature of TSC and LAM tumors (62). Therefore, *Tsc2* loss appears to primarily enhance survival, with additional signals, such as SPP1-driven motility and YAP/TAZ-mediated mechano-transduction, needed for overt neoplasia (63, 64). These genotype-phenotype correlations provide mechanistic evidence that neural crest lineages harboring *Tsc2* mutations are major drivers of TSC and LAM tumor biology, a hypothesis supported by our published lineage-tracing studies (10). Additionally, SPP1 overlaps with PMEL in LAM tumors but is more abundant and consistently expressed, making it a more reliable marker than PMEL, the traditional LAM indicator. Absence of neurocristopathic markers in age-matched IPF tissues further confirms their specificity for LAM and TSC.

Our findings collectively support reframing TSC and LAM as disorders characterized by substantial neurocristopathic signaling, rather than solely as mTOR-driven cancers, and potentiate combinatorial therapies targeting mTORC1 alongside neural crest–associated pathways. The identification of serum-detectable neurocristopathic biomarkers enables noninvasive disease monitoring and patient stratification, with multiple biomarkers potentially improving diagnostic accuracy, especially in VEGFD^–^ cases. Importantly, targeting NCCs and clarifying signaling linking neurocristopathic markers to tumor development could address the limitations of mTOR inhibitor therapy for TSC and LAM. Overall, our results indicate that, in TSC/LAM tumorigenesis, *Tsc2* loss, *SPP1* induction, and intrinsic NCC developmental programs must cooperate with ECM remodeling by TNCC to form a self-reinforcing circuit involving mTORC1, YAP/TAZ, and neurocristopathic lineage identity (59).

### Study limitations.

This study has some limitations that warrant consideration. First, our previous lineage tracing with *Mpz*-Cre–TdTomato mice (10) used a constitutive, not inducible, Cre driver, which prevented precise timing of recombination and the ability to distinguish early developmental NCCs from true long-term lineage NCCs. Additionally, experiments did not directly address overlaps in neural crest and EMT gene expression in development and cancer (12, 59). Second, while high-confidence predictions classify 93% of cells in *Tsc2^+/–^* tumors as “unknown” and 4.5% as CNCCs, relaxing thresholds shows CNCCs dominate (Supplemental Figure 2). However, scRNA-seq cannot confirm their exact origin due to transitional states (65). Human lineage assignments also depended on reference atlases and marker expression rather than genetic fate mapping. Third, analyses focused only on female *Tsc2^+/–^* mice, limiting insights into sex differences in neural crest marker expression or pathway activation. Functional experiments specifically targeting CNCCs, and similar manipulations of TNCCs, as well as in vivo validation of CNCC/TNCC crosstalk, are yet to be conducted. Nonetheless, the consistent presence of neural crest signatures, developmental pathway activation, and neurocristopathic biomarkers across *Tsc2^+/–^* mice, LAM lung, AML, and serum, supports a central role for neural crest–derived lineages in organizing both the neoplastic and stromal compartments in TSC and LAM.

## Methods

### Sex as a biological variable.

All in vivo and human tissue studies utilized female *Tsc2^+/–^* mice, WT controls, and women with LAM to minimize sex-dimorphic variability in neurocristopathic marker expression and transcriptomic signatures.

### Animal studies.

B6129S4-*Tsc2*^tm1Djk^/J (*Tsc2^+/–^*) mice (strain no. 004686) and B6129SF2/J WT littermates (strain no. 101045) (The Jackson Laboratory [JAX]) were maintained at Columbia University’s Institute of Comparative Medicine (ICM) animal facility as described (66).

### Mouse renal tissue/tumor dissociation and single-cell isolation.

Renal cortical tissues (~1 cm³) from 8-month-old WT mice (*n* = 3, 1 tissue per mouse) and renal cystadenomas (35–250 mm³) from age-matched *Tsc2^+/–^* mice (*n* = 4, 3–7 tumors per mouse) were enzymatically processed for flow cytometry using previously established protocols (66). Cells at > 85% viability were verified at the Columbia Stem Cell Initiative Flow Cytometry Core and adjusted to 1,000 cells/μL for scRNA-seq.

### Pulmonary LAM single cell isolation and scRNA-seq.

Pulmonary LAM lesions (*n* = 4) obtained from NDRI were processed within 48 hours of surgery and dissociated as described (67). Viable PI(-) tumor cells were sorted by flow cytometry using analogous gating to the renal samples and transferred on ice for scRNA-seq. Mouse renal and human LAM single-cell suspensions were processed using the 10x Genomics Chromium 3′ Single Cell Gene Expression workflow at the JP Sulzberger Columbia Genome Center’s Single Cell Core Facility. Libraries were prepared with 3′ v3 chemistry (version 3; 10x Genomics; PN-1000075) and sequenced on a Novaseq 6000 system (Illumina) according to the manufacturer’s protocols.

### scRNA-seq data processing.

Cell Ranger (v5.0.1; 10x Genomics) was used with default parameters to demultiplex, align reads to GRCm38 (mouse) or GRCh38 (human) reference genomes, filter barcodes, and create count matrices which were imported into the CLC Genomics Workbench Single Cell Analysis Module (v23.0.4, Qiagen) for quality control, removing doublets/empty droplets, (FDR < 0.001), and normalization using a negative binomial generalized linear model (GLM) (68). Cell type classification used built-in Support Vector Machine–based (SVM-based) classifiers with Platt scaling (14), and differential expression was assessed using Wilcoxon rank-sum tests and GLM-based post hoc comparisons, including to identify cell type–defining marker genes. Dimensionality reduction was used to visualize cell clusters on UMAP plots.

### Annotating NCC subpopulations in Tsc2^+/–^ mouse renal tumors and in pulmonary LAM neoplasms.

The SVM-based sc_mouse_cell_type_classifier_v1.3 (CLC Genomics Workbench v23.0.4; Qiagen) was adapted to annotate cranial (CNCC) and trunk (TNCC) neural crest populations as cells expressing 40% of the top 100 CNCC (Supplemental Table 1) and TNCC (Supplemental Table 5) markers defined by Soldatov et al. (12) based on analysis of NCCs from E8.5 *Wnt1*-Cre; *R26R*^tomato+^ and E9.5 *Sox10*^CreERT2^; *R26R*^tomato+^ mouse embryo expression data (GSE129114). GLM-based differential expression testing (68) was used to compare tumors versus normal tissues, CNCC/TNCC versus other cell types, and corresponding clusters in *Tsc2*^+/–^ mouse tumors versus WT kidneys. The sc_human_cell_type_classifier_v1.2 SVM classifier was analogously trained to identify CNCC and TNCC populations in LAM tumors as cells expressing ≥ 30% of the top 100 CNCC or TNCC markers from Soldatov et al. (12), enabling GLM-based comparisons between neural crest–derived and other cell types in pulmonary tumors across 4 patients with LAM.

### Bioinformatics analysis.

IPA software (v68752261, Qiagen) was used for Core Analyses of DEGs applying right-tailed Fisher’s exact tests (*P* < 0.05) with Benjamini-Hochberg (B-H) correction to ascertain activated (*z* ≥ 2.0) or inhibited (*z* ≤ –2.0) gene networks, functions, and pathways, as well as magnitude ratios of gene expression between sample types with *P* value adjustment (FDR *q* < 0.05; –1.0 ≤ Log_2_FC ≤ 1.0) (69). A regulator effects analysis biased toward CNCC differential expression profiles to infer upstream regulators, downstream functional effects, and neurocristopathic tumorigenic networks, with ranked regulator–effect pairs, are summarized in Supplemental Table 8.

### Histology, immunohistochemistry, and imaging.

FFPE and paraformaldehyde-fixed frozen tissues (4 μm) from mouse kidney and liver, human renal cortex, LAM renal AML, healthy lung, LAM pulmonary neoplasms, and fibrotic lung were processed for H&E and immunostaining, with antigen retrieval, peroxidase blocking, and antibody staining following established protocols (10, 66). Incubation with primary and secondary antibodies, and Hoechst 33342 nuclear counterstain (10μg/mL; Thermo Fisher Scientific), followed by autofluorescence quenching for 5 min, was performed against neurocristopathic and LAM markers SPP1, TFAP2A, SOX9, KRT18, ACTA2, and gp100, with detection antibodies described in Supplemental Table 9. Appropriate positive, negative, and antibody-omission controls were processed in parallel and displayed in Supplemental Figures 14 and 15. Sections were imaged on Nikon A1 and AXR resonant (wide-area imaging) confocal systems mounted on Ti2 inverted microscopes (Nikon Instruments Inc.) using standardized laser lines, emission filters, and *z* stack acquisition parameters, including tiled scans at ~0.9 μm pixel size, with 5–7 randomized fields per section acquired using NIS-Elements (AR software version 5.0.2.01 or 6).

### Western blot.

Unless specified, protein blotting materials and equipment were obtained from Thermo Fisher Scientific. Tissues and tumors were lysed, separated on 10% SDS-PAGE, transferred to nitrocellulose, and probed with primary antibodies (Supplemental Table 10). Detection was performed using HRP-conjugated secondary antibodies (Supplemental Table 10), with ECL visualization on an iBright FL1500 system. Band intensities were normalized to housekeeping proteins (β-actin or Cyclophilin B) and analyzed by 1-way ANOVA with Dunnett’s post hoc tests.

### qPCR.

Total RNA from mouse renal and hepatic tissues/lesions and human lung parenchyma and LAM tumors was extracted using a Qiagen RNeasy Mini Kit, 1μg RNA reverse-transcribed with a High-Capacity cDNA Reverse Transcription Kit (Applied Biosystems), and analyzed by TaqMan qPCR on a QuantStudio 3 system (Applied Biosystems), using ΔΔCt normalization and predesigned TaqMan-based assays (Supplemental Table 11) for neural crest markers (*LCN2*, *CLU*, *SOX9*, *SPP1*, *TFAP2A*, *KRT18*) and canonical LAM markers (*ACTA2*, *PMEL*, *MLANA*, *CTSK*, *TSC2*) (3–8 biological specimens per group).

### NCC culture.

The 09-1 cranial NCCs (Millipore Sigma) derived from E8.5 *Wnt1*-Cre; R26R-GFP mouse embryos were maintained in culture according to established protocols (36, 70) and were passaged at 80% confluency. For serum starvation, CNCCs were cultured in embryonic fibroblast SIM (Sandoz Inbred Mouse), Thioguanine-resistant, and Ouabain-resistant (STO) feeder cell–conditioned media (ATCC), supplemented with 0.5% FBS and formulated according to established protocols (36, 70).

### Generation of Spp1- and Tsc2-KO NCC lines.

For *Spp1* disruption, Cas9 ribonucleoprotein (RNP) complexes containing 3 sgRNAs (multiguide) (Supplemental Table 12) were used to target exon 4 of *Spp1* by nucleofection into the 09-1 NCC line (sgRNA:Cas9 v/v 5:1) using a 4D-Nucleofector system (Program DS150; Lonza); transfection parameters were optimized with mouse ROSA26 sgRNA (Supplemental Table 12) and pMAXGFP controls (1 μg/μL; Lonza), and single-cell clones were screened by PCR and ICE analysis (Synthego), with loss of *Spp1* expression confirmed by qPCR and Western blot.

For *Tsc2* disruption, a GFP-tagged Cas9 D10 nickase and 3 sgRNAs (AA1, AB1, AC1) (Supplemental Table 12) targeting exons 3, 6, and 8 of *Tsc2*, respectively, were assembled into RNP complexes (sgRNA:Cas9 v/v 2:1) and nucleofected into O9-1 NCCs with an electroporation enhancer (Integrated DNA Technology [IDT]). Mutant NCCs identified by PCR and ICE analysis (Synthego) were enriched based on GFP fluorescence via flow cytometry, and single cells were sorted into Matrigel-coated (250μg/mL) 96-well plates. Clonal Tsc2-KO lines were validated by qPCR and Western blot.

### Transwell migration and invasion assays.

Invasion and migration of serum-starved (0.5% FBS) *Spp1-*KO and *Tsc2*-KO mouse CNCCs versus WT CNCCs were quantified as previously described (71) using 8 μm pore-size transwells (USA Scientific). Hoechst 33342-positive cells (10 μg/mL incubation for 30 mins) on the underside of transwells were counted in 5 fields of view per transwell (diameter = 2.2 mm; area = 0.038 cm^2^) using an inverted fluorescence microscope (Nikon) and scaled to the insert area. NCC migration is then expressed as a percentage of the original seeded cells at low (1 × 10^3^) and high (5 × 10^3^) seeding densities, across at least 3 independent experiments, each performed in triplicate or quadruplicate.

### BrdU proliferation and cell viability assay.

The effect of inhibiting *Spp1* and *Tsc2* expression on CNCC proliferation was assessed by measuring BrdU incorporation (Cell Signaling Technology), a thymidine analog, as previously described (66). Experiments were repeated on 3 separate days in quadruplicates. Viability was assessed by ATP content using a luminescence assay (Promega) in quadruplicate wells (2,500 NCCs per well) per genotype, and the assay was repeated at least 3 times.

### ELISA.

Serum from healthy volunteers (*n* = 3; age 35–70) and age-matched female patients with LAM (*n* = 12; age 35–70) was analyzed for SPP1, KRT18, CLU, LCN2 (Thermo Fisher Scientific), and VEGF-D (Abcam) using commercial ELISA kits in duplicate, according to the manufacturer’s protocols.

### Statistics.

Migration, invasion, proliferation, and viability rates were quantified using Fiji (Version 2.14.0) for automated cell counting and analyzed by unpaired 2-tailed Student’s *t* tests for comparisons between Spp1-KO and WT NCCs, with *P* < 0.05 considered significant. A 1-way ANOVA with Dunnett’s post hoc tests was adopted for comparisons between healthy and LAM samples, and 2-tailed paired *t* tests for age-dependent differences in NCC marker expression, with all graphs generated in GraphPad Prism (Version 10.3.1). Data are shown as mean ± SEM.

### Study approval.

Animal studies received approval from the IACUC at Columbia University Medical Center (CUMC) (protocol no. AC-AABF2559). Human tissue use was permitted with informed consent and approved by the IRB (protocol no. AAAR7738).

### Data availability.

Single-cell RNA-seq data from WT and *Tsc2^+/–^* mouse renal tissues and tumors, and human LAM pulmonary neoplasms are publicly available in GEO under accession nos. GSE291150 and GSE292058, respectively. Neural crest training datasets were obtained from GSE129114 (12), with the analyzed data available at https://pklab.med.harvard.edu/ruslan/neural.crest.html All graph data points are detailed in the Supporting Data Values file.

## Author contributions

UJU and JMD conceptualized the study, designed methods, and secured funding. UJU and EBG performed in vitro and in vivo experiments. UJU and NR drafted the initial manuscript and performed all data analyses. UJU, NR, and JMD revised the manuscript. JMD recruited patients with LAM and provided clinical data and samples. All authors have read and approved the manuscript.

## Conflict of interest

The authors have declared that no conflict of interest exists.

## Funding support

This work is the result of NIH funding, in whole or in part, and is subject to the NIH Public Access Policy. By accepting this federal funding, the NIH has been granted the right to make the work publicly available in PubMed Central.

- NIH grant NIH/NHLBI-HL167137-01 (to JMD and UJU)
- Tuberous Sclerosis Alliance Award no. 990240 (to UJU)
- Congressionally Directed Medical Research Programs Award no. W81XWH-18-1-0077 (to JMD and UJU)
- Center for LAM and Rare Lung Disease, Columbia University Medical Center (to JMD)

## Supplementary Material

Supplemental data

Unedited blot and gel images

Supporting data values

## Figures and Tables

**Table 1:**
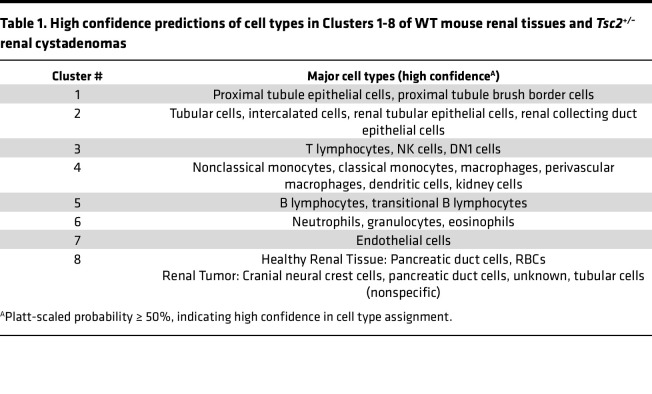
High confidence predictions of cell types in Clusters 1-8 of WT mouse renal tissues and *Tsc2^+/–^* renal cystadenomas

**Table 2:**
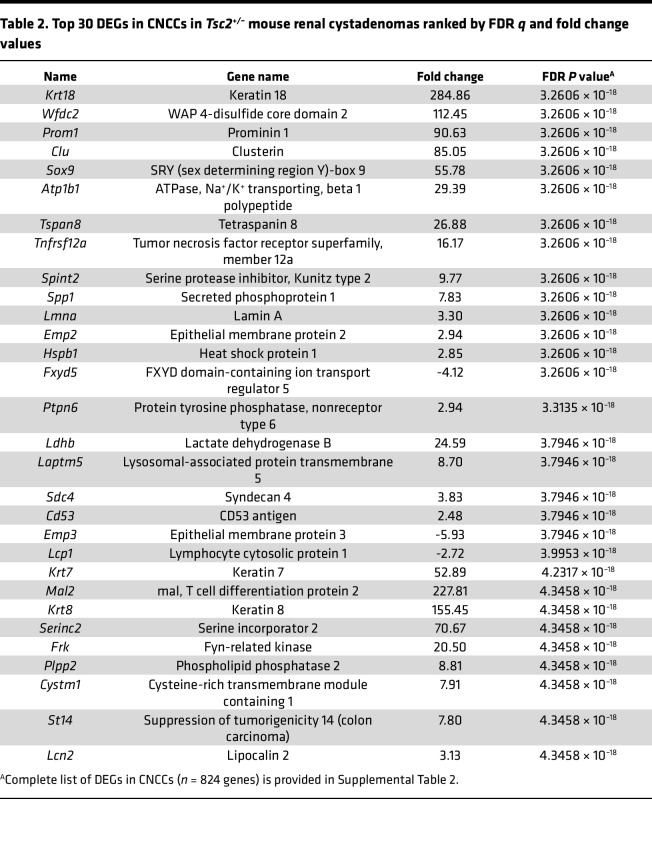
Top 30 DEGs in CNCCs in *Tsc2^+/–^* mouse renal cystadenomas ranked by FDR *q* and fold change values

## Version 1 06/04/2026

In-Press Preview

## Version 2 07/22/2026

Electronic publication
