# Supplementary material for: Neural crest cell signatures drive tumorigenesis in tuberous sclerosis complex and lymphangioleiomyomatosis
Source: JCI Insight. 2026 Jun 2;11(14):e195013. doi: 10.1172/jci.insight.195013 (PMC13461175; doi:10.1172/jci.insight.195013)
Supplement: Supplementary file 1 [file jciinsight-11-195013-s254.pdf]

Neural Crest Cell Signatures Drive Tumorigenesis in Tuberous Sclerosis Complex and Lymphangioleiomyomatosis

Authors and Affiliations

Uchenna J. Unachukwu[1], Enio C. Garcia[1], Nooralam Rai[2], Jeanine M. D'Armiento[1]

[1]Center for LAM and Rare Lung Disease, Department of Anesthesiology, College of Physicians and Surgeons, Columbia University Medical Center, 630 West 168th Street, New York.

[2]Department of Pediatrics (Pulmonary), Columbia University Medical Center, 3959 Broadway, New York.

Corresponding Author: Uchenna J. Unachukwu. Center for LAM and Rare Lung Disease, Department of Anesthesiology, College of Physicians and Surgeons, Room 7-430, Columbia University Medical Center, 630 West 168th Street, New York, NY 10032, U.S.A. Phone: 212-305-7757; Fax: 212-305-1188; Email: uu2110@cumc.columbia.edu.[1]

Conflict-of-Interest Statement: The authors have declared that no conflict of interest exists.

Supplemental Data

SUPPLEMENTAL METHODS

General

Animal studies received approval from the IACUC at Columbia University Medical Center (CUMC) (protocol #AC-AABF2559). Human tissue use was permitted with informed consent and approved by the IRB (protocol #AAAR7738).

All in vivo and human tissue studies utilized female specimens to minimize sex-dimorphic variability in neurocristopathic marker expression and transcriptomic signatures. B6129S4-*Tsc2*^{tm1Djk}/J (*Tsc2*$^{+/-}$) mice (Strain #004686) and B6129SF2/J WT littermates (Strain #101045) (The Jackson Laboratory (JAX)) were maintained at the Columbia University's Institute of Comparative Medicine (ICM) animal facility as described (1).

Animal Studies

Female B6129S4-*Tsc2*^{tm1Djk}/J (*Tsc2*$^{+/-}$) mice (Strain# 004686) and B6129SF2/J WT littermates (Strain# 101045) (The Jackson Laboratory) were bred, genotyped according to JAX protocols using PCR on tail snip DNA with *Tsc2* primers: 5'-CAAACCCACCTCCTCAAGCTTC-3' (forward) and 5'-AGACTGCCTTGGGAAAAGCG-3' (reverse) (Integrated DNA Technologies), and aged until >6 months when renal cystadenoma volumes typically exceed ~100 mm^3 (1).

Mouse Renal Tissue/Tumor Dissociation and Single-Cell Isolation

Renal cortical tissues (~1cm^3) were harvested from 8-month-old WT mice (n=3, one tissue per mouse) and renal cystadenomas (35-125 mm^3) from age-matched *Tsc2*$^{+/-}$ mice (n=4, 3-7 tumors per mouse). Tissues were rinsed twice with ice-cold PBS, minced with sterile razors, and digested in a mix of 2 mg/ml collagenase I (≥125U/mg; Cat# LS004194, Worthington Biochemical), 10 µg/ml DNase I (10 U/µl; Cat# 4716728001, Millipore Sigma), and 10 mM EDTA (Cat# E5134, Millipore Sigma) in DMEM (Cat# 30-2002, ATCC), shaken at 300 rpm for 60 min at 37°C with trituration every 30 min. Suspensions were centrifuged at 300 × g for 3 min, resuspended in RBC lysis buffer (Cat# sc-296258, Santa Cruz Biotechnology) for 10 min, then neutralized with cell sorting media (PBS with 0.5% FBS) (FBS; Cat# A3160502, Gibco, Thermo Fisher Scientific). After centrifugation for 10 min at 300 × g at 4°C, the tissues were resuspended in cell-sorting media, filtered through a 40 µm strainer, and counted. Viability dye SYTOX Green (1:1000 (v/v); Cat# S7020, Invitrogen-Thermo Fisher Scientific) was added, and samples were incubated for 10 min on ice in the dark. Flow cytometry was performed using a BD FACS Aria II (Becton Dickinson) at 20 psi with a 100 µm nozzle. Single cells were gated on FSC-A/SSC-A and FSC-A/FSC-H using unstained controls and 200,000+ events per sample; SYTOX green signals were acquired with a 530/30 nm filter. Viable cells (SYTOX$^{(-)}$) were sorted into tubes pre-coated with media and kept on ice. Cell

viability (>85%) was verified using trypan blue exclusion, and cell concentrations were adjusted to 1,000 cells/μL before sequencing.

Dissociation of Pulmonary LAM Lesions and Single Cell Isolation

Pulmonary lesions from four LAM patients were obtained from the National Disease Research Interchange (NDRI). Samples (5 cm × 5 cm × 5 cm) were immersed in DMEM with 1% penicillin-streptomycin (Penn-Strep; Cat#15140122, Thermofisher Scientific) and processed within 48 hours, with some modifications (2). After rinsing in serum-free, ice-cold DMEM/F12 (Cat# 11039-047, Thermofisher Scientific), non-malignant tissue and necrotic areas were removed. Tumors were mechanically fragmented and enzymatically digested in DMEM with collagenase I (0.2 mg/ml; Cat# LS004194), collagenase II (0.1 mg/ml; Cat# LS004174) and elastase (0.075 U/ml, Cat# LS002294) (Worthington Biochemical), DNase I (0.03 mg/ml; (Cat# 4716728001, Millipore Sigma), and 1% Penn-Strep on a shaker at 37°C for 45 minutes, then for 30 minutes after trituration. Tissue suspensions were filtered through 100- and 70-μm nylon cell strainers, centrifuged at 300 × g for 5 minutes, and erythrocytes lysed with RBC lysis buffer for 10 minutes. Cells were washed twice with DMEM/F12 + 10% FBS + 1% Penn-Strep, filtered through a 40 μm strainer, centrifuged again, and resuspended in cell sorting media (PBS with 0.5% FBS). Propidium iodide (PI) dye (1:500 (v/v)); Cat# P3566, Invitrogen-Thermo Fisher Scientific) was added for 15 minutes on ice, and flow sorting was performed to isolate viable pulmonary LAM tumor cells using a 610/20 nm filter. PI(-) cells were sorted into tubes containing cell-sorting media for sequencing.

Single Cell RNA Sequencing (scRNA-seq)

The cell suspensions underwent RNA sequencing at the JP Sulzberger Columbia Genome Center's Single Cell Core Facility. A 10x Genomics 3’ Single Cell Gene Expression (GEX) workflow (10x Genomics) was used. Gel bead partitioning and cellular barcoding were performed with the 10x Chromium Controller (10x Genomics), and libraries were constructed using Illumina Single Cell 3’ v3 chemistry following the protocol (version 3; 10x Genomics; PN-1000075). Libraries were pooled and sequenced on a NovaSeq6000 system (Illumina).

scRNA-seq Data Processing

Cell Ranger (v5.0.1; 10x Genomics) was used to demultiplex, generate FASTQ files, align reads to GRCm38 (mouse) or GRCh38 (human) reference genomes, and create count matrices. These matrices were further processed in CLC Genomics Workbench (v23.0.4, Qiagen) for quality control, removing doublets, empty droplets, and low-quality barcodes (FDR $p<0.001$), followed by normalization using a negative binomial generalized linear model (GLM) fitted to 2000 genes sampled across the expression range. Cell types were predicted using Qiagen reference classifiers in the CLC Genomics

Workbench, which generated cell clusters and annotations. The classifier employs Support Vector Machine (SVM) algorithms that use log-transformed maximum gene expression per cell and weighted training classes to distinguish cell types. Platt scaling (3) converts the decision values into probabilities, with values above 50% indicating high confidence in cell-type assignment. Probabilities below 50% mark the cell type as "unknown." Predictions without probability considerations are labeled "Cell Type (All)." Differential expression (DE) analysis was performed using Wilcoxon rank-sum tests to identify marker genes defining cell types. Post-hoc tests for differential expression between sample types (healthy vs. tumor) were performed using a minimum of 10 cells expressing a gene as the threshold. Dimensionality reduction was used to visualize cell clusters on UMAP plots. DE results were exported for pathway analysis using Qiagen's IPA software.

Annotating Neural Crest Cell Subpopulations in *Tsc2*$^{+/-}$ Mouse Renal Tumors and Pulmonary LAM Neoplasms

NCC subpopulations were annotated using SVM-based cell type classifiers in the CLC Genomics Workbench Single Cell Analysis Module. For mouse data, we configured the sc_mouse_cell_type_classifier_v1.3, trained on 234 cell types across 720 biological samples, to identify CNCCs as any cell expressing ≥40% of the top 100 differentially expressed CNCC genes resolved from work by Soldatov et al. (GSE129114) (4), who derived NCCs from the anterior cranial region of embryonic day E8.5 *Wnt1*-Cre; *R26R*$^{tomato+}$ mice. TNCCs were defined as cells expressing ≥40% of the top 100 differentially expressed genes in *Sox10*-expressing cells sorted from the trunk of E9.5 *Sox10*^{CreERT2}; *R26R*$^{tomato+}$ embryos (GSE129114) (4). These gene sets were chosen from the authors' detailed analyses of mouse cranial and trunk neural crest lineages, isolated by TdTomato-based flow cytometry during peak *Wnt1* activity (4). The top 100 CNCC and TNCC genes (Supplemental Tables, 1 and 5) are ranked by F-test p-values (Benjamini-Hochberg corrected) for expression differences between TdTomato^{+} NCC clusters and other clusters. The modified cell type classifier was then applied to normalized expression data from *Tsc2*$^{+/-}$ mouse renal tumors and WT mouse tissues to predict cell types and perform GLM-based differential expression tests (5) to distinguish *Tsc2*$^{+/-}$ mouse renal tumors versus WT tissues, matched cell clusters, and CNCCs and TNCCs versus other cell types in both tumors and tissues.

For human pulmonary LAM neoplasms, we trained the sc_human_cell_type_classifier_v1.2 (CLC Genomics Workbench v23.0.4; Qiagen) on 148 human cell types from 474 biological samples to define CNCCs and TNCCs as cells expressing ≥30% of the top 100 differentially expressed CNCC and TNCC genes, respectively (4) (Supplemental Tables, 1 and 5), compared to other cell types. The modified SVM classifier was then used to predict cell types in four LAM patient tumors (E202, E204, E208, and E209). GLM-based differential expression analyses then identified cell clusters within tumors, cell types within clusters, and contrasts between CNCCs or TNCCs and other cell types in pulmonary LAM tumors.

Bioinformatics Analysis

Ingenuity Pathway Analysis (IPA) software (v68752261, Qiagen) was used to perform a Core Analysis (6) on genetic markers identified from CLC Genomics Workbench data in *Tsc2*$^{+/-}$ mouse renal tumors and WT tissue. A right-tailed Fisher's exact test ($p < 0.05$) assessed the significance of the association between genetic markers and gene networks, functions, and canonical pathways curated in IPA. P-values were adjusted with the Benjamini-Hochberg method to account for multiple testing. Z-scores measured the similarity between gene expression patterns (up- or downregulated) and the activation ($Z \geq 2.0$) or inhibition ($Z \leq -2.0$) of canonical pathways. The ratio of genetic markers assigned to each pathway or function ($Q < 0.05$; $-1.0 \leq \text{Log2FC} \leq 1.0$) to the total genes in that pathway/function was also calculated for each analysis.

A regulator effect analysis was performed on differentially expressed genes (DEGs) identified between CNCCs and other cell types in *Tsc2*$^{+/-}$ mouse renal tumors, with a bias toward neurocristopathic targets. This analysis identified upstream regulator networks associated with these targets and predicted their downstream effects. It also identified potential therapeutic targets and formulated testable hypotheses regarding signaling that govern neurocristopathic tumorigenesis in *Tsc2*$^{+/-}$ mice. Resolved upstream regulator-downstream function pairs for neurocristopathic tumor targets were ranked by consistency scores, which measure the causal match between upstream regulator networks and the expression changes they drive in the CNCC dataset that cause the downstream effect (tumorigenesis). Ranked regulator-downstream function pairs exhibited significant Fisher's Exact test p-values ($p < 0.05$) that measured overlap between upstream regulator and disease/function dataset molecules. The results of the regulator effect analysis of DEGs in CNCCs are tabulated in Supplemental Table 8.

Immunohistochemistry

De-identified human renal cortex, LAM renal angiomyolipoma, and healthy lung tissue sections (4 µm thick) were obtained from the Molecular Pathology Core Facility at Columbia University Medical Center (CUMC) and processed as described. FFPE sections of de-identified LAM patient pulmonary tumors, derived from fresh biopsies at NDRI, were also prepared and processed similarly. Additionally, fibrotic lung tissue sections from a 57-year-old patient with idiopathic pulmonary fibrosis, enrolled at Columbia's Center for LAM and Rare Lung Disease (New York City), were prepared with consent and stained accordingly. All mouse tissues were prepared in-house; mice were euthanized via slow inhalation overdose of isoflurane (>4.5%, approximately 2 minutes), followed by tissue and tumor excision.

Paraffin Tissue Immunohistochemistry

Excised tissues and tumors were briefly rinsed in PBS, fixed overnight in 10% buffered formalin, then processed and embedded in paraffin. Sections (4 μm) were stained with H&E. For IHC, paraffin sections were autoclaved at 121°C for 20 min in Tris-HCl buffer (10 mmol/L, pH 9.0) for antigen retrieval and incubated with 6% hydrogen peroxide in methanol for 20 min to block endogenous peroxidase before staining.

Frozen Tissue Immunohistochemistry

Excised tissues were briefly rinsed in PBS, fixed overnight in 4% PFA at 4°C, then sequentially soaked in 15% and 30% sucrose for 24 hours each (7). They were embedded in OCT, snap-frozen in isopentane on dry ice, and sectioned at 4μm thickness at Columbia University's Molecular Pathology Core.

Immunofluorescence staining of paraffin-embedded and frozen tissue sections was performed using primary and secondary antibodies listed in Supplemental Table 9. Nuclear staining was performed for 30 min with Hoechst 33342 (10μg/ml; Cat# 62249, Thermo Fisher Scientific). Autofluorescence was quenched for 5 min (Cat# SP-8400, Vector Laboratories), and the slides were then mounted with antifade medium (Cat# H-1900, Vector Laboratories) and imaged by confocal microscopy. Control sections (positive and negative) were processed for comparison and compiled in Supplemental Figures 14 and 15. Antibody control sections are displayed alongside each respective marker.

Image Acquisition

All stained histology slides were imaged using a Nikon A1 confocal microscope on a TiE Eclipse inverted microscope with a Plan Apo VC 20x/0.75 NA objective lens (Nikon Instruments Inc.). The pinhole was set to 1 Airy Unit for Alexa Fluor 647. Excitation and emission settings were: Hoechst (405 nm, 425-75 nm), Alexa Fluor 488 (488 nm, 500-550 nm), Alexa Fluor 647 (640 nm, 655-800 nm). Z-stacks with 1.5 μm steps were captured, and maximum-intensity projections were generated. Images were acquired under identical settings, with 5-7 images per tissue section in random order. Fast, wide-area imaging was performed with a Nikon AXR MP resonant scanner on a Ti2 microscope equipped with a motorized XY stage, with a pixel size of 0.908 μm. Tiled z-stacks covered entire tissue sections for detailed spatial mapping of neurocristopathic markers in both LAM and normal tissues. All systems were operated with NIS-Elements AR software version 5.0.2.01 or 6.

Western blot

Tissues and tumors were homogenized in 1x RIPA buffer with protease and phosphatase inhibitors (Cat# 78442; Thermo Fisher Scientific). 30 μg of protein was separated on 10% SDS-polyacrylamide gels, transferred to nitrocellulose membranes, and probed with primary antibodies (Supplemental Table 10). Antibody binding was detected using peroxidase-

labeled anti-rabbit (1:5000; Cat# ab205718) or anti-mouse (1:5000; Cat# ab205719) secondary antibodies (Abcam), with visualization via ECL (Cat#34580, Thermo Fisher Scientific) on an iBright FL1500 system (Cat# A44241, Invitrogen-Thermo Fisher Scientific). Where applicable, protein bands were quantified by normalizing to the housekeeping protein (β-actin or Cyclophilin B) and expressed relative to the marker protein in WT mouse kidney tissue. Relative protein content ratios in the immunoblots were determined using iBright Analysis software (Thermo Fisher Scientific) and analyzed using a one-way ANOVA with multiple comparisons and post-hoc Dunnett's test, comparing WT mouse kidney tissues to *Tsc2*$^{+/-}$ mice renal tumors.

RT-qPCR

Total RNA was extracted from mouse renal tissues, tumors, hepatic tissues, lesions, healthy human lung tissue, and LAM patient pulmonary neoplasms using a RNeasy Mini Kit (Qiagen) according to the manufacturer's protocol. RNA quality was assessed by DNase treatment and UV spectroscopy. Reverse transcription was performed with a High-Capacity cDNA Reverse Transcription Kit (Cat# 4368813; Applied Biosystems) using 1µg of total RNA. Gene expression was quantified by qPCR in 20 µL reactions, run in quadruplicate with TaqMan probes on a QuantStudio 3 system (Cat# A28567, Applied Biosystems). Data was analyzed using QuantStudio software to obtain ΔΔCt values. Pre-designed Taq-man-based assays for neural crest markers (*LCN2, CLU, SOX9, SPP1, TFAP2A, KRT18*) and canonical LAM markers (*ACTA2, PMEL, MLANA, CTSK, TSC2*) are listed in Supplemental Table 11. Validation was performed using tissues from 3 to 8 biological samples.

Neural Crest Cell Culture

The 09-1 mouse CNCC line (Cat# SCC049, Millipore Sigma) was used for *Spp1* gene recombination experiments. These NCCs were derived from tissue above the second pharyngeal arch in E8.5 *Wnt1*-Cre; R26R-GFP mouse embryos and sorted by flow cytometry based on GFP expression (8). NCCs are multipotent, capable of indefinite passaging, and can differentiate into osteoblasts, chondrocytes, smooth muscle, and glial cells (9). They robustly express canonical neural crest markers, including *Tfap2a*, *Twist1*, *Sox9*, and *Ets1* (8). Cultured in a 5% CO_2 incubator at 37°C, NCCs were plated on Matrigel-coated flasks (250µg/ml; Cat# E1270) with complete mouse embryonic stem cell media, supplemented with leukemia inhibitory factor (LiF; 10^3 U/ml) and 15% FBS (Cat# ES-101-B), and 25 ng/ml basic fibroblast growth factor (bFGF; Cat# GF003) (all from Millipore Sigma), added before use (8, 9). Cells were passaged at 80% confluency to prevent differentiation, and the media were changed every 3 days. Passages 2-5 were used for gene recombination.

For serum starvation experiments, NCC media was prepared with modifications to established protocols (8, 9) using conditioned media from an overnight (16-hour) incubation of embryonic fibroblast STO feeder cells (Cat# CRL-1503; ATCC)

at 5% CO_2 and 37°C in high glucose DMEM (Cat# 11995-040), supplemented with 0.1 mM minimal essential media (Cat# 11140-050), 55μM β-mercaptoethanol (Cat# 21985), all from Gibco, Thermo Fisher Scientific, 1% Penn-Strep, and 0.5% FBS. The conditioned medium was then supplemented with 25 ng/ml bFGF and 1000 U/ml mouse LiF (Cat# ESG1106; Millipore Sigma), sterile-filtered (0.22 μm), and used as serum-starved media for NCCs.

Generation of *Spp1*-knockout Neural Crest Cell Lines

Synthego designed and synthesized Cas9 nuclease and multi-guide RNAs (sgRNAs) (Supplemental Table 12) to target exon 4 of *Spp1* in the 09-1 NCC line. Ribonucleoproteins (RNPs) were prepared by incubating sgRNAs with Cas9 for 10 minutes at room temperature. These RNPs were transfected into NCCs (sgRNA: Cas9 ratio 5:1 v/v) using nucleofection with the P3 Primary Cell 4D-Nucleofector X Kit on a Lonza 4D-Nucleofector X system (Program DS150; Cat# AAF-1003X, Lonza). A mouse ROSA26 sgRNA (Supplemental Table 12) served as a positive control to optimize transfection parameters, including sgRNA:Cas9 ratios and cell concentrations, with transfection efficiency and nucleofector conditions determined using the pMAXGFP vector (1 μg/μl; Lonza) as a control. Negative controls omitted sgRNA and/or Cas9. The sgRNA sequences are listed in Supplemental Table 12. Electroporated NCCs were cultured to generate single-cell colonies, and after 72 hours, PCR was used to identify edits at the *Spp1* locus via Sanger sequencing (Genewiz) and Inference of CRISPR Edits (ICE) analysis (Synthego). PCR primers used to identify edits are listed in Supplemental Table 10. Loss of *Spp1* expression in mutant NCCs was confirmed by RT-qPCR and Western blot.

Generation of *Tsc2*-knockout Neural Crest Cell Lines

CRISPR/Cas9-mediated gene recombination at the *Tsc2* locus in O9-1 mouse CNCCs was achieved using a Cas9 D10 enzyme fused to GFP, three sgRNAs (Supplemental Table 12) (AA1, AB1, AC1) targeting exon 3 (>NC_000083.7:24849887-24849973), 8 (>NC_000083.7:24845220-24845345), and 6 (>NC_000083.7:24846965-24847082) of *Tsc2*, respectively, and an electroporation enhancer, all from Integrated DNA Technology (IDT). RNP complexes were formed by incubating sgRNAs with Cas9 in a 2:1 ratio for 20 minutes at room temperature. Transfection was performed via nucleofection using the P3 Primary Cell 4D-Nucleofector with program DS150, following mixing with an electroporation enhancer. Transfection efficiency of the nucleofector program conditions was determined using pMAXGFP as a control. Negative controls lacked sgRNA or Cas9.

Forty-eight hours after nucleofection, PCR products from AA1-, AB1-, and AC1-transfected NCCs were sequenced to identify NCC colonies with edits at the *Tsc2* gene locus using ICE analysis. The primers used are listed in Supplemental Table 12. GFP-conjugated Cas9 enabled visualization of enzyme delivery into NCCs post-transfection via microscopy,

confirming the presence of mutant *Tsc2*-ko NCCs in the pre-sorted population. Based on ICE results, AC1 mutant *Tsc2*-ko NCCs were selected, and single-cell clones were generated by flow sorting GFP^+, viable NCCs into Matrigel-coated 96-well plates. Loss of *Tsc2* expression in the*se* clones was validated by RT-qPCR and western blot.

Transwell Migration & Invasion Assays

The invasion and migration rates of *Spp1*-ko and *Tsc2*-ko mouse CNCCs versus WT CNCCs were evaluated using Matrigel-coated (2 mg/ml) and non-ECM-coated transwells, respectively, as previously described (10). NCCs were seeded in the upper chambers of 8 µm pore-size transwells (Cat# 5666-2638; USA Scientific) in serum-starved medium for 24 hours at 37°C in 5% CO_2. Cells that migrated to the lower chamber containing complete medium with 15% FBS were fixed in ice-cold methanol for 10 min, then stained with Hoechst 33342 (10µg/ml) for 30 min. Migration was assessed at low (1×10^3) and high (5×10^3) cell densities in triplicate or quadruplicate transwells per genotype, with three repetitions. Hoechst-positive cells were counted in 5 fields of view (diameter = 2.2 mm; area = 0.038 cm^2) per transwell using an inverted fluorescence microscope at 100X (Nikon). Counts were scaled to the 0.33 cm^2 area of the transwells, and percentages of migrated or invasive cells were calculated by dividing by the initial seeded cell number.

BrdU Proliferation Assay

The effect of inhibiting *Spp1* and *Tsc2* expression on CNCC proliferation was assessed by measuring BrdU incorporation (Cat# 6813, Cell Signaling Technology), a thymidine analog, as previously described. NCCs (2,500 per well) were cultured in Matrigel-coated (250 µg/ml) 96-well plates in quadruplicate with complete NCC media for 24 hours at 37°C in 5% CO_2. Cell proliferation rates were then measured according to the manufacturer's instructions, and the experiments were repeated on three separate days.

Cell Viability Assay

The impact of inhibiting *Spp*1 and *Tsc2* expression on CNCC viability was assessed by measuring ATP levels in WT and *Spp1*-ko NCCs using a luminescence assay (Cell Titer-Glo; Cat# G7571, Promega). The assay was performed three times with 2,500 NCCs per well in quadruplicate on opaque 96-well plates, following the manufacturer's protocol. To generate a standard curve, NCCs ranging from 0 to 10,000 cells were used, showing ATP concentrations directly proportional to viable cell numbers. This allowed comparison of viability percentages among *Tsc2*-KO*, Spp1*-KO, and non-edited NCCs after overnight incubation in complete media.

Enzyme-Linked Immunosorbent Assay (ELISA)

Fresh blood samples (10 ml) from healthy volunteers (N=3; age 35-70) and age-matched female LAM patients (N=12; age 35-70) cared for at the Center for LAM and Rare Lung Disease, CUMC, were centrifuged at 3000 × g for 5 minutes to obtain serum. Serum levels were measured using ELISA kits according to manufacturer protocols with duplicate assays for each of the following markers: SPP1 (Cat# BMS2066), KRT18 (Cat# EH145RB), CLU (Cat# EHCLU), LCN2 (Cat# EHLCN2) (all from Thermo Fisher Scientific), and VEGF-D (Cat# ab233625, Abcam).

Statistics

Images from migration and invasion assays were processed, and cell counts were automated using Fiji (Version 2.14.0). Cell count, proliferation, and viability data for *Spp1*-KO versus *Spp1*-WT NCCs were analyzed with unpaired two-tailed Student's t-tests, and $p<0.05$ was considered significant (*). Serum protein levels and normalized gene expression in healthy volunteers versus LAM patients were compared using a one-way ANOVA with Dunnett's post-hoc test. Differences in NCC marker expression related to mouse tissue or tumor age were assessed with paired Student's t-tests. Statistical analyses and graphs were generated with GraphPad Prism (Version 10.3.1). All error bars in figures represent mean ± SEM.

Data Availability

The raw datasets and analysis files generated from RNA sequencing of single-cell dissociates from 1) WT and *Tsc2*$^{+/-}$ mouse renal tumors and tissues, and 2) human LAM pulmonary neoplasms, are publicly available in the NCBI Gene Expression Omnibus under GSE291150 and GSE292058, respectively. The single-cell RNA data used to train neural crest cell signatures were obtained from GSE129114 (4), with the analyzed data available at https://pklab.med.harvard.edu/ruslan/neural.crest.html. All graph data points are detailed in the Supporting Data Values file.

SUPPLEMENTAL TABLES

Supplemental Table 1: Top 100 CNCC genes in E9.5 Wnt1-Cre; R26R mice embryos is presented separately as an excel file

Supplemental Table 2: Differentially expressed genes (DEGs) (n=824 genes) in CNCCs in renal cystadenomas of the tuberous sclerosis *Tsc2*$^{+/-}$ mice is presented separately as an excel file

Supplemental Table 3: Description of NDRI LAM patient cohort

LAM Sample ID	Age	Race	Gender	LAM Diagnosis	Time Since Diagnosis[A] (Years)	AML	Single Cell Count (n)
E202	45	Caucasian	Female	Yes	15	Yes	4972
E204	61	Caucasian	Female	Yes	14	Yes	3570
E208	60	Caucasian	Female	Yes	12	Yes	4091
E209	60	Caucasian	Female	Yes	25	Yes	4918

NDRI = National Disease Research Interchange. LAM = Lymphangioleiomyomatosis. AML = Renal angiomyolipoma.
[A] Calculated from the time of LAM diagnosis till sample collection.

Supplemental Table 4: High confidence predictions of cell types in clusters 1-7 in healthy lung and LAM pulmonary neoplasms

Cluster #	Major cell types (High confidence)	
	LAM pulmonary neoplasm	Healthy lung parenchyma
1H (Healthy), 1HH, 1T (Tumor)	Alveolar type 1 cells, epithelial cells, endothelial cells, cranial neural crest cells, airway basal cells, enterocytes	Alveolar type 1 cells, alveolar type 2 cells, cranial neural crest cells, ciliated epithelial cells.
2	Endothelial cells, Skeletal muscle satellite cells	Epithelial cells, epithelial precursor cells, fibroblasts, alveolar type 1 cells, ciliated epithelial cells
3	Endothelial cells, sinusoidal endothelial cells	Endothelial cells, sinusoidal endothelial cells
4	Ciliated epithelial cells, epithelial cells, cranial neural crest cells	Ciliated epithelial cells, epithelial cells, cranial neural crest cells
5	M2 macrophages, macrophages, T Lymphocytes, monocytes, plasma cells	M2 macrophages, macrophages, T Lymphocytes, monocytes, plasma cells
6	Fibroblasts, stromal cells, trunk neural crest cells	Fibroblasts, stromal cells, trunk neural crest cells
7	Smooth muscle cells, skeletal muscle satellite cells, pericytes, endothelial cells.	Smooth muscle cells, skeletal muscle satellite cells, pericytes, endothelial cells.

Cell types in pulmonary neoplasms from lymphangioleimyomatosis (LAM) patients (N=4 patients, N=17551 cells) and in healthy lung tissues (N=4 donors, N=27093 cells) were identified by correlating representative gene signatures in the cells with the weighted maximum observed gene expressions per cell type curated in the Qiagen CLC Genomics Workbench used for scRNAseq analysis. The CLC Qiagen reference data manager compiles gene expression data from 474 biological samples across 148 different human cell types. Probability testing is performed using Platt scaling, and cell types are predicted with ‘high confidence’ when probabilities exceed 50%. Cell types with similar gene signatures are grouped into the same cluster.

Supplemental Table 5: Top 100 TNCC genes in E9.5 Sox10CreERT2; R26Rtomato+ embryos is presented separately as an excel file.

Supplemental Table 6: Differentially expressed genes (DEGs) (n= 90 genes) in CNCCs compared to other cell types in cluster #1T of LAM pulmonary neoplasms is presented separately as an excel file

Supplemental Table 7: Differentially expressed genes (DEGs) (n= 472 genes) in TNCCs compared to other cell types in cluster #6 of LAM pulmonary neoplasms is presented separately as an excel file.

Supplemental Table 8: Regulator effect networks is presented separately as an excel file.

Supplemental Table 9 – Antibody resource table for Immunohistochemistry

Antibody	Source	Dilution	Catalogue# / Identifier
Mouse monoclonal anti-osteopontin	Abcam	1:200	Cat# ab166709<br>RRID:AB_2936514
Rabbit monoclonal anti-transcription factor AP-2α	Abcam	1:200	Cat# ab108311<br>RRID:AB_10861200
Mouse monoclonal anti-Sox9	Abcam	1:300	Cat# ab76997<br>RRID:AB_2194156
Rabbit monoclonal anti-cytokeratin 18	Abcam	1:800	Cat# ab181597<br>RRID:AB_2922417
Rabbit monoclonal anti-melanoma gp100	Abcam	1:200	Cat# ab137078<br>RRID:AB_2732921
Rabbit polyclonal anti-phospho-p70 S6 Kinase (Thr389)	Abcam	1:100	Cat# ab2571<br>RRID:AB_303169
Rabbit polyclonal anti-alpha smooth muscle actin	Abcam	1:100	Cat# ab5694<br>RRID:AB_2223021
Rabbit monoclonal anti-lipocalin-2	Abcam	1:200	Cat# ab125075<br>RRID:AB_10978084
Donkey anti-rabbit IgG Alexa Fluor 488<br>(Highly cross adsorbed)	Thermo Fisher Scientific	10 µg/mL	Cat# A21206<br>RRID:AB_2535792
Donkey anti-rabbit IgG Alexa Fluor 647<br>(Highly cross adsorbed)	Thermo Fisher Scientific	1:1000	Cat# A31573<br>RRID:AB_2536183
Donkey anti-Mouse IgG (H+L) Highly Cross-Adsorbed Secondary Antibody, Alexa Fluor™ 647	Thermo Fisher Scientific	10 µg/mL	Cat# A31571<br>RRID: AB_162542
Donkey anti-Mouse IgG (H+L) Highly Cross-Adsorbed Secondary Antibody, Alexa Fluor™ 488	Thermo Fisher Scientific	10 µg/mL	Cat# A21202<br>RRID: AB_141607

Supplemental Table 10 – Antibody resource table for western blot

Antibody	Source	Dilution	Catalogue# / Identifier
Rabbit monoclonal anti-osteopontin	Cell Signaling Technologies	1:1000	Cat# 88742<br>RRID:AB_3107209
Mouse monoclonal anti-Sox9	Abcam	1:1000	Cat# ab76997<br>RRID:AB_2194156
Rabbit monoclonal anti-cytokeratin 18	Abcam	1:2000	Cat# ab181597<br>RRID:AB_2922417
Rabbit monoclonal anti-tuberin	Abcam	1:1000	Cat# ab255612<br>RRID: AB_3073945
Mouse monoclonal β-actin antibody	Abcam	1:5000	Cat# ab6276<br>RRID:AB_2223210
Rabbit monoclonal anti-cyclophilin B	Abcam	1:5000	Cat# ab178397<br>RRID:AB_2924975
Mouse monoclonal anti-glyceraldehyde phosphate dehydrogenase	Abcam	1:1000	Cat# ab8245<br>RRID:AB_2107448
Goat anti-rabbit IgG (H&L)<br>(Horse radish peroxidase (HRP)-conjugated)	Abcam	1:5000	Cat# ab205718,<br>RRID:AB_2819160
Goat anti-mouse IgG (H&L)<br>(Horse radish peroxidase (HRP)-conjugated)	Abcam	1:5000	Cat# ab205719,<br>RRID:AB_2755049

Supplemental Table 11 – Oligonucleotide resource table for pre-designed TaqMan assays

Gene Target	Source	Catalogue#	Assay
Lipocalin (*Lcn2*)	Thermo Fisher Scientific	4331182	Mm01324470_m1
Clusterin (*Clu*)	Thermo Fisher Scientific	4331182	Mm00442773_m1
SRY-box transcription factor 9 (*Sox9*)	Thermo Fisher Scientific	4331182	Mm00448840_m1
Secreted phosphoprotein 1 (*Spp1*)	Thermo Fisher Scientific	4331182	Mm00436767_m1
Transcription factor AP-2-alpha (*Tfap2a*)	Thermo Fisher Scientific	4331182	Mm00495574_m1
Cytokeratin 18 (*Krt18*)	Thermo Fisher Scientific	4331182	Mm01601704_g1
Eukaryotic 18s rRNA endogenous control (FAM/MGB probe – non-primer limited)	Thermo Fisher Scientific	4352930E	Hs99999901_s1
smooth muscle actin alpha 2 *(ACTA2)*	Thermo Fisher Scientific	4331182	Hs00426835_g1
Pre-melanosome protein (*PMEL*)	Thermo Fisher Scientific	4331182	Hs00173854_m1
Melanoma antigen recognized by T cells (*MLANA*)	Thermo Fisher Scientific	4331182	Hs00194133_m1
Cathepsin K *(CTSK)*	Thermo Fisher Scientific	4331182	Hs00166156_m1
Tuberin (*TSC2*)	Thermo Fisher Scientific	4331182	Hs01020387_m1

Supplemental Table 12 – Oligonucleotide resource table for gene recombination studies

Oligonucleotide Resource	Source	Catalogue#	Primer (5' > 3')
Spp1- Gene Knockout kit v2 - multi-guide gRNA	Synthego	GKO-MM1-000-0-003n-0-0	gRNA1: UGGCUAUAGGAUCUGGGUGC, gRNA2: AACACAAUACCUGUGGCGCA, gRNA3: UGGCAGAGCUGUUCUUACAG
Rosa26 – positive control	Synthego	052-1020-000-000-001n-Rosa26-0	ACUCCAGUCUUUCUAGAAGA
Tsc2- single guide sgRNA – AA1	Integrated DNA Technology	Mm.Cas9.TSC2.1.AA	ACAAUCGCAUCCGAAUGAUAG
Tsc2- single guide sgRNA – AB1	Integrated DNA Technology	Mm.Cas9.TSC2.1.AB	UGACAUUGAUGGUGCGGCACG
Tsc2- single guide sgRNA – AC1	Integrated DNA Technology	Mm.Cas9.TSC2.1.AC	UGACGAAUACAUUGCAUCAAG
Spp1 – Forward Primer	Thermo Fisher Scientific	10336022	AGACCAGCAGCATTGGCATA
Spp1 – Reverse Primer	Thermo Fisher Scientific	10336022	CCTAGCCTGTGTGGCTTGAT
Spp1 – Sequencing Primer	Thermo Fisher Scientific	10336022	AGTGTTCCATGTATCTGCCG
Tsc2 – Forward / Sequencing Primer	Thermo Fisher Scientific	10336022	TAAGCTTGAGACCCAAGACCG
Tsc2 – Reverse Primer	Thermo Fisher Scientific	10336022	GCAGTGATAGGATGCAGACC
Rosa26 – Forward / Sequencing Primer	Thermo Fisher Scientific	10336022	GAGGCGGATCACAAGCAATA
Rosa26 – Reverse Primer	Thermo Fisher Scientific	10336022	GGGAGGGGAGTGTTGCAATA

Bibliography

1. Unachukwu U, Sonett J, Woode D, Shiomi T, Chada K, and D'Armiento JM. Tyrosine Kinase Inhibitors Diminish Renal Neoplasms in a Tuberous Sclerosis Model Via Induction of Apoptosis. *Mol Cancer Ther.* 2023;22(7):844-58.
2. Quatromoni JG, Singhal S, Bhojnagarwala P, Hancock WW, Albelda SM, and Eruslanov E. An optimized disaggregation method for human lung tumors that preserves the phenotype and function of the immune cells. *J Leukoc Biol.* 2015;97(1):201-9.
3. Platt J. Probabilistic Outputs for Support Vector Machines and Comparisons to Regularized Likelihood Methods. *Adv Large Margin Classif.* 2000;10.
4. Soldatov R, Kaucka M, Kastriti ME, Petersen J, Chontorotzea T, Englmaier L, et al. Spatiotemporal structure of cell fate decisions in murine neural crest. *Science.* 2019;364(6444):eaas9536.
5. Ku AT, Wilkinson S, and Sowalsky AG. Comparison of approaches to transcriptomic analysis in multi-sampled tumors. *Brief Bioinform.* 2021;22(6).
6. Kramer A, Green J, Pollard J, Jr., and Tugendreich S. Causal analysis approaches in Ingenuity Pathway Analysis. *Bioinformatics.* 2014;30(4):523-30.
7. Unachukwu U, Shiomi T, Goldklang M, Chada K, and D'Armiento J. Renal neoplasms in tuberous sclerosis mice are neurocristopathies. *iScience.* 2021;24(7):102684.
8. Ishii M, Arias AC, Liu L, Chen YB, Bronner ME, and Maxson RE. A stable cranial neural crest cell line from mouse. *Stem Cells Dev.* 2012;21(17):3069-80.
9. Nguyen BH, Ishii M, Maxson RE, and Wang J. Culturing and Manipulation of O9-1 Neural Crest Cells. *J Vis Exp.* 2018(140).
10. Justus CR, Leffler N, Ruiz-Echevarria M, and Yang LV. In vitro cell migration and invasion assays. *J Vis Exp.* 2014(88):51046.

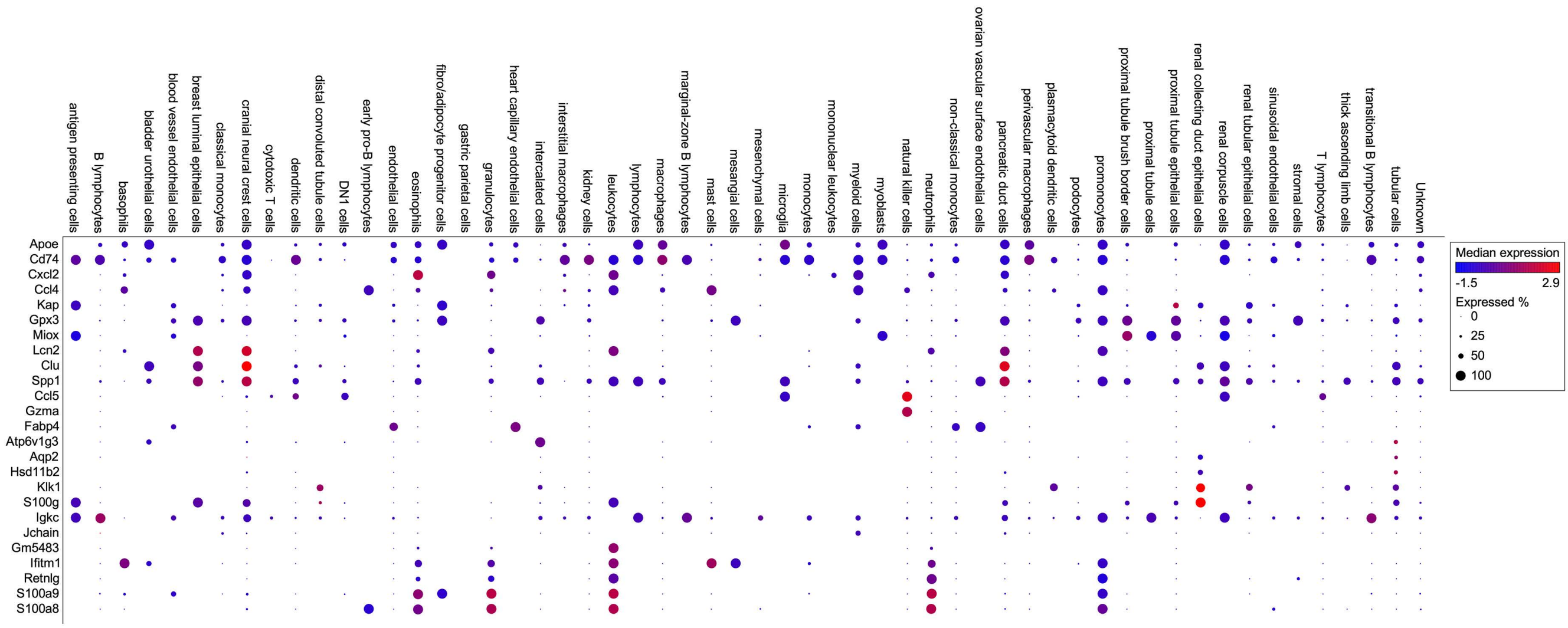

Supplemental Figure 1: Cell type annotation in tuberous sclerosis (*Tsc2*$^{+/-}$) mouse renal cystadenomas – Dot plot illustrating all 55 predicted cell types in *Tsc2*$^{+/-}$ mouse renal tumors. An excerpt is provided in **figure 1D**. It shows the median expression levels of 25 genes with the highest variability, comparing single-cell RNA sequencing data from wild-type C57BL6/J mouse renal tissues (N = 3 mice; 19078 cells) to Tsc2+/- mouse renal tumors (N = 4 mice; 26861 cells). Cell type clustering was performed using a Wilcoxon rank-sum test for differential expression within the Single-cell RNA-Seq analysis workflow (CLC Genomics Workbench Version 23.0.4). Circle sizes correspond to the percentage of cells expressing each gene, while red and blue hues represent up- and down-regulated gene expression, respectively.

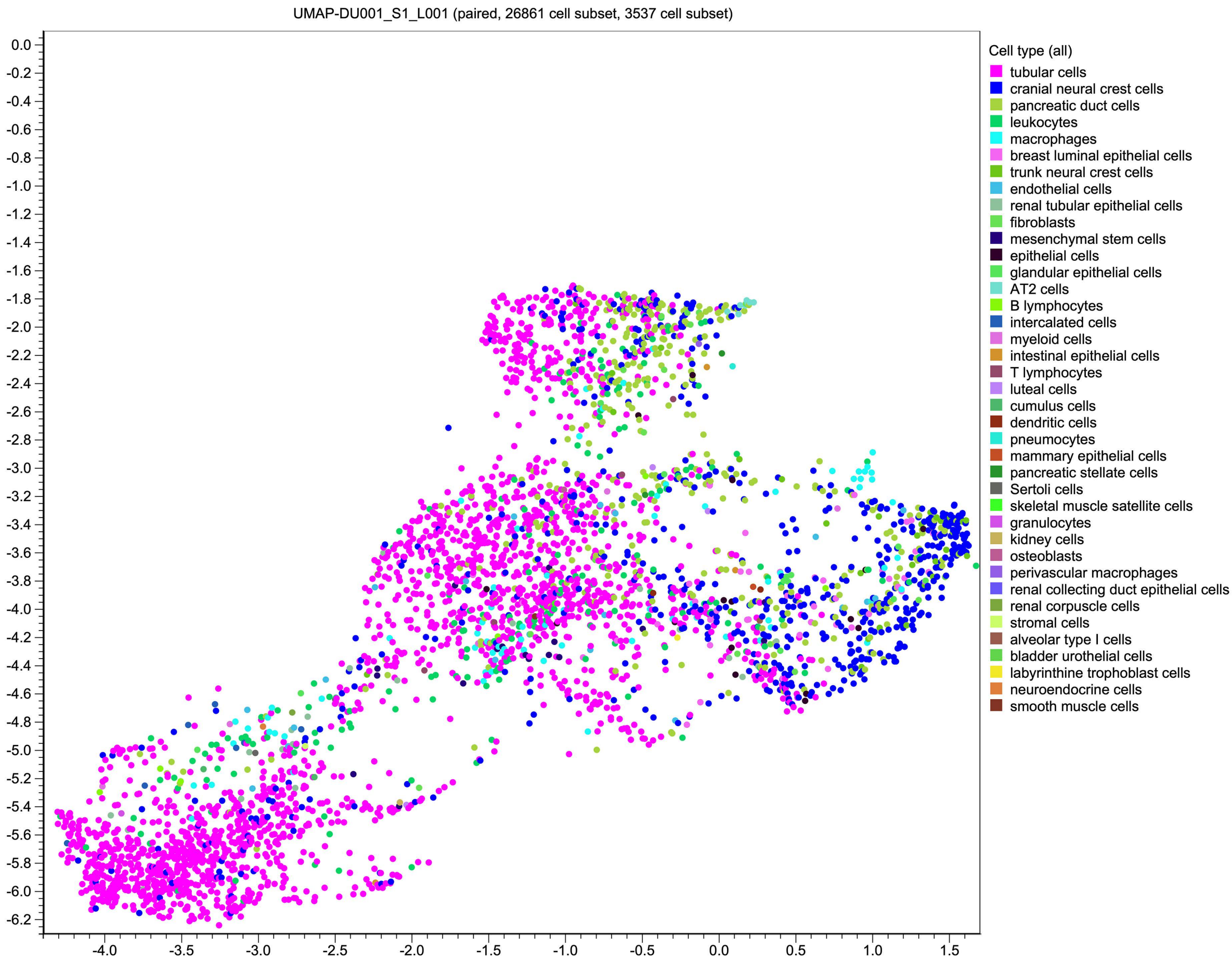

Supplemental Figure 2: Classification of cell types in cluster 8 of tuberous sclerosis (*Tsc2*$^{+/-}$) mouse renal tumors – UMAP clustering of single cells in cluster 8 (N = 3537 cells) of *Tsc2*$^{+/-}$ mouse renal cystadenomas (N = 4 mice; 26861 cells). Cell type classification was based on log-transformed values of the maximum observed gene expression per cell and on the relative sizes of the various gene classes used to define different cell types. However, this broad “Cell Type -All” classification (N=39 cell types), though Platt-probability-scaled, does not account for thresholds when defining cell types.

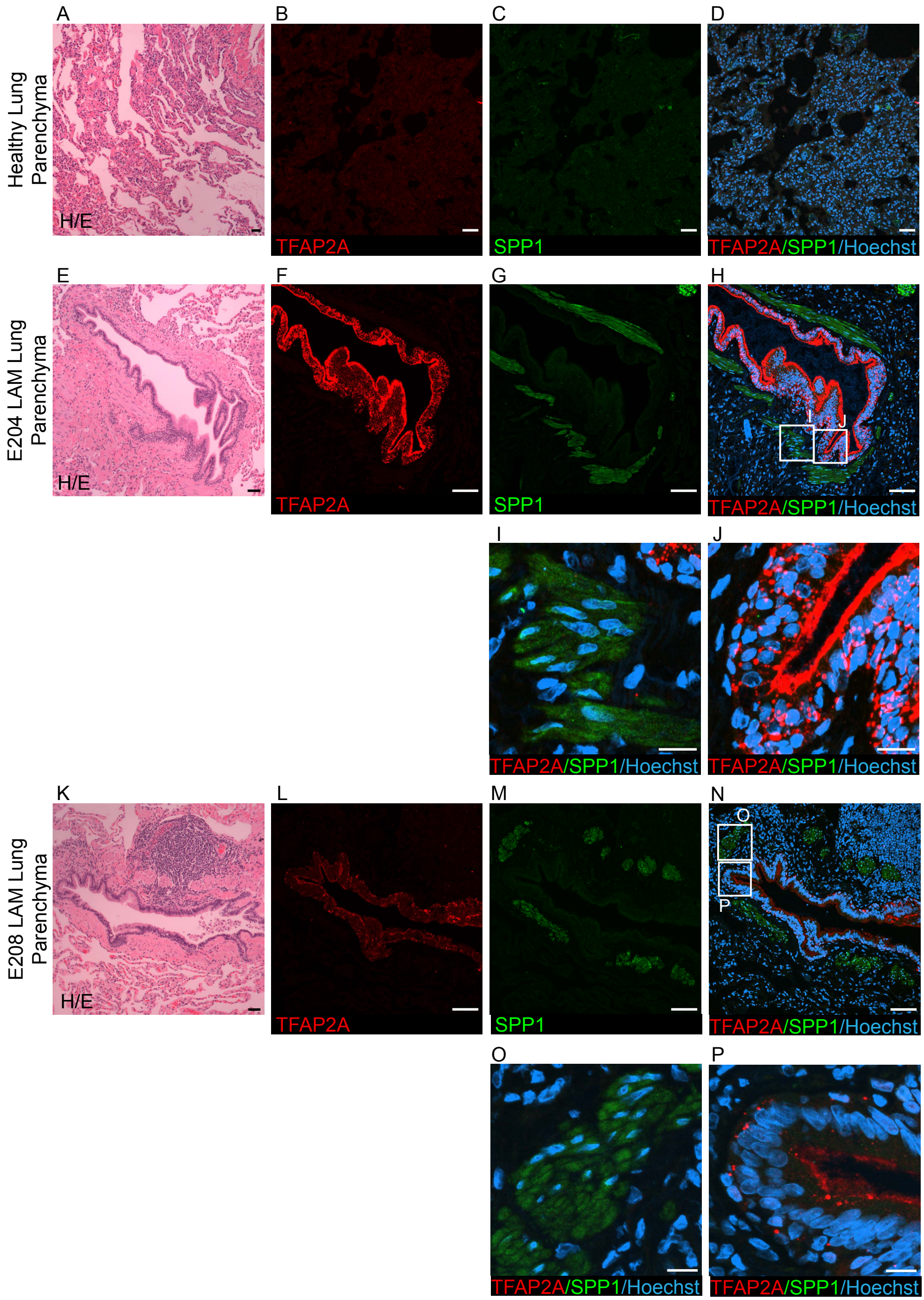

Supplemental Figure 3: Evidence of neurocristopathy in Lymphangioleiomyomatosis (LAM) pulmonary neoplasms (SPP1 & TFAP2A) – **(A-D)** Co-localization IHC using FFPE tissue slides demosntrating the absence of osteopontin (SPP1) and early neural crest marker transcription factor activating protein 2 (TFAP2A) in healthy human lung parenchyma. TFAP2A is localized in the apical membrane and cilia of airway epithelial cells, with moderate cytoplasmic expression observed in pulmonary neoplasms from LAM patient E204 **(F, H, J)** and E208 **(L, N, P)**. SPP1 expression extends beyond the airway smooth muscle into the parenchyma of pulmonary neoplasms from the same patients as shown in panels **(G)**, **(H)**, and **(I)** (E204) and **(M)**, **(N)**, and **(O)** (E208). Boxed inserts **(I)** and **(J)** in **Figure H** and **(O)** and **(P)** in **Figure N** are magnified, respectively. Hoechst 33342 was a nuclear counterstain across all slides. Scale bar: 20µm (magnified images), 50µm (all other panels). Images were captured using a Nikon TiE Eclipse confocal microscope.

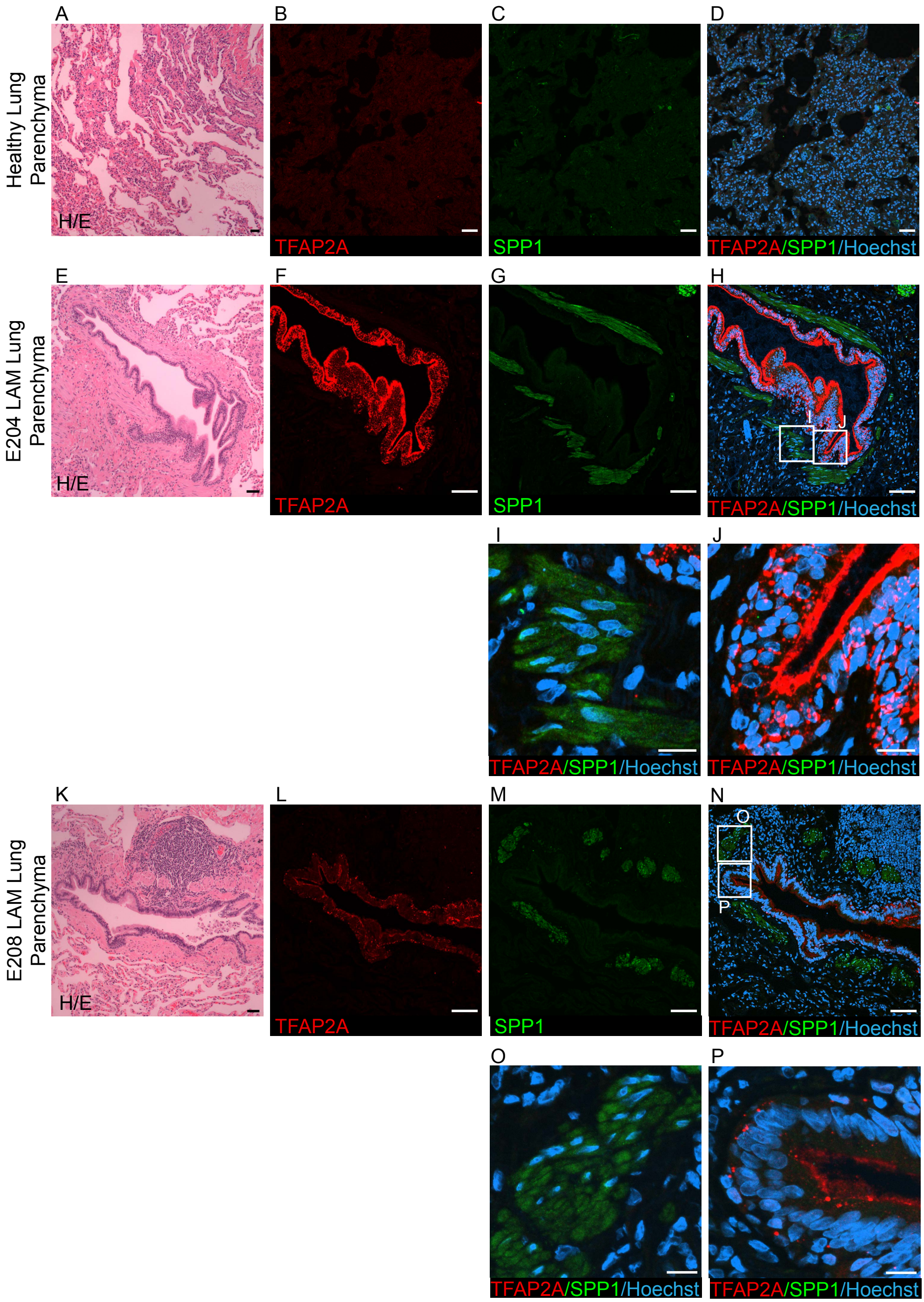

Supplemental Figure 3: Evidence of neurocristopathy in Lymphangioleiomyomatosis (LAM) pulmonary neoplasms (SPP1 & TFAP2A) – **(A-D)** Co-localization IHC using FFPE tissue slides demosntrating the absence of osteopontin (SPP1) and early neural crest marker transcription factor activating protein 2 (TFAP2A) in healthy human lung parenchyma. TFAP2A is localized in the apical membrane and cilia of airway epithelial cells, with moderate cytoplasmic expression observed in pulmonary neoplasms from LAM patient E204 **(F, H, J)** and E208 **(L, N, P)**. SPP1 expression extends beyond the airway smooth muscle into the parenchyma of pulmonary neoplasms from the same patients as shown in panels **(G)**, **(H)**, and **(I)** (E204) and **(M)**, **(N)**, and **(O)** (E208). Boxed inserts **(I)** and **(J)** in **Figure H** and **(O)** and **(P)** in **Figure N** are magnified, respectively. Hoechst 33342 was a nuclear counterstain across all slides. Scale bar: 20µm (magnified images), 50µm (all other panels). Images were captured using a Nikon TiE Eclipse confocal microscope.

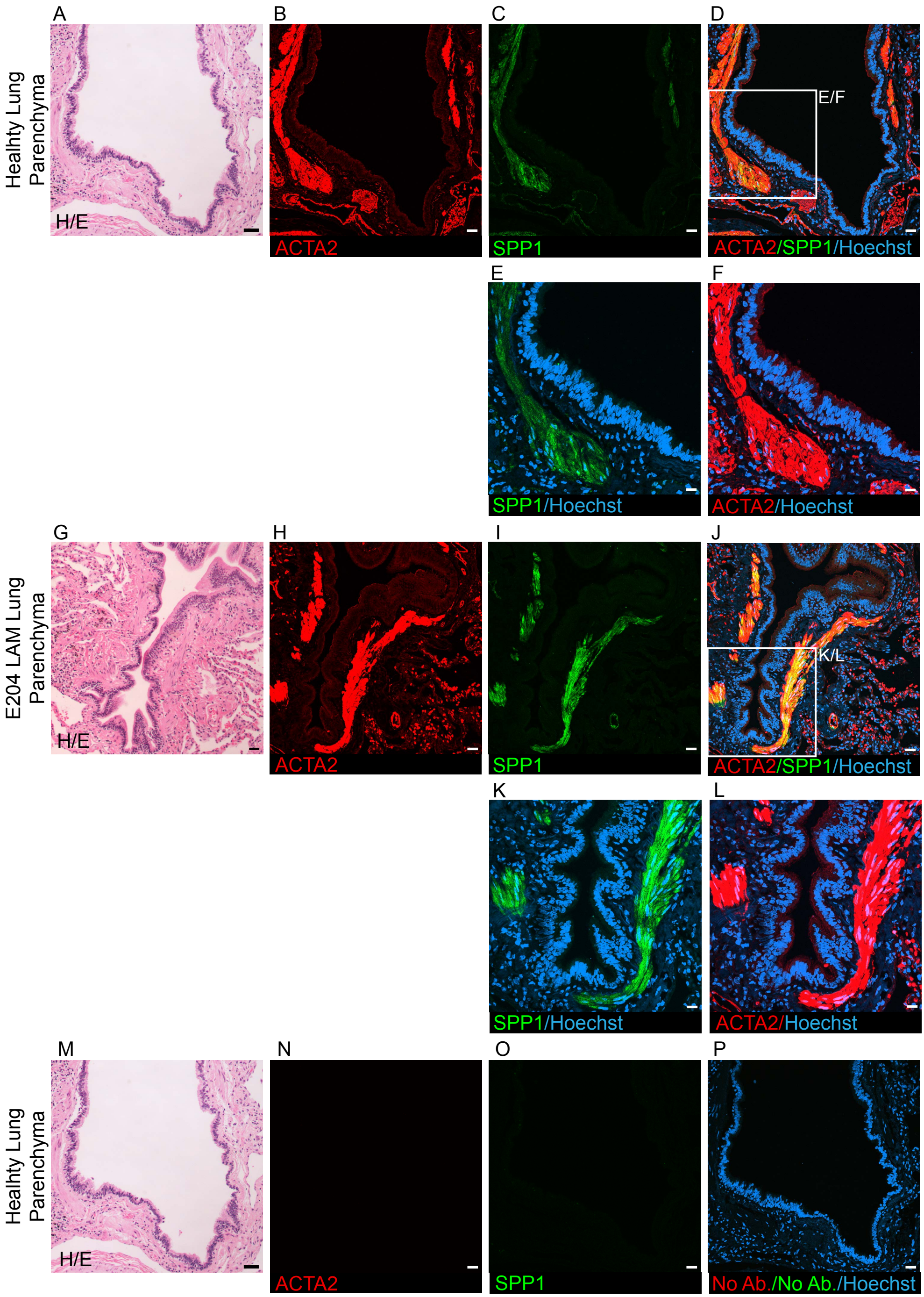

Supplemental Figure 4: Evidence of neurocristopathy in Lymphangioleiomyomatosis (LAM) pulmonary neoplasms (SPP1 & ACTA2) – **(A-F)** Co-localization IHC using FFPE tissue slides reveals overlapping moderate expression of alpha smooth muscle actin (ACTA2), a LAM marker, and low levels of osteopontin (SPP1), a neurocristopathic marker. Boxed inserts **(E)** and **(F)** in **Figure D** are enlarged, highlighting SPP1 and ACTA2 co-localization within bronchiolar airway smooth muscle. **G-L)** ACTA2 and SPP1 co-localize beyond the bronchiolar airway smooth muscle into the parenchyma of pulmonary neoplasms of LAM patient E204. Boxed inserts **(K)** and **(L)** in **Figure J** are magnified. **M-P)** Healthy lung tissue sections processed without primary antibody (antibody controls). Positive and negative control staining for SPP1 and ACTA2 are provided in **Supplemental Figures 14** and **15** respectively. Hoechst 33342 was used as a nuclear counterstain in all images. Scale bar: 20µm (magnified images), 50µm (all other panels). Images were captured using a Nikon TiE Eclipse confocal microscope.

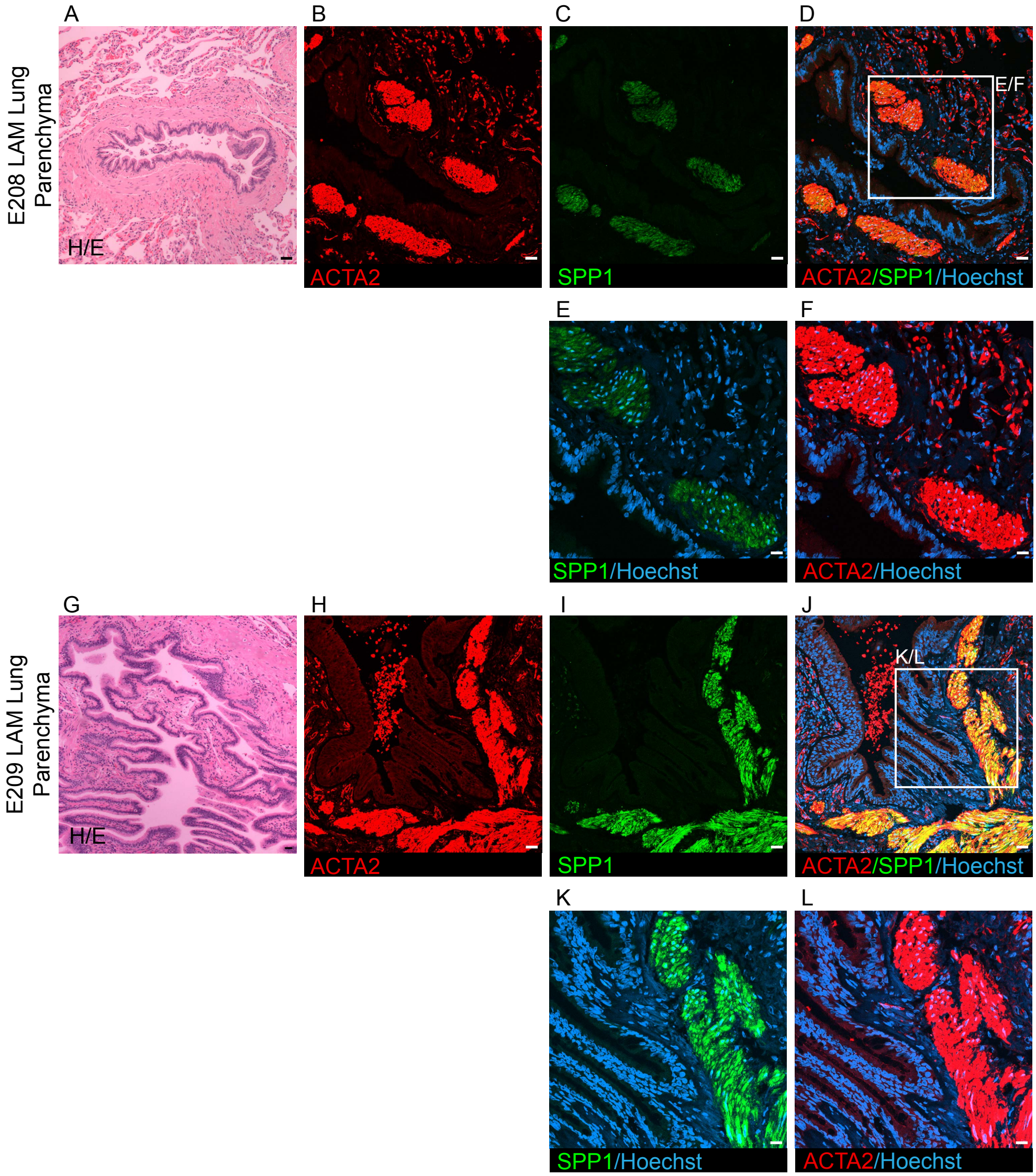

Supplemental Figure 5: Evidence of neurocristopathy in Lymphangioleiomyomatosis (LAM) pulmonary neoplasms (SPP1 & ACTA2) contd. – LAM marker alpha smooth muscle actin (ACTA2) and neurocristopathic marker osteopontin (SPP1) co-localize beyond the smooth muscle region of bronchiolar airways, extending into the lung parenchyma in LAM patient E208 **(A-F)** and E209 **(G-L)**. This pattern contrasts with observations in healthy lungs **(Supplemental Figure 4)**. Enlarged views highlighting overlapping SPP1 and ACTA2 expression are presented in boxed regions **(E)** and **(F)** in **Figure D**, and **(K)** and **(L)** in **Figure J**. Positive, negative, and antibody controls for SPP1 can be visualized in **Supplemental Figure 14**, and in **Supplemental Figures 4** and **15** for ACTA2, respectively. All slides were counterstained with Hoechst 33342 to label nuclei. Scale bars: 20µm (magnified images), 50µm (all other panels). Images were captured using a Nikon TiE Eclipse confocal microscope.

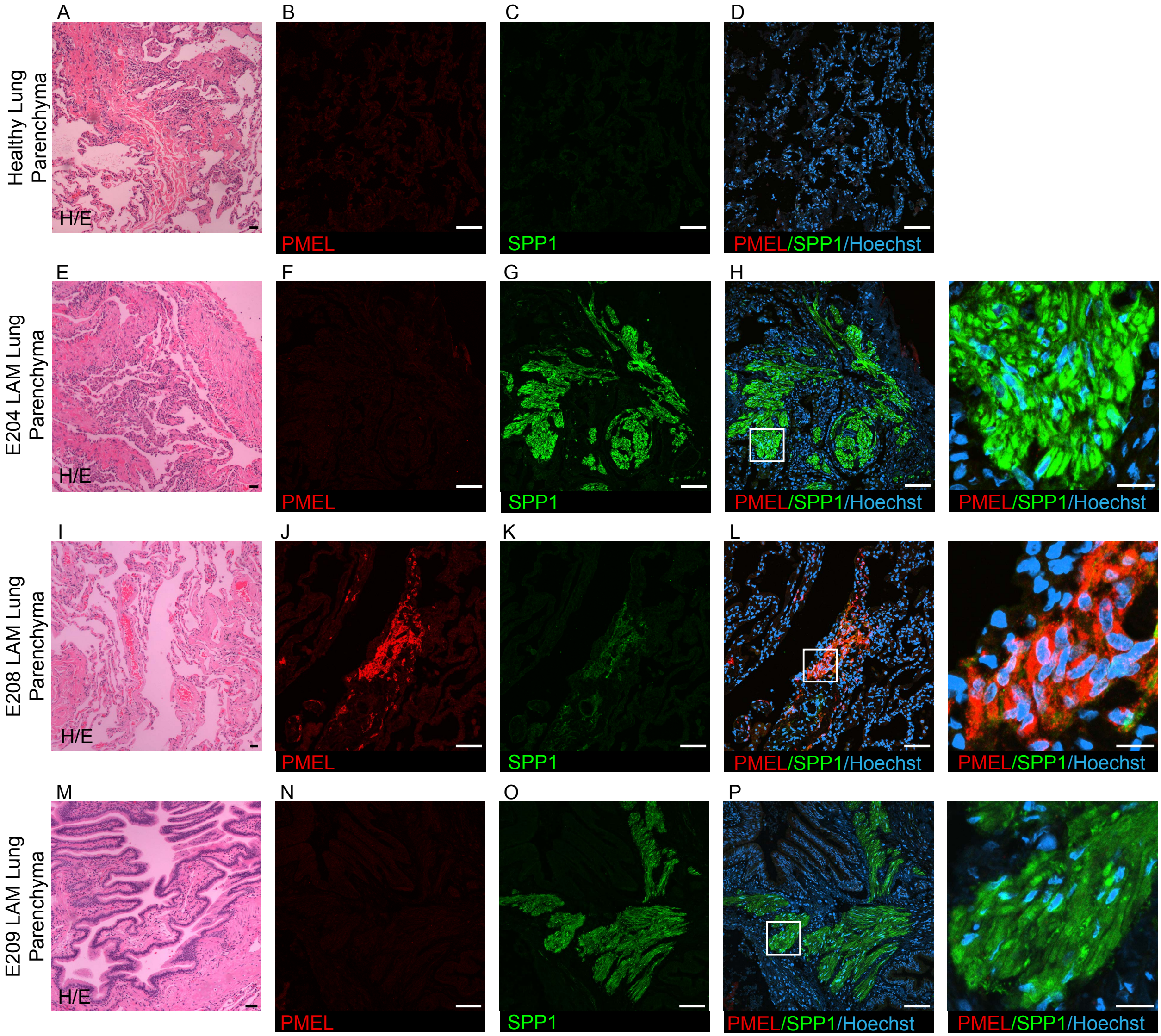

Supplemental Figure 6: Evidence of neurocristopathy in Lymphangioleiomyomatosis (LAM) pulmonary neoplasms (SPP1 & PMEL) – H/E staining **(A)** and co-localization IHC of the pre-melanosome protein, PMEL, and the neurocristopathic marker, osteopontin (SPP1) in healthy human lung parenchyma **(B-D)**. PMEL and SPP1 co-localize within the parenchyma of lung tumors from LAM patient E208 **(I-L)**, but not in neoplasms from patients E204 **(E-H)** or E209 **(M-P)**. Notably, SPP1 is consistently overexpressed in all pulmonary neoplasms examined. Boxed inserts are magnified in the adjacent panels. Hoechst 33342 is used as a nuclear counterstain in all slides. Scale bar: 20µm (magnified images), 50µm (all other panels). Images were captured using a Nikon TiE Eclipse confocal microscope.

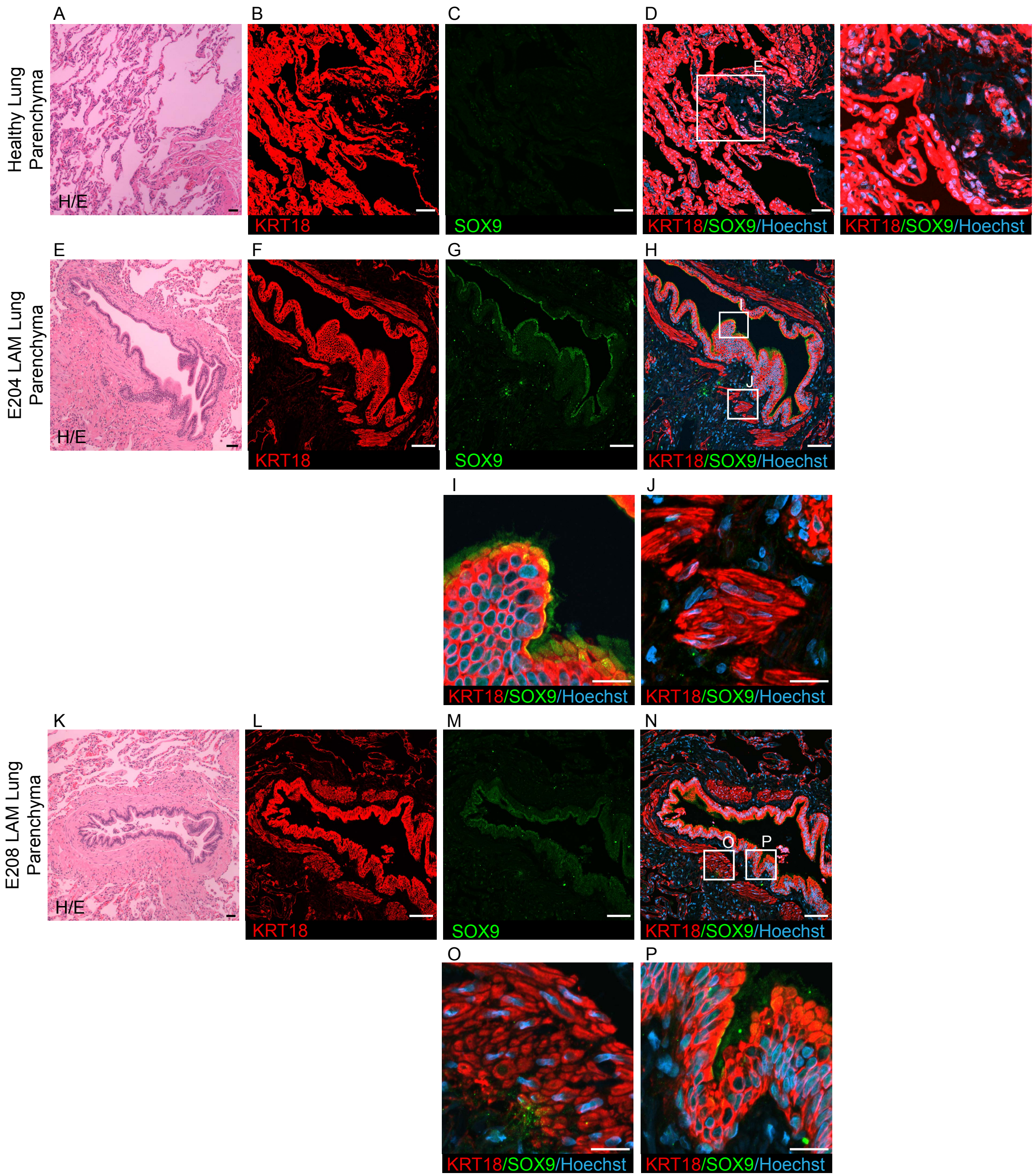

Supplemental Figure 7: **Evidence of neurocristopathy in Lymphangioleiomyomatosis (LAM) pulmonary neoplasms (KRT18 & SOX9)** – H/E staining **(A)** and co-localization IHC of cytokeratin 18 (KRT18) **(B, D, E)** and SRY-box transcription factor 9 (SOX9) **(C, D, E)** in FFPE tissue sections of healthy lung parenchyma. The boxed region in **Figure D** is magnified in the adjacent panel. KRT18 expression is observed both in the cytoplasm of pseudostratified columnar airway epithelia cells and in the adjacent smooth muscle tissue in LAM patient E204 **(F, H-J)** and E208 **(L, N-P)**. SOX9 expression is detected in the apical membrane and cilia of patient E204 **(G-J)** but less prominently in patient E208's LAM lungs **(M-P)**. Boxed regions **(I)** and **(J)** in **Figure H**, and **(O)** and **(P)** in **Figure N**, are magnified. Positive and negative control tissues for KRT18 and SOX9 staining are shown in **Supplemental Figure 15**. Hoechst 33342 serves as a nuclear counterstain across all slides. Scale bar: 20μm (magnified images), 50μm (all other panels). Images were acquired using a Nikon TiE Eclipse confocal microscope.

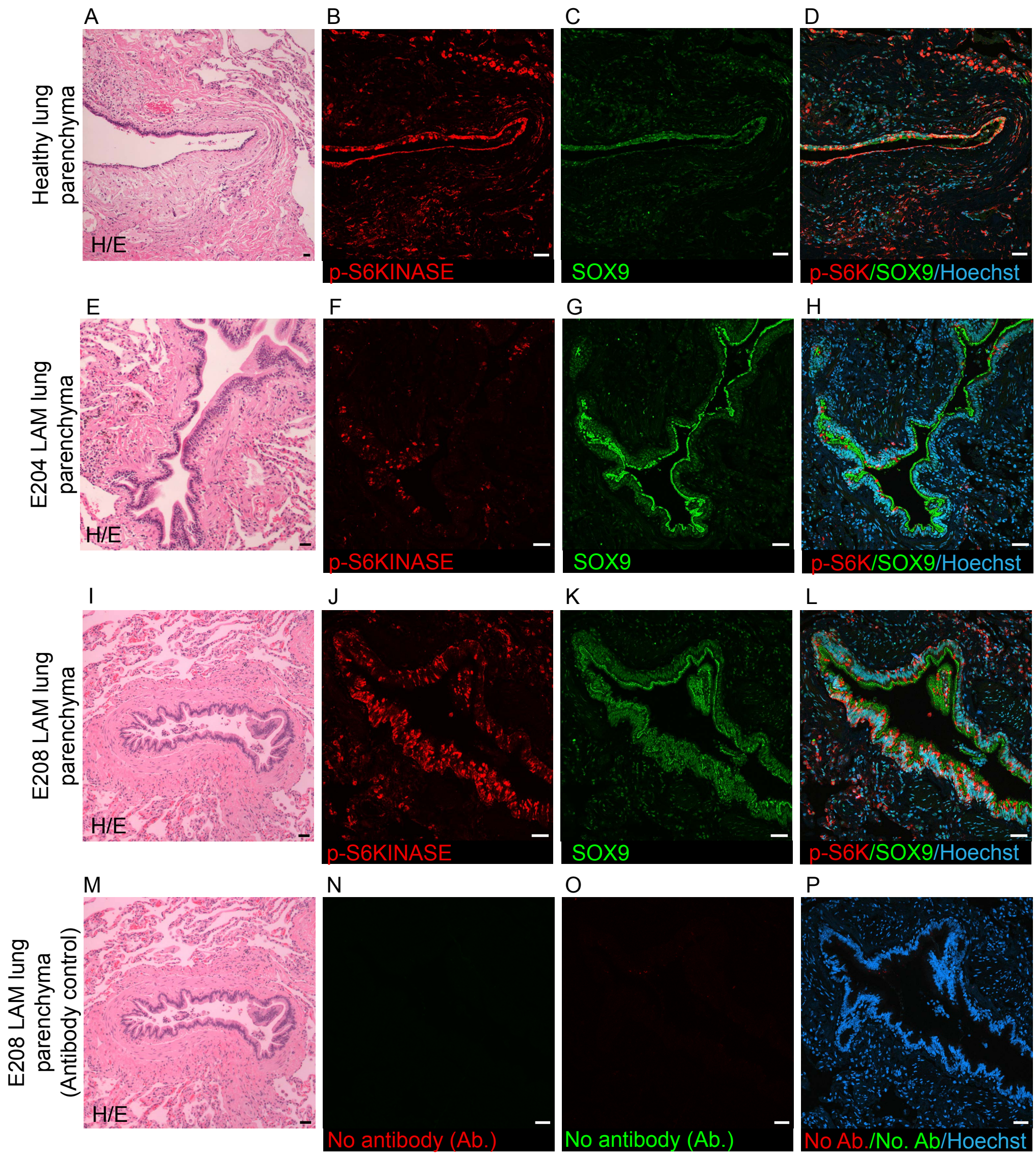

Supplemental Figure 8: Evidence of neurocristopathy in Lymphangioleiomyomatosis (LAM) pulmonary neoplasms (p-S6K & SOX9) – A-D) H/E staining and co-localization IHC of SRY-box transcription factor 9 (SOX9) and phosphorylated p70S6 kinase (p-S6K) in FFPE tissue from healthy lung parenchyma. Double immunolabeling indicates minimal p-S6K expression and SOX9 localization to the apical membrane in patient E204 LAM lungs **(E-H)**, whereas widespread pathologic p-S6K expression is observed in E208 LAM lungs **(I-L)**. **M-P)** Patient E208 pulmonary neoplasm processed without primary antibody (antibody control). Positive and negative control tissues for SOX9 are provided in **Supplemental Figure 15**. Hoechst 33342 is used as a nuclear counterstain across all slides. Scale bar: 50µm. Images were captured using a Nikon TiE Eclipse confocal microscope.

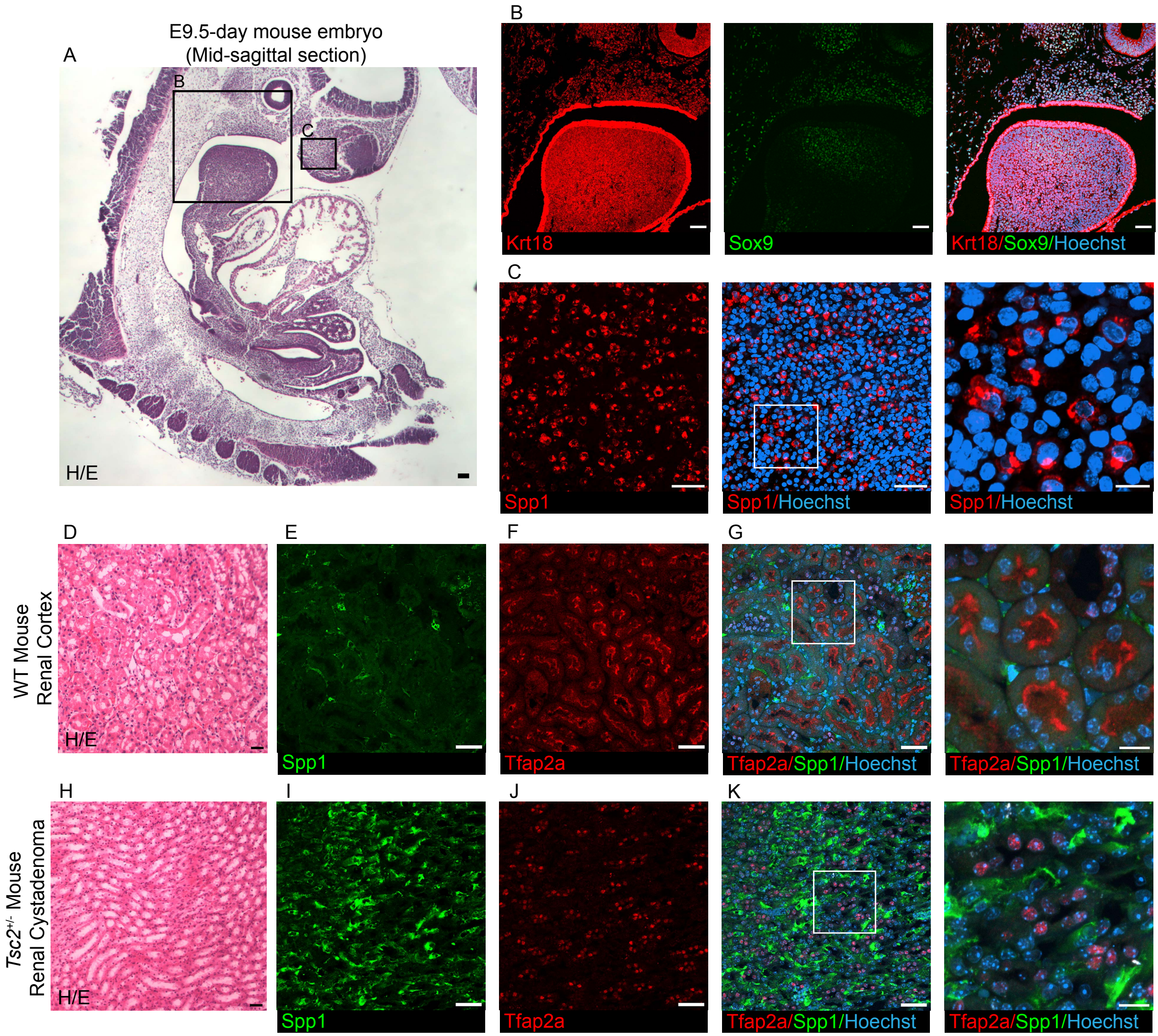

Supplemental Figure 9: Evidence of neurocristopathy in tuberous sclerosis (*Tsc2*$^{+/-}$) mouse renal cystadenoma — A) H&E-stained image of an embryonic E9.5-day C57BL/6J mouse, with boxed regions **(B)** and **(C)** magnified to depict IHC results for Krt18 and Sox9 **(B)**, and Spp1 **(C)** expression in the cranio-pharyngeal region. Co-immunolocalization of Spp1 and Tfap2a in adult WT mouse renal cortical tissue **(D-G)** and in *Tsc2*$^{+/-}$ mouse renal cystadenoma **(H-K)**. All boxed inserts are magnified in the respective adjacent panel. IHC results for antibody control slides (without primary antibody) are displayed in **Supplemental Figure 10**, while **Supplemental Figures 14** and **15** present comparison images for negative and positive control IHC slides similarly processed. Hoechst 33342 was used as a nuclear counterstain across all 4-µm-thick FFPE slides. Scale bar: 20 µm (magnified images) and 50 µm (all other panels). Images were captured using a Nikon Ti eclipse confocal microscope.

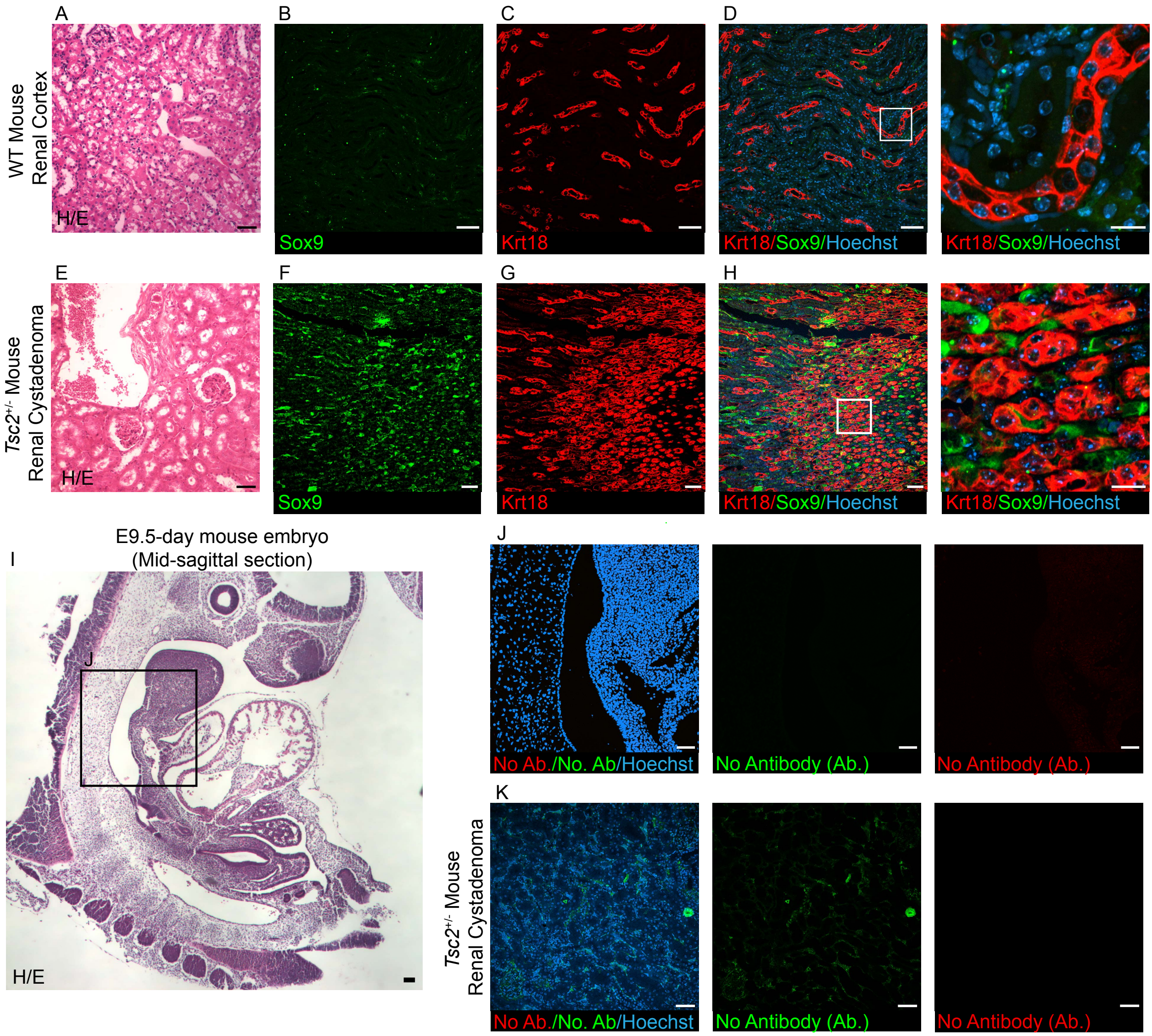

Supplemental Figure 10: Evidence of neurocristopathy in tuberous sclerosis (*Tsc2*$^{+/-}$) mouse renal cystadenomas (contd.) – H/E and double immunolabeling of Sox9 and Krt18 in adult C57BL/6J mouse renal cortex **(A-D)** and *Tsc2*$^{+/-}$ mouse renal tumor **(E-H)**. Boxed inserts are magnified in adjacent image panes. **I)** An H&E-stained image of embryonic E9.5-day C57BL/6J mice features a boxed insert exemplifying a region processed via IHC as antibody controls (no primary antibody) and magnified in **Figure J**. **K)** Double immunolabeled sections of antibody control tissue from tuberous sclerosis (*Tsc2*$^{+/}$) mouse renal cystadenomas used for comparison. **Supplemental figures 14** and **15** presents images of negative and positive control IHC slides prepared using the same protocols as above. Hoechst 33342 was used as a nuclear counterstain in all slides. Scale bar: 20 μm (magnified images) and 50 μm (all other panels). Images were captured with a Nikon Ti Eclipse confocal microscope.

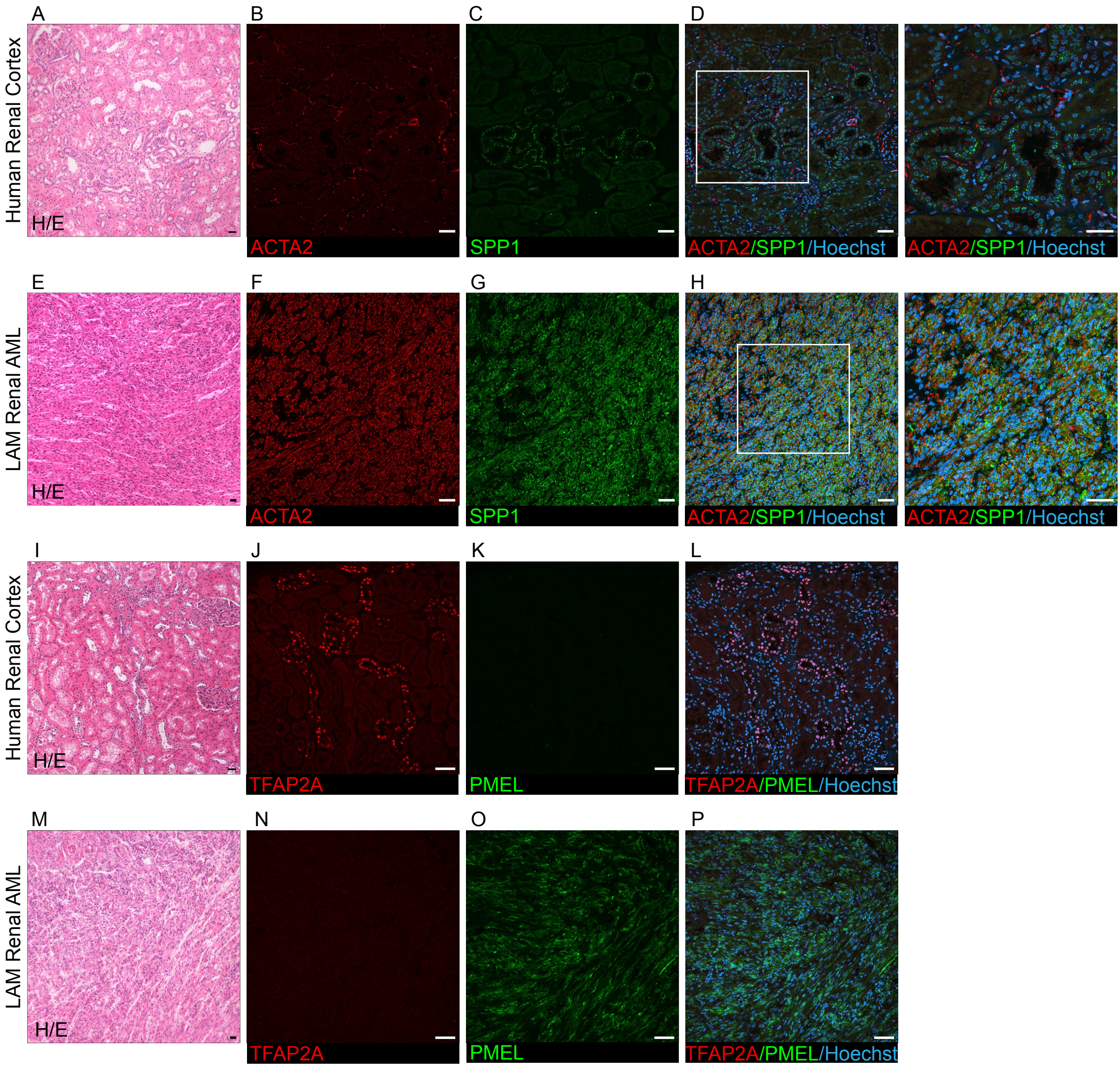

Supplemental Figure 11: Evidence of neurocristopathy in LAM renal angiomyolipoma (AML) — **A-D)** H&E staining and double-label IHC demonstrate absence of ACTA2 and minimal SPP1 expression restricted to collecting ducts in healthy renal cortex. **E-H)** In AML, widespread overexpression of ACTA2 and SPP1 in the renal cortex and medulla was observed. TFAP2A was expressed in the distal tubules of healthy renal cortex but not PMEL **(I-L)**, whereas in renal AML, PMEL was overexpressed with no TFAP2A detected **(M-P)**. All control tissue slides imaged for IHC comparisons can be visualized in **Supplemental figures 14** and **15**. Enlarged views of boxed inserts are provided in the respective adjacent panels. Hoechst 33342 served as a nuclear counterstain. Scale bar: 20µm (magnified images), 50µm (all other panels). Images were captured using a Nikon TiE Eclipse confocal microscope.

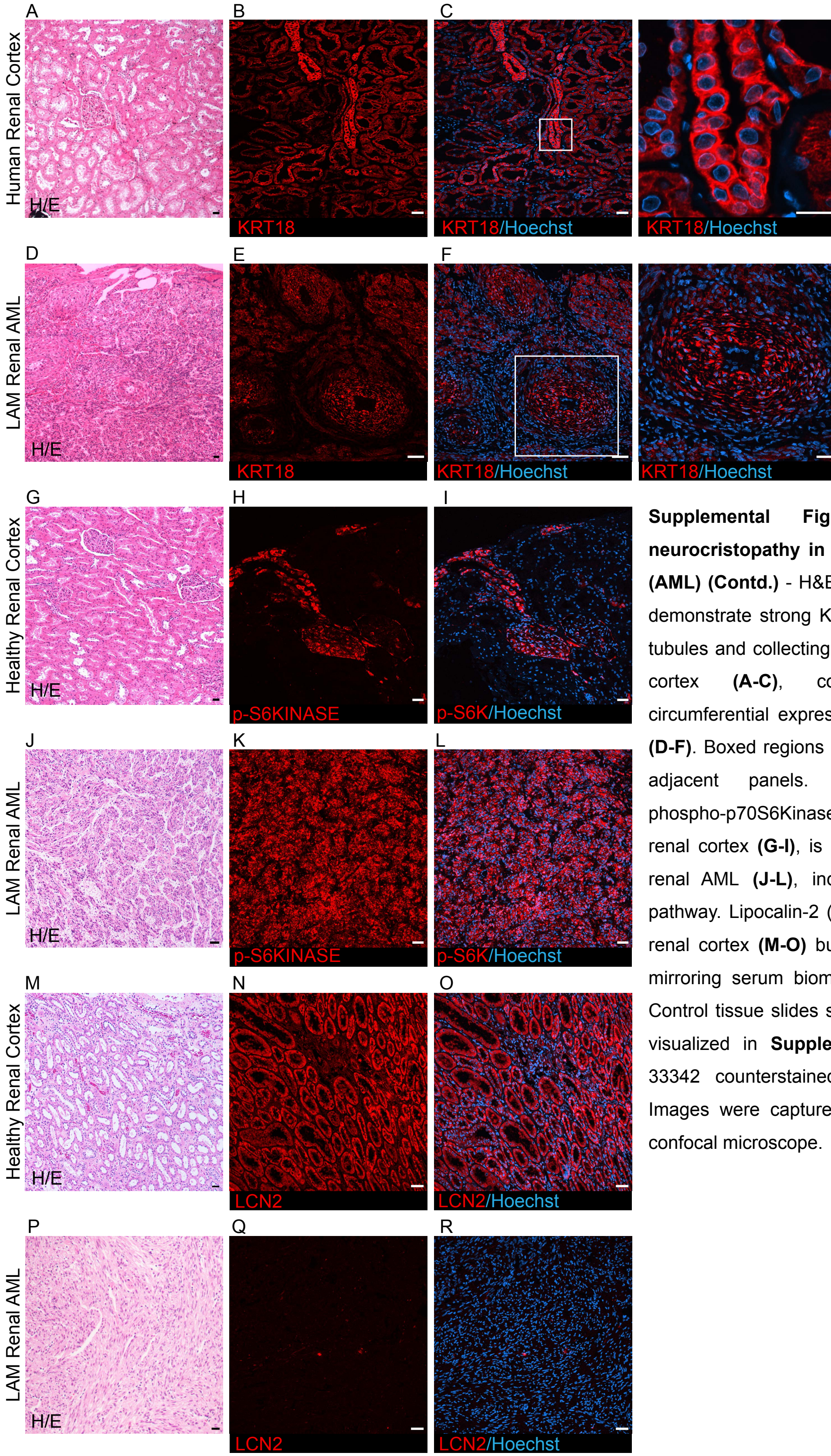

Supplemental Figure 12: Evidence of neurocristopathy in LAM renal angiomyolipoma (AML) (Contd.) - H&E staining and immunolabeling demonstrate strong KRT18 expression in the distal tubules and collecting ducts of human healthy renal cortex **(A-C)**, contrasted with moderate circumferential expression around LAM renal cysts **(D-F)**. Boxed regions are enlarged in the respective adjacent panels. Localized expression of phospho-p70S6Kinase (T389) (p-S6K) in healthy renal cortex **(G-I)**, is ubiquitously overexpressed in renal AML **(J-L)**, indicating an activated mTOR pathway. Lipocalin-2 (LCN2) is abundant in healthy renal cortex **(M-O)** but absent in renal AML **(P-R)**, mirroring serum biomarker patterns **(Figure 10C)**. Control tissue slides stained for comparison can be visualized in **Supplemental Figure 15**. Hoechst 33342 counterstained nuclei. Scale bar: 50μm. Images were captured using a Nikon TiE Eclipse confocal microscope.

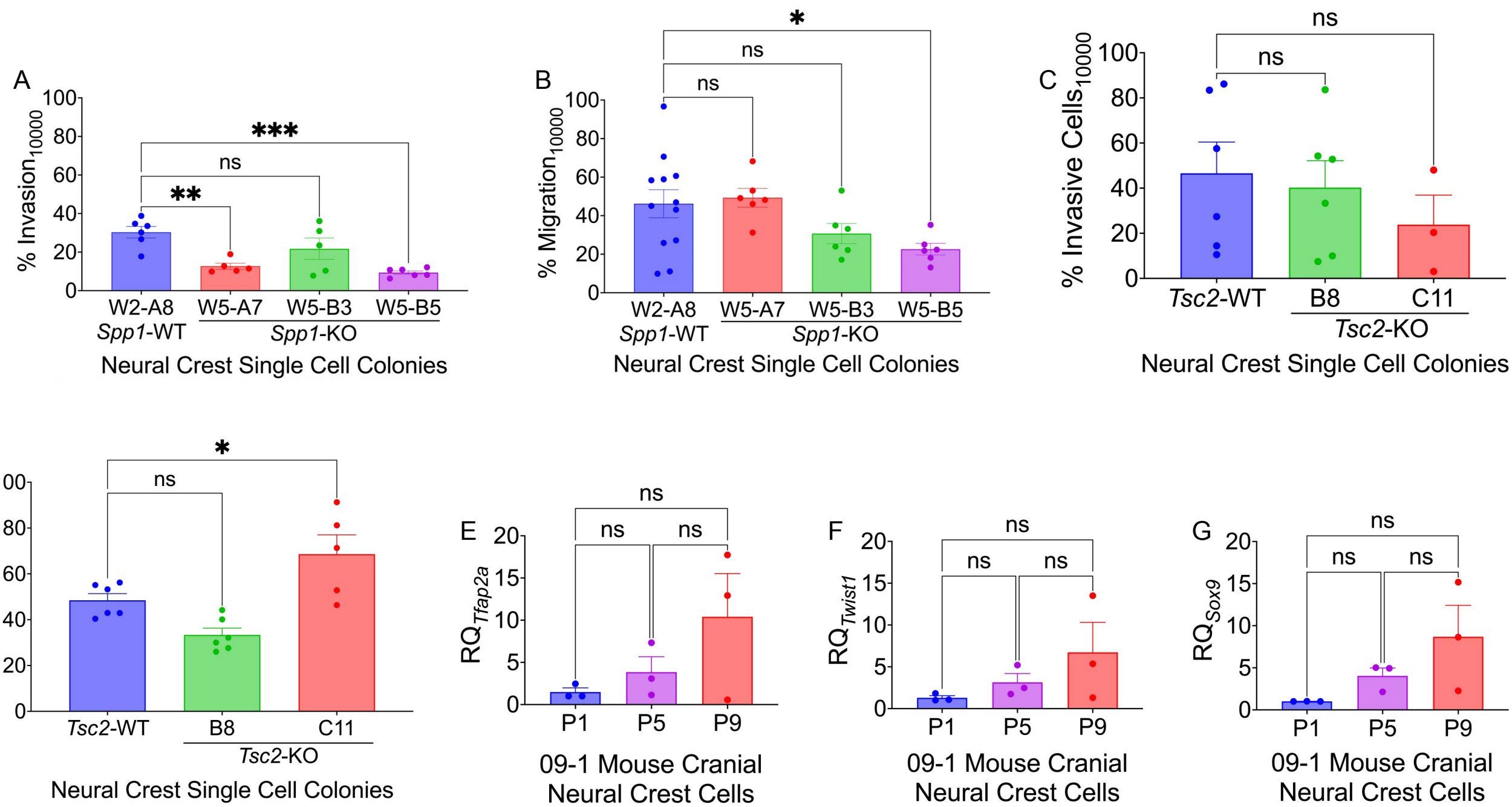

Supplemental Figure 13: Effects of gene recombination and routine maintenance on multipotency and chemotaxis of neural crest cells (NCCs): **A-B)** Mutant 09-1 mouse NCC clones (W5-A7, W5-B3, W5-B5) generated by CRISPR-mediated KO of exon 4 in the osteopontin (*Spp1*) gene were seeded at a density of 10,000 cells per well in 8µm transwells matrigel-coated (for invasion assays) or uncoated (for migration assays). After overnight incubation with chemoattractant (10% FBS) in the lower chamber, mutant NCCs exhibited significantly reduced invasion rates (W5-A7, W5-B5) **(A)** and migration rates (only W5-B5) **(B)** than the non-edited W2-A8 clone. **C)** Mutant NCC clones B8 and C11, harboring CRISPR edits at exon 6 of the *Tsc2* gene, showed no marked difference in invasion rates relative to non-edited clones when seeded at the same density (10,000 cells per well). **D)** The NCC-*Tsc2*-KO clone C11 demonstrated significantly higher migration rates than wild-type NCCs. Data from similar experiments using higher NCC densities (50,000 cells per well) are reported in **Figure 11**. The maintenance of multipotency during this study was assessed by measuring the expression of canonical neural crest markers in 09-1 mouse NCCs at different culture passages. Expression levels of transcription factor activation protein (*Tfap2a*) **(E)**, *Twist1* **(F)**, and SRY-Box transcription factor 9 (*Sox9*) **(G)** did not vary significantly across passages, although a slight increase was observed over time. Each data point represents the mean ± SEM of at least three independent experiments, with each experiment including triplicate or quadruplicate wells per biological sample. Statistical significance was evaluated using a one-way ANOVA with post-hoc Dunnett's or Šídák's multiple comparisons test. Asterisks denote significant differences: *p<0.05, **p<0.01, ***p<0.0005, ****p<0.0001; ns = not significant.

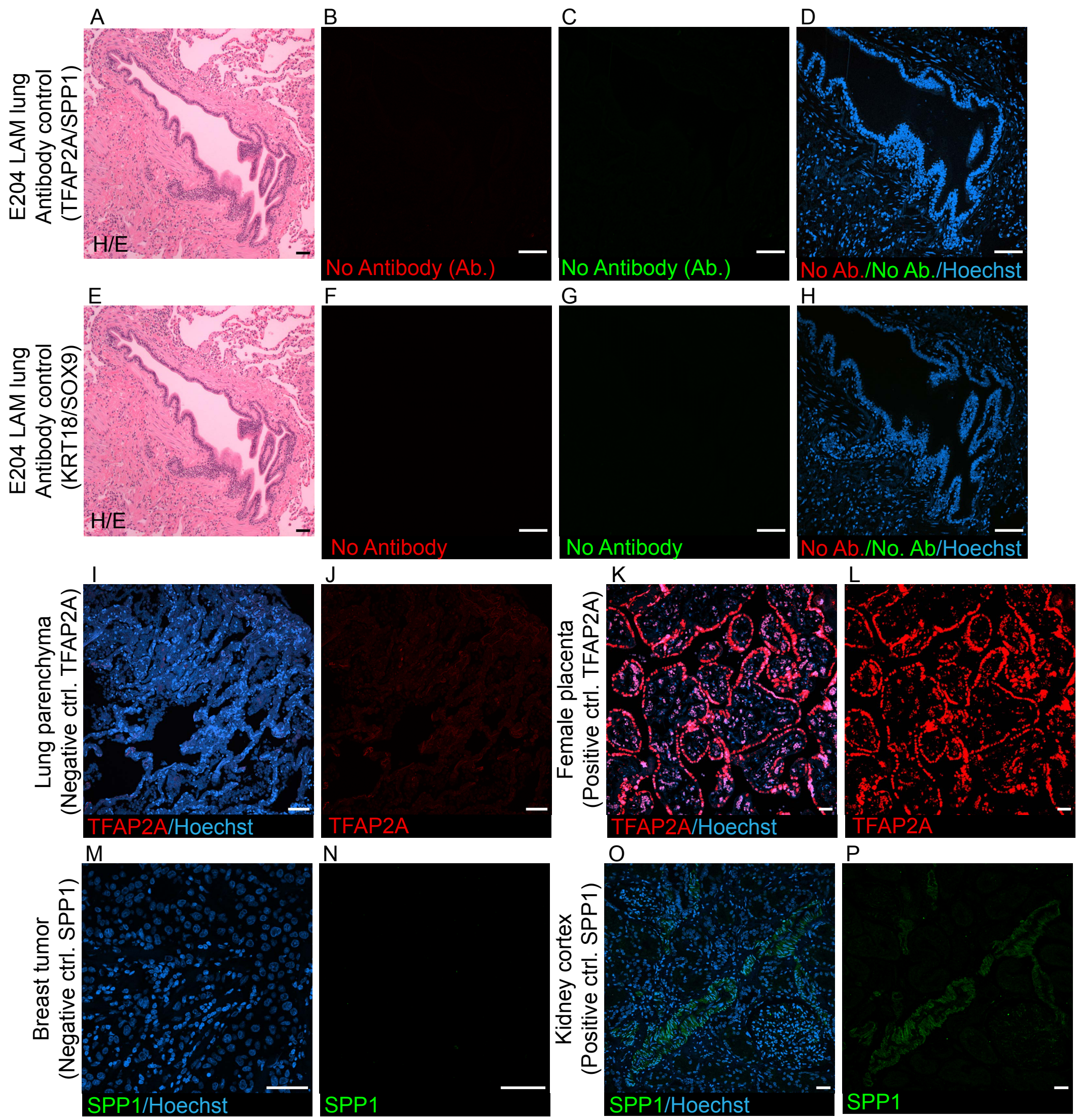

Supplemental Figure 14: Immunohistochemistry of control specimens used to validate neurocristopathic marker detection – FFPE tissue sections (4µm) known to either express (positive control), not express (negative control), or lack primary antibodies (antibody control) were processed as comparative controls in this study. **A-H)** Sections of patient E204 pulmonary neoplasms without primary antibody incubation served as antibody controls in double immunolabeling experiments targeting TFAP2A and SPP1 **(A-D)** and KRT18 and SOX9 **(E-H)**. Lung parenchyma served as a negative control for TFAP2A immunostaining **(I, J)** while its overexpression in female placental trophoblasts acted as positive control **(K, L)**. Mammary tumor tissue **(M, N)** and renal cortex **(O, P)** served as negative and positive control tissue sections respectively in SPP1 IHC studies. All control slides were derived from human tissue and de-identified. Hoechst 33342 was used as a nuclear counterstain. Scale bar: 50µm. Images were captured using a Nikon TiE Eclipse confocal microscope.

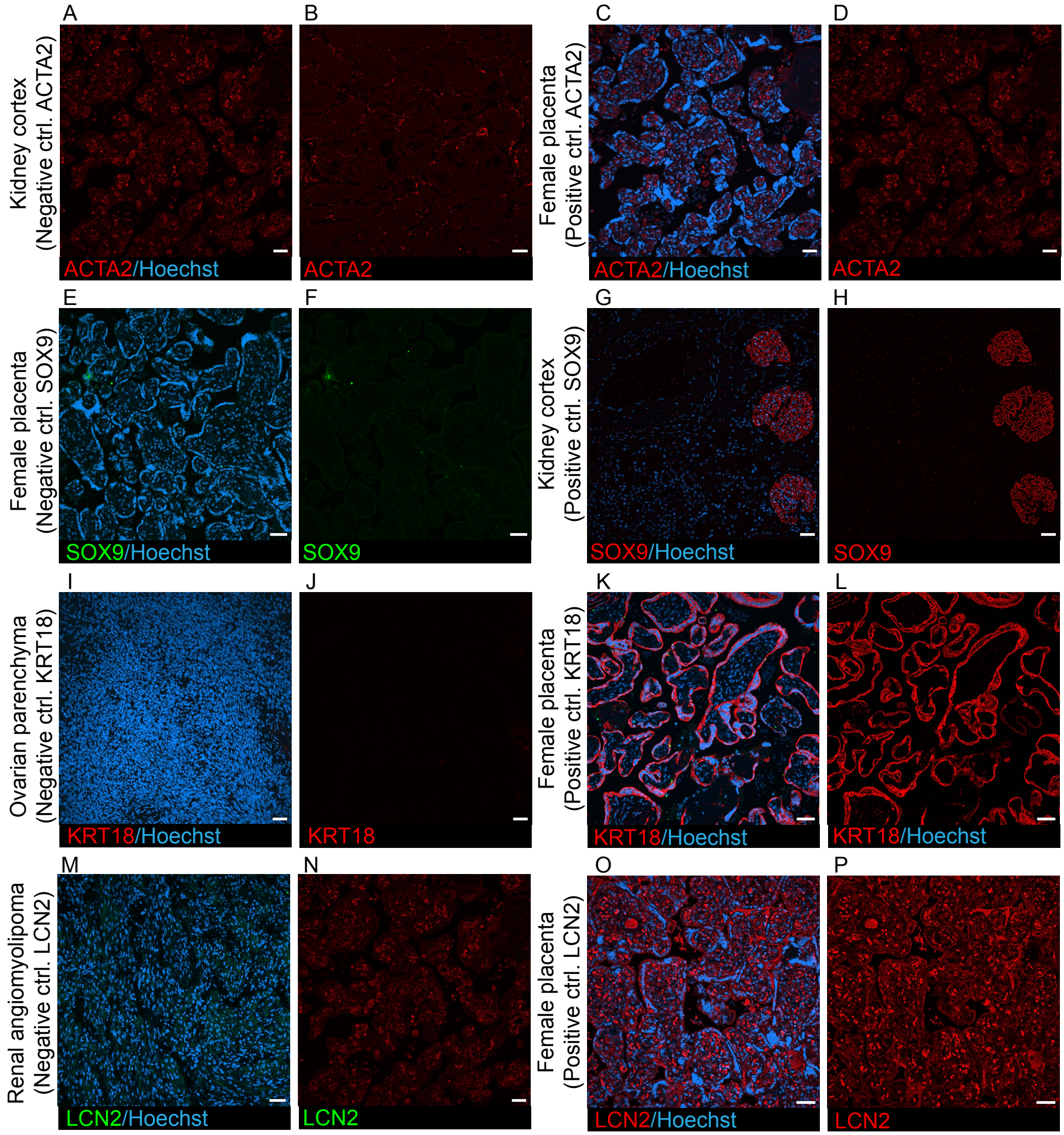

Supplemental Figure 15: Immunohistochemistry of control specimens used to validate neurocristopathic marker detection (contd.) – Renal cortex **(A, B)** and female placenta **(C, D)** FFPE sections (4µm) served as negative control (no marker expression) and positive control (known marker expression) respectively, in smooth muscle actin (ACTA2) IHC. Conversely, placental tissues **(E, F)** and renal glomeruli **(G, H)** were negative and positive contros for SOX9 IHC, respectively. For KRT18 IHC, ovarian parenchyma was negative control **(I, J)**, while placental syncytiotrophoblasts exhibited expected KRT18 positivity **(K, L)**, consistent with their epithelial phenotype. Diseased LAM renal angiomyolipoma (AML) tissues served as negative control for lipocalin-2 (LCN2) **(M, N)**, in concord with decreased LCN2 expression in LAM patient serum **(Figure 10C)** and its high variability directing tumorigenesis in the *Tsc2*$^{+/-}$ mouse model **(Figure 1)**. Conversely, placental tissue sections were used as positive controls **(O, P)**. All control slides are of human origin and have been de-identified. Hoechst 33342 was employed as a nuclear counterstain. Scale bar: 50µm. Images were captured using a Nikon TiE Eclipse confocal microscope.
