# Supplementary material for: Neural crest cell signatures drive tumorigenesis in tuberous sclerosis complex and lymphangioleiomyomatosis
Source: JCI Insight. 2026 Jun 2;11(14):e195013. doi: 10.1172/jci.insight.195013 (PMC13461175; doi:10.1172/jci.insight.195013)
Supplement: Supplementary file 2 [file jciinsight-11-195013-s255.pdf]

UNEDITED WESTERN BLOTS

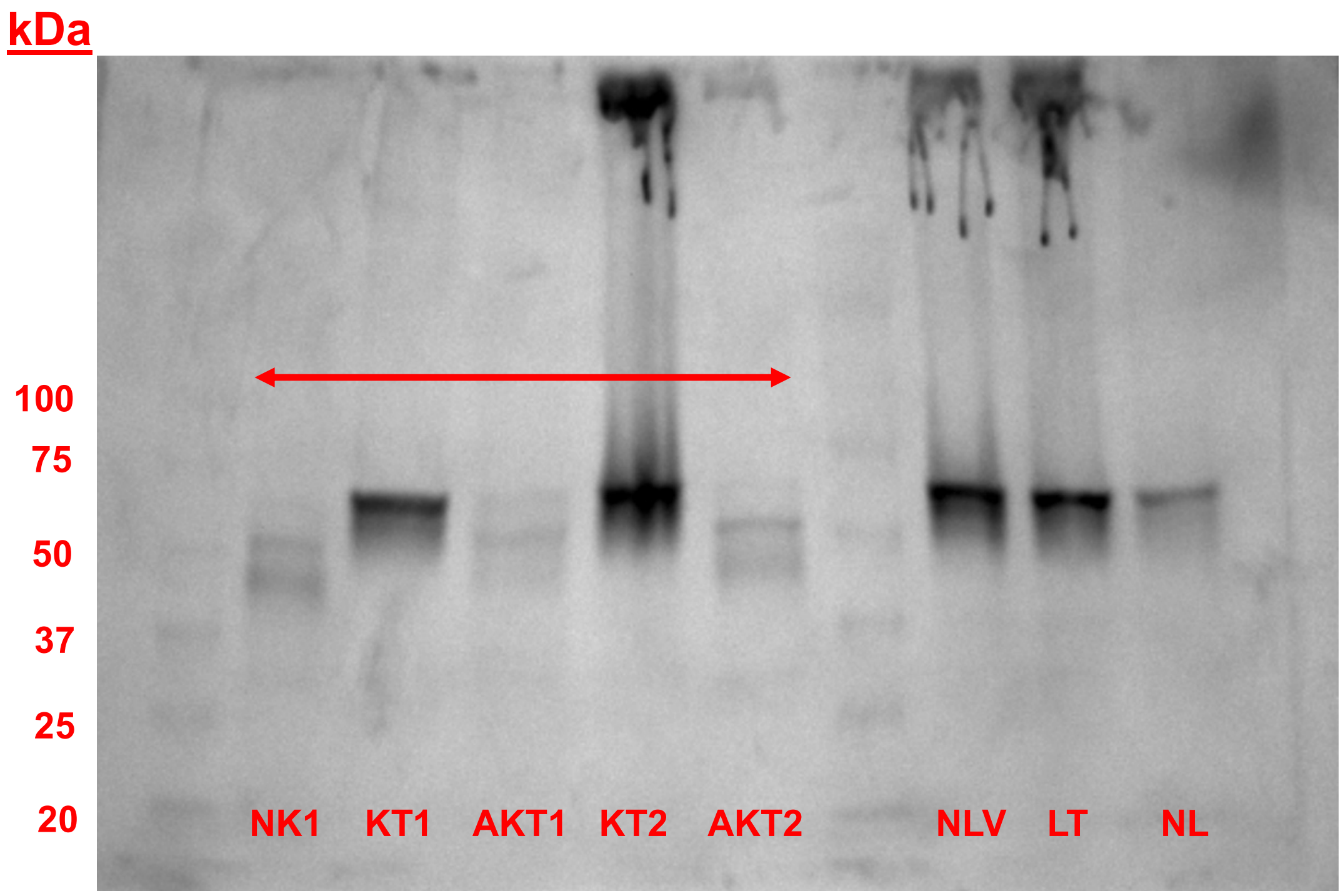

Figure A: Unedited gel of cytokeratin 18 **(Krt18)** expression used in **Figure 3M** with the corresponding ladder. The red arrow spans the bands used in the manuscript. (Mol. Wt. = 48kDa). NK = Normal Kidney; KT = Kidney Tumor; AKT = Adjacent to Kidney Tumor; NLV = Normal Liver; LT = Liver Tumor; NL = Normal Lung

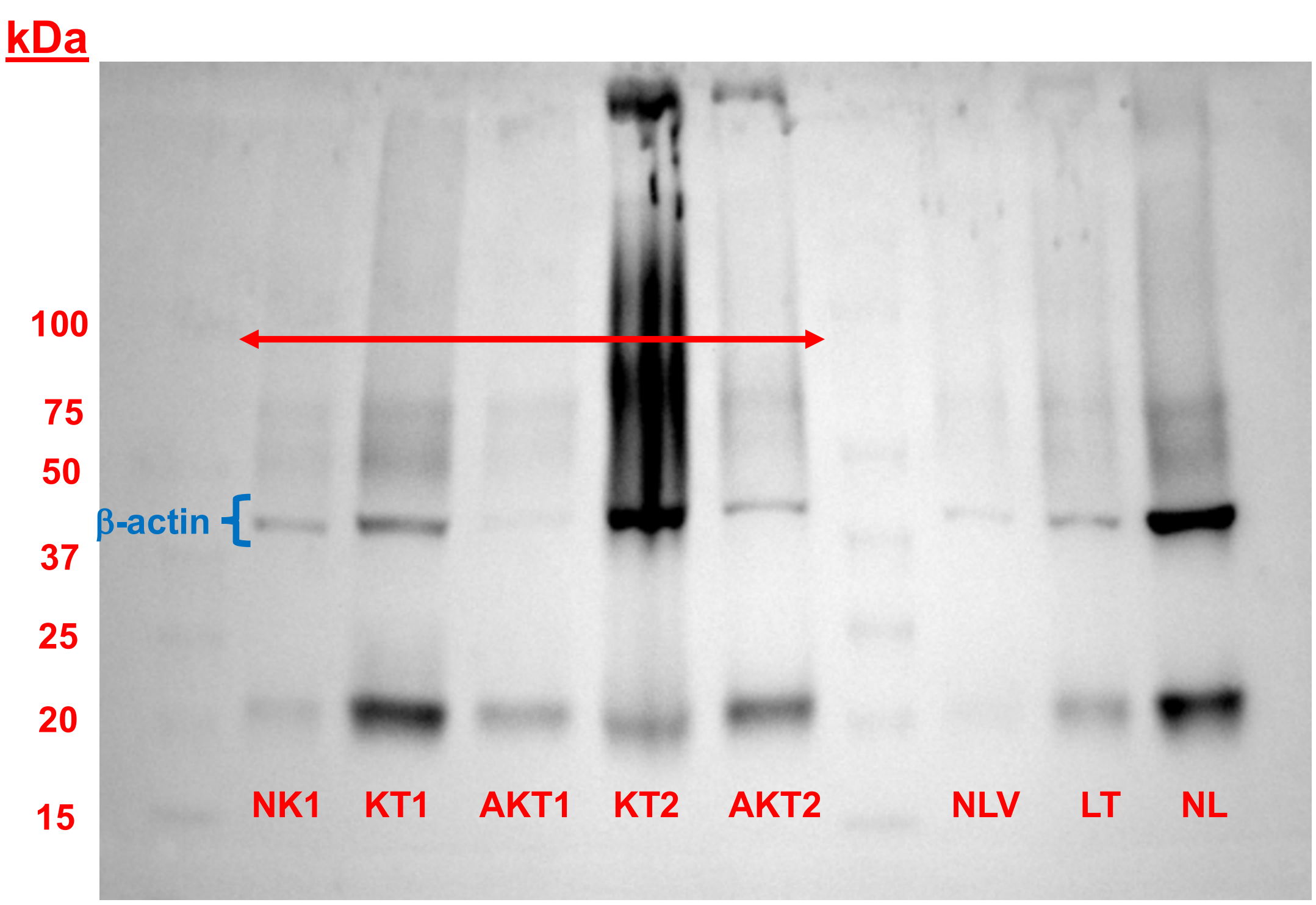

Figure B: Unedited gel of **beta-actin** expression **for Krt18** used in **Figure 3M** with the corresponding ladder. The red arrow spans the bands used in the manuscript. (Mol. Wt. = 42kDa). NK = Normal Kidney; KT = Kidney Tumor; AKT = Adjacent to Kidney Tumor; NLV = Normal Liver; LT = Liver Tumor; NL = Normal Lung

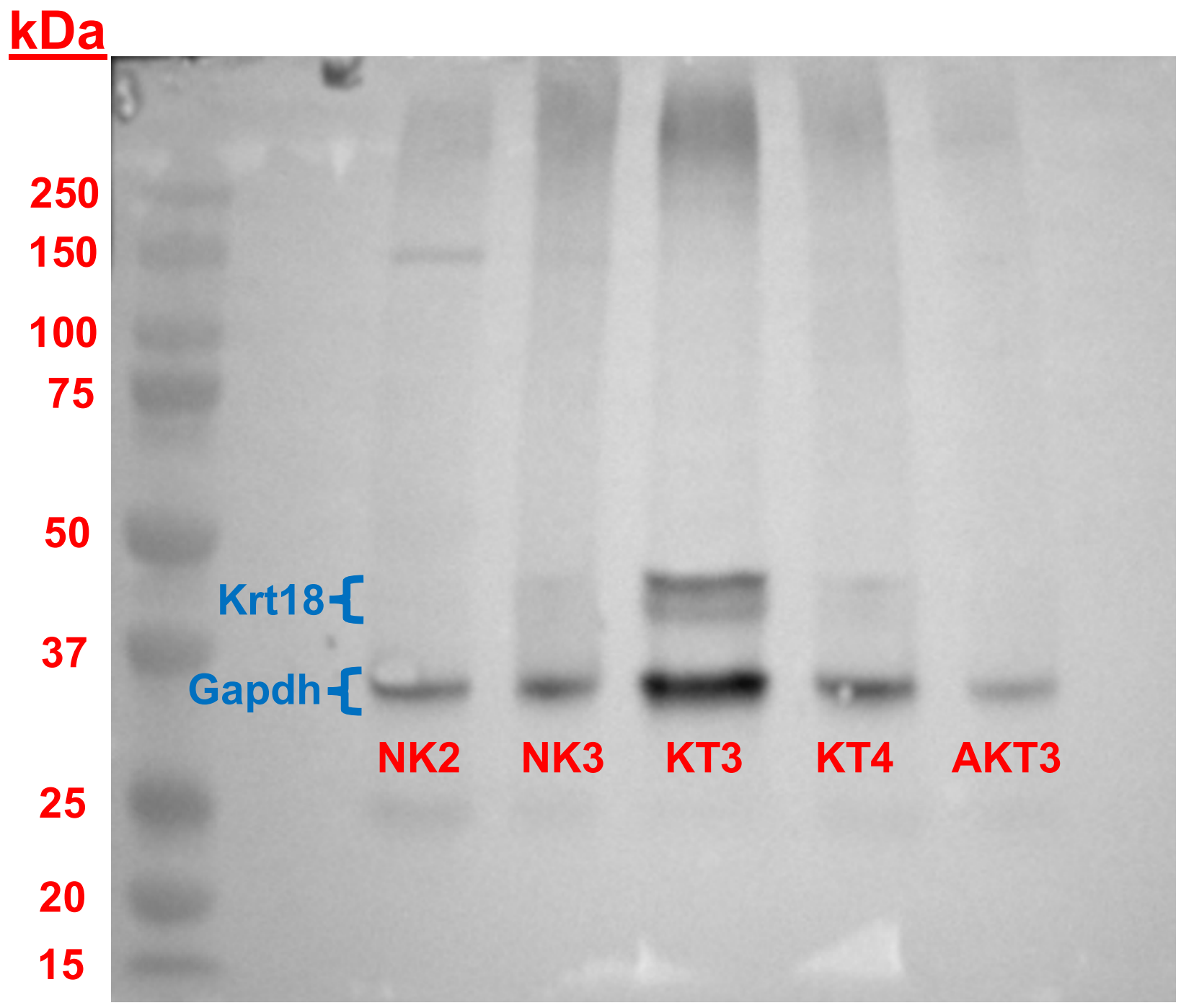

Figure C: Unedited gel of cytokeratin 18 **(Krt18)** (<u>Mol. Wt. = 48kDa</u>) and glyceraldehyde-3-phosphate dehydrogenase **(Gapdh)** (<u>Mol. Wt. = 36kDa</u>) expression for **Figure 3M** with the corresponding ladder. NK = Normal Kidney; KT = Kidney Tumor; AKT = Adjacent to Kidney Tumor.

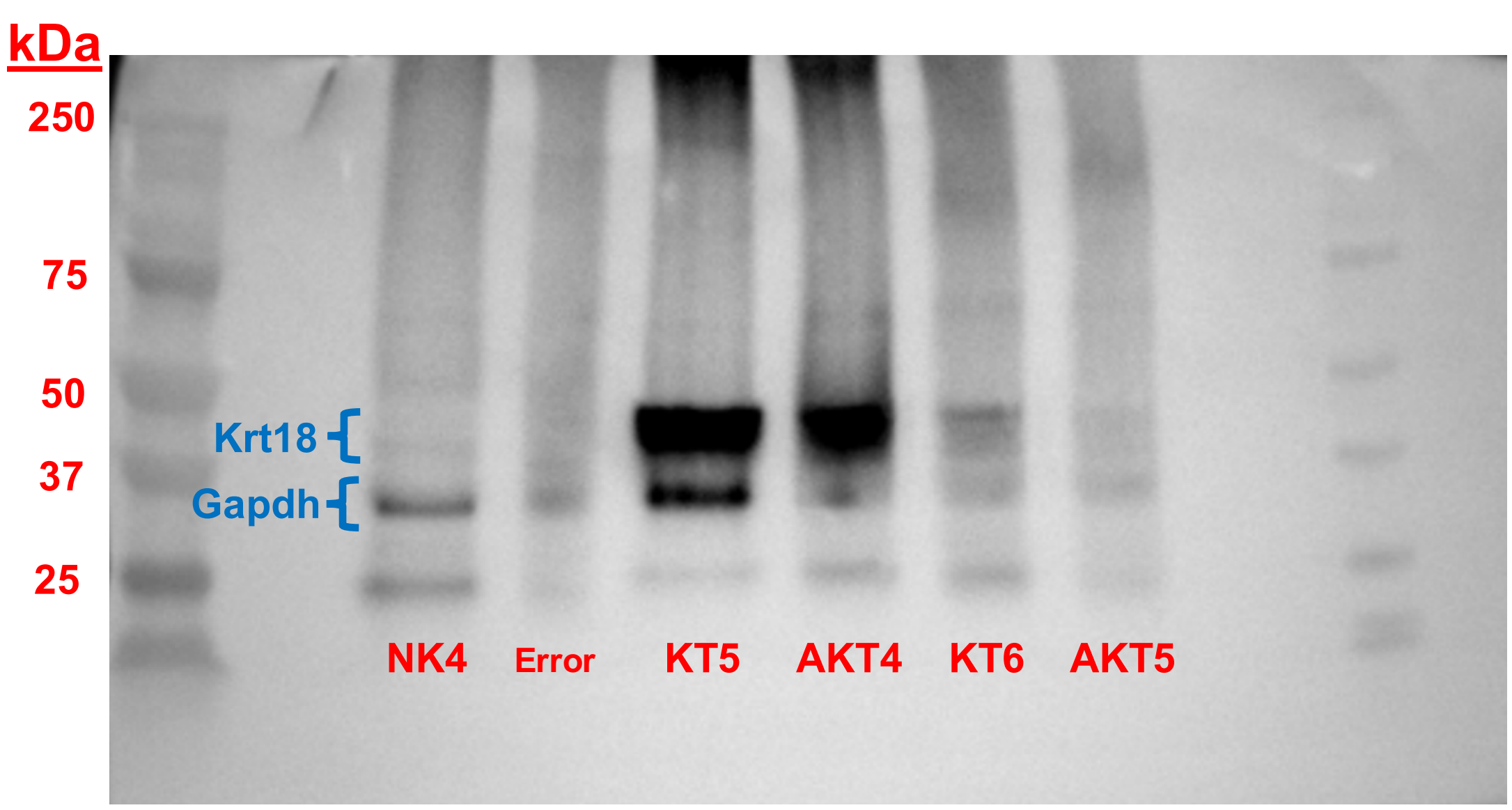

Figure D: Unedited gel of cytokeratin 18 **(Krt18)** (<u>Mol. Wt. = 48kDa</u>) and glyceraldehyde-3-phosphate dehydrogenase **(Gapdh)** (<u>Mol. Wt. = 36kDa</u>) expression for **Figure 3M** with the corresponding ladder. NK = Normal Kidney; KT = Kidney Tumor; AKT = Adjacent to Kidney Tumor.

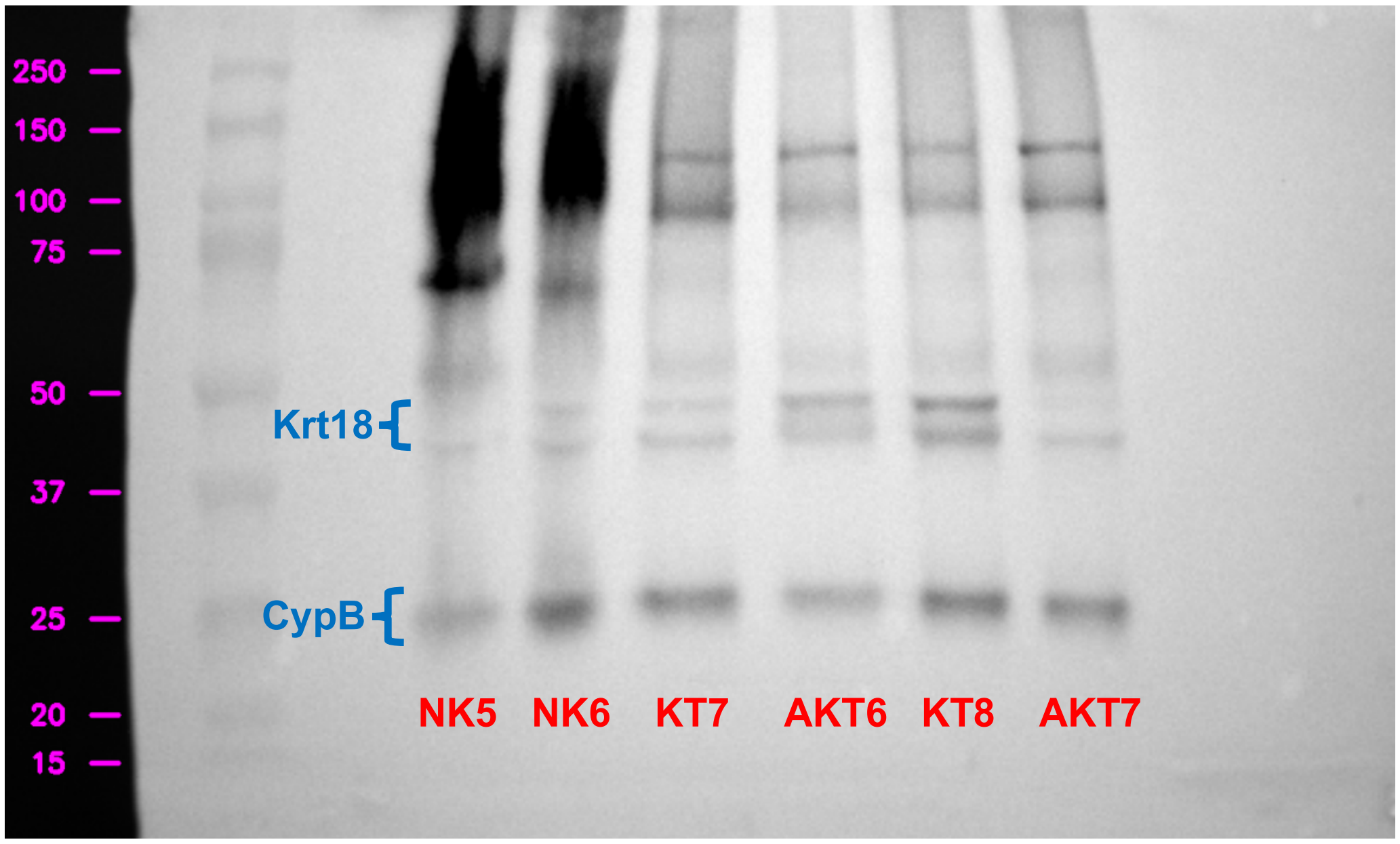

Figure E: Unedited gel of cytokeratin 18 **(Krt18)** (Mol. Wt. = 48kDa) and cyclophilin-B **(Cyp-B)** (Mol. Wt. = 24kDa) expression for **Figure 3M** with the corresponding ladder. NK = Normal Kidney; KT = Kidney Tumor; AKT = Adjacent to Kidney Tumor.

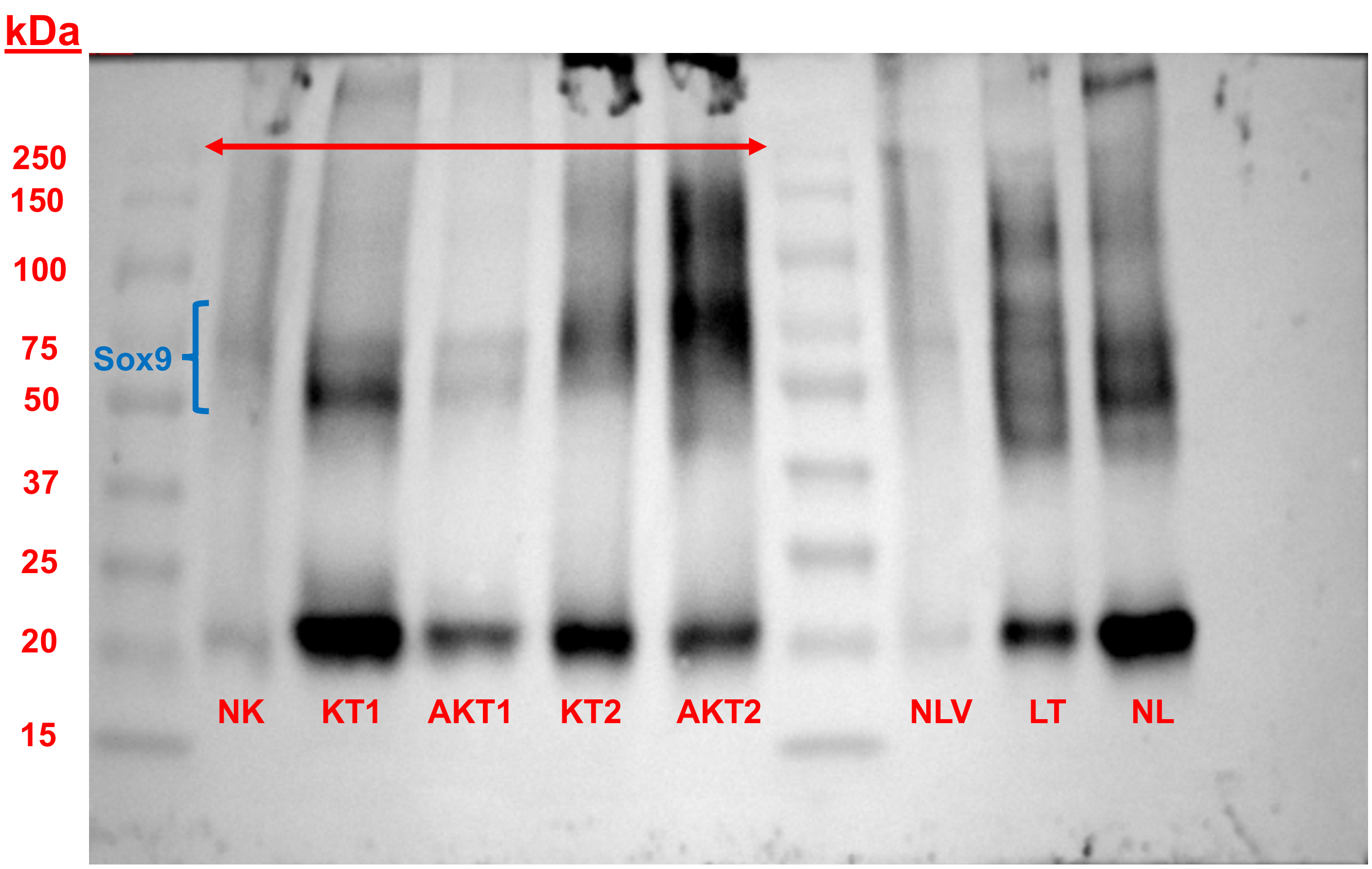

Figure F: Unedited gel of **SRY-Box Transcription Factor 9 (Sox9)** expression for **Figure 3N** with the corresponding ladder. The red arrow spans the bands used in the manuscript. (Mol. Wt. = 56kDa). NK = Normal Kidney; KT = Kidney Tumor; AKT = Adjacent to Kidney Tumor; NLV = Normal Liver; LT = Liver Tumor; NL = Normal Lung

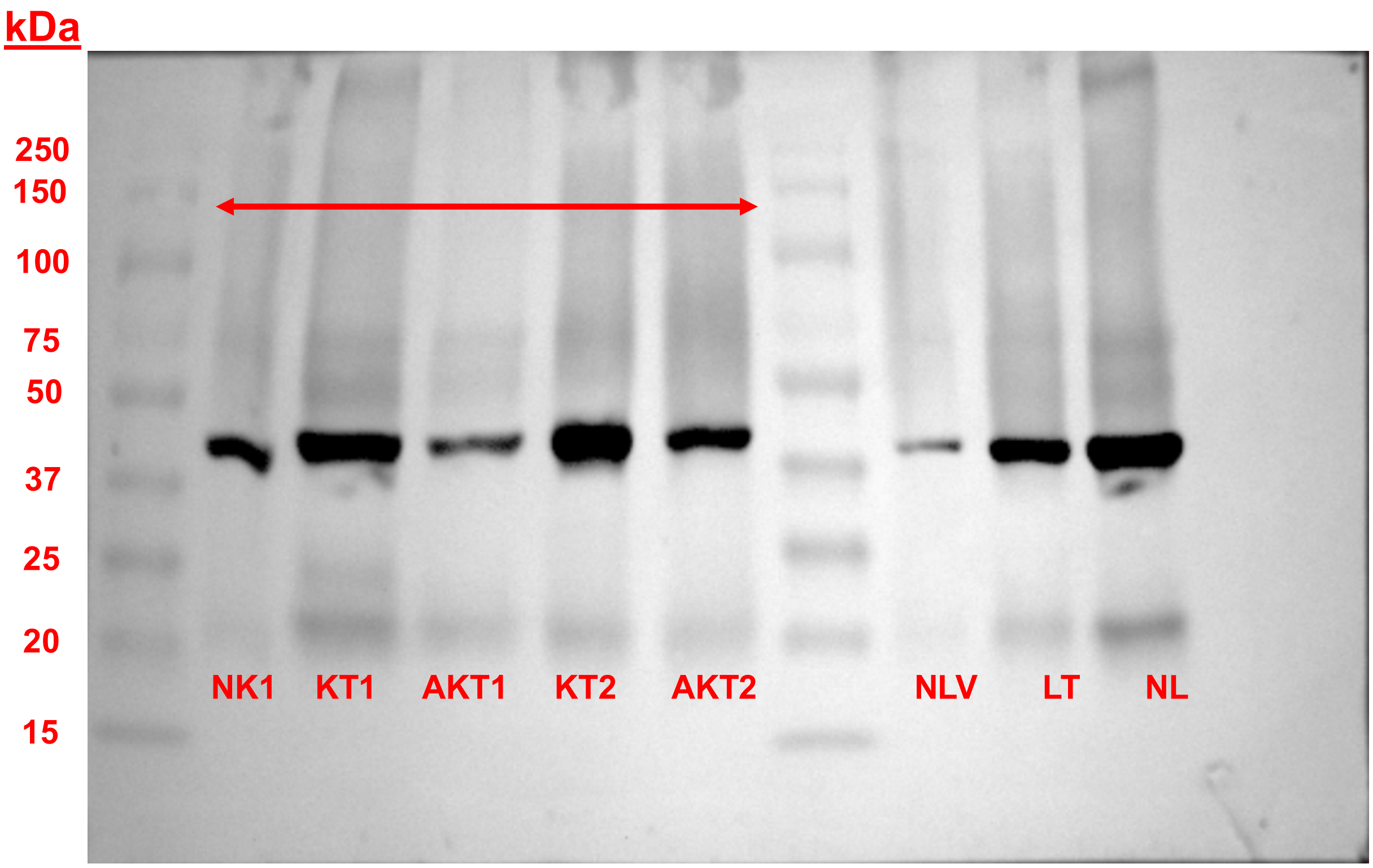

Figure G: Unedited gel of **beta-actin** expression **for SRY-Box Transcription Factor 9 (Sox9)** for **Figure 3N** with the corresponding ladder. The red arrow spans the bands used in the manuscript. Mol. Wt. = 42kDa. NK = Normal Kidney; KT = Kidney Tumor; AKT = Adjacent to Kidney Tumor; NLV = Normal Liver; LT = Liver Tumor; NL = Normal Lung

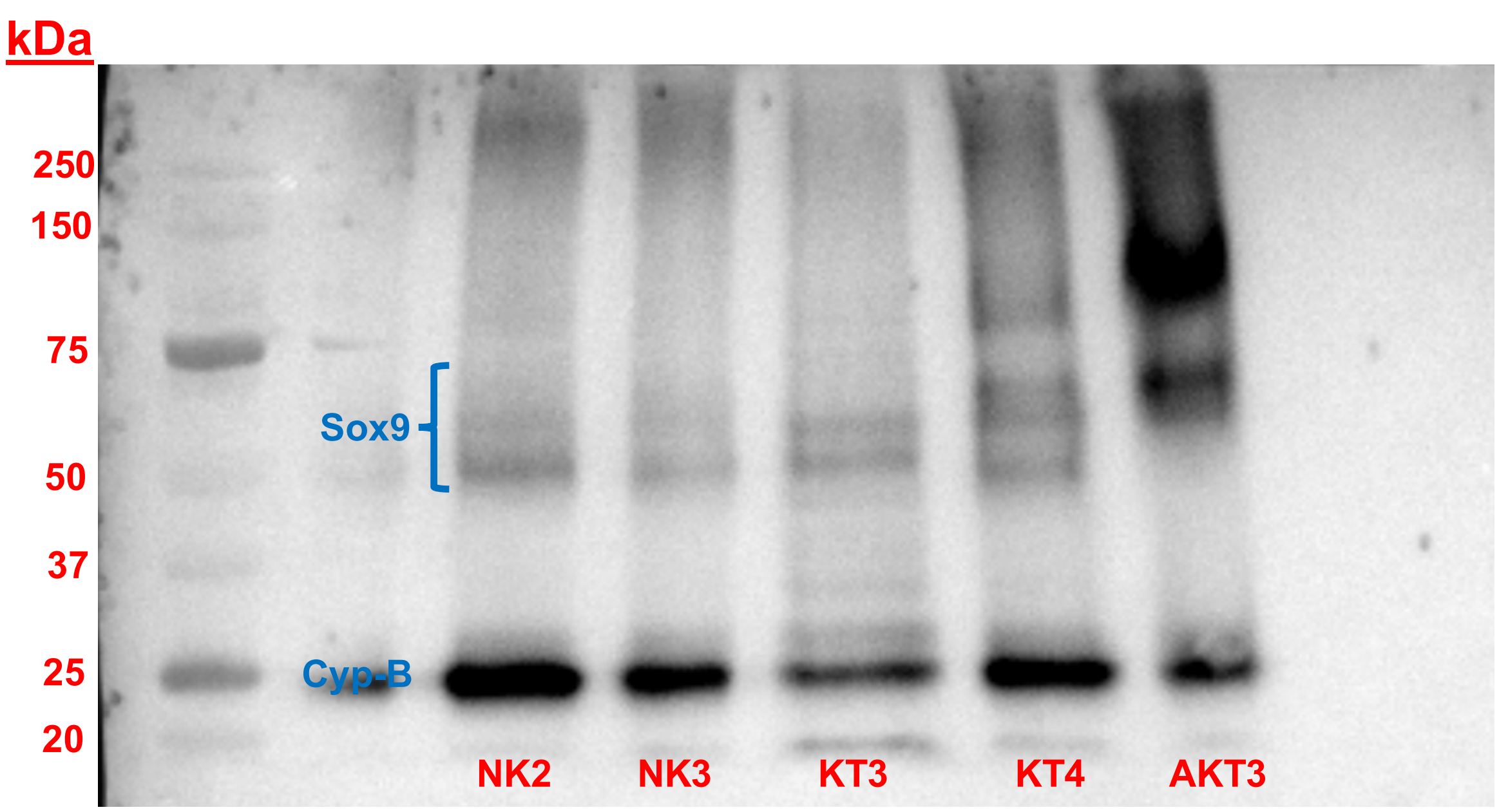

Figure H Unedited gel of **SRY-Box Transcription Factor 9 (Sox9)** (Mol. Wt. = 56kDa) and cyclophilin-B **(Cyp-B)** (Mol. Wt. = 24kDa) expression for **Figure 3N** with the corresponding ladder. NK = Normal Kidney; KT = Kidney Tumor; AKT = Adjacent to Kidney Tumor.

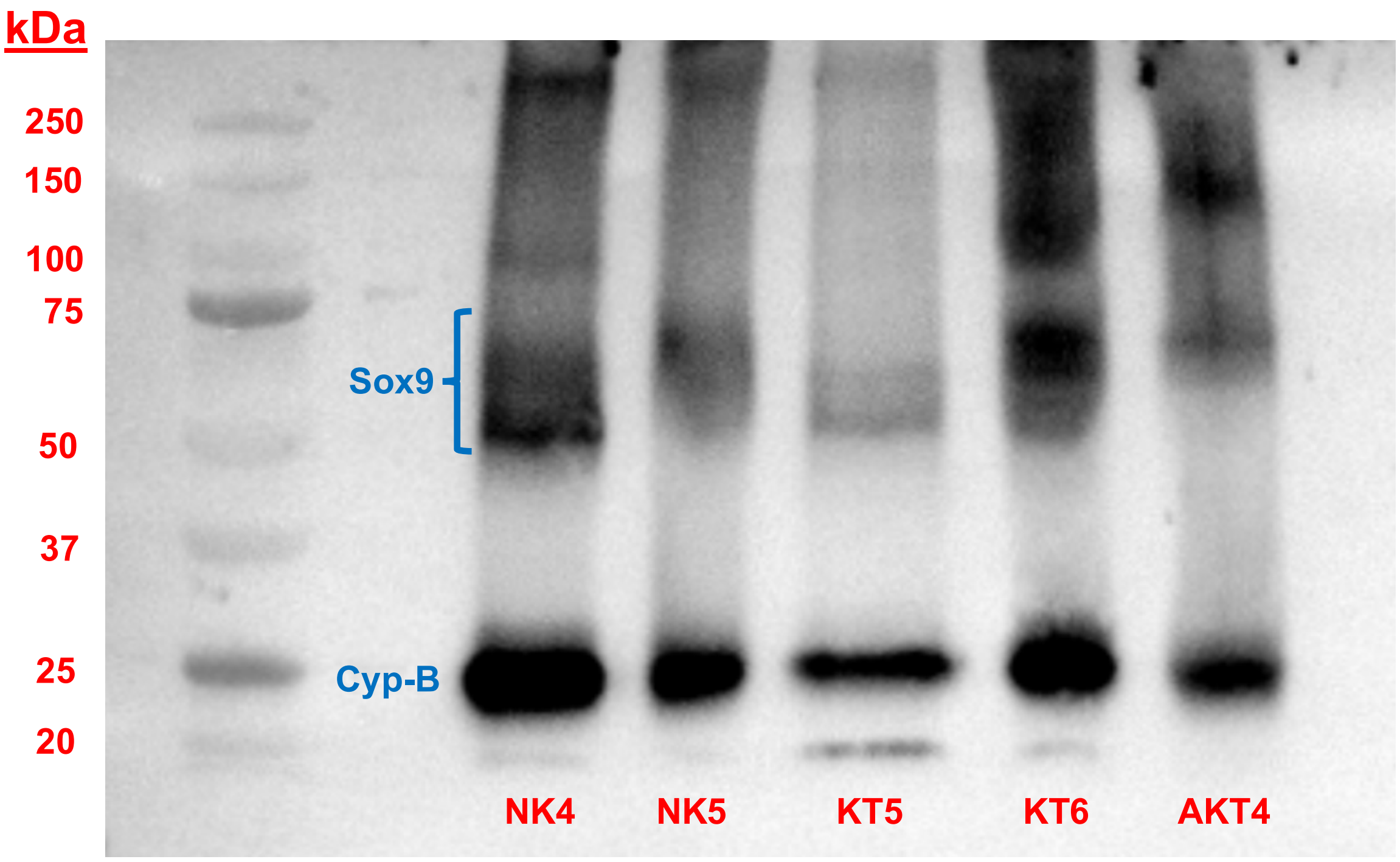

Figure I: Unedited gel of **SRY-Box Transcription Factor 9 (Sox9)** (Mol. Wt. = 56kDa) and cyclophilin-B **(Cyp-B)** (Mol. Wt. = 24kDa) expression for **Figure 3N** with the corresponding ladder. NK = Normal Kidney; KT = Kidney Tumor; AKT = Adjacent to Kidney Tumor.

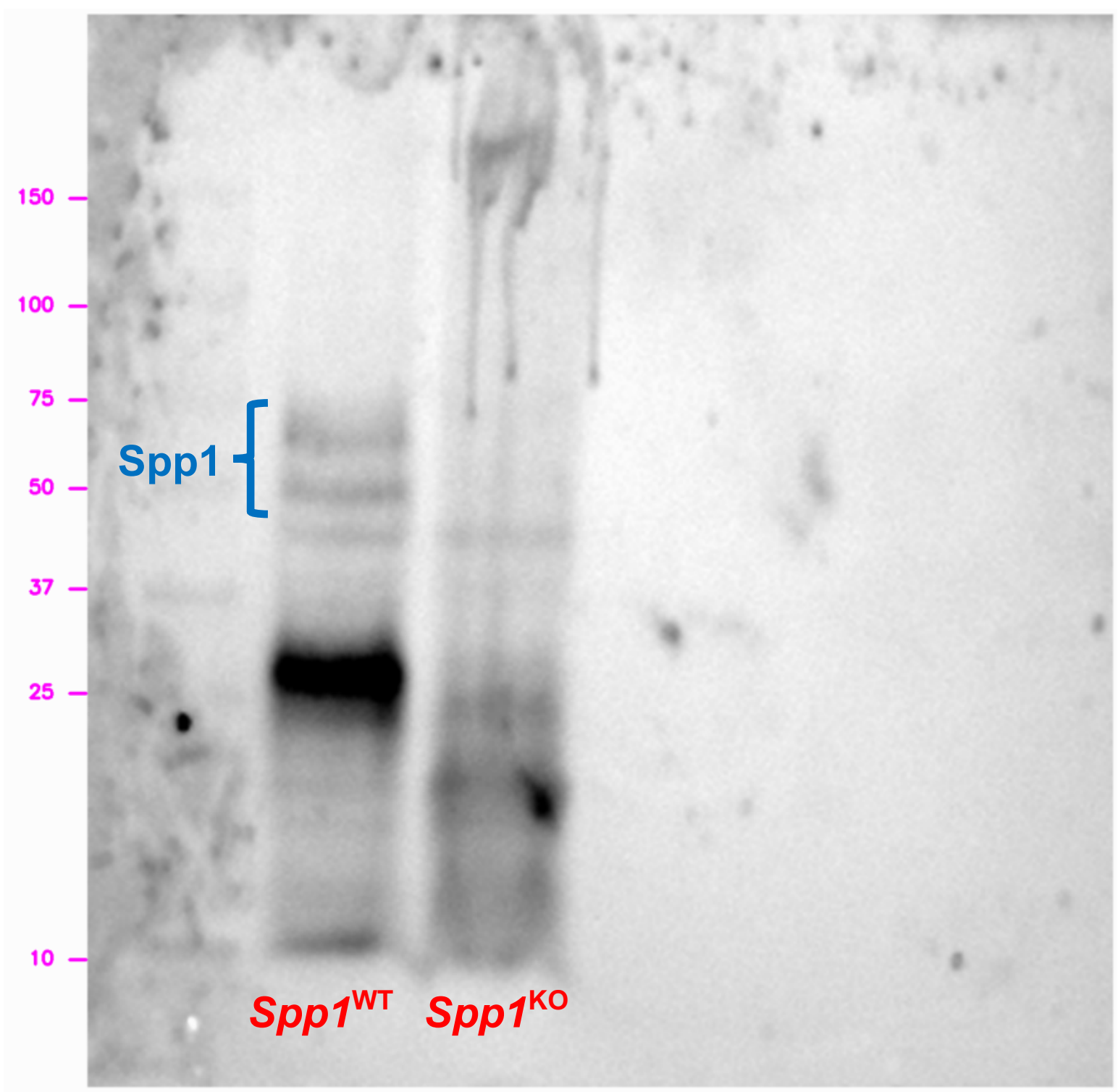

Figure J: Unedited gel of **Osteopontin (Spp1)** (Mol. Wt. = 58-75kDa) expression in lysates of wildtype (WT) and *Spp1*-knockout (KO) mouse cranial neural crest cells (NCCs) for **Figure 11C** with the corresponding ladder.

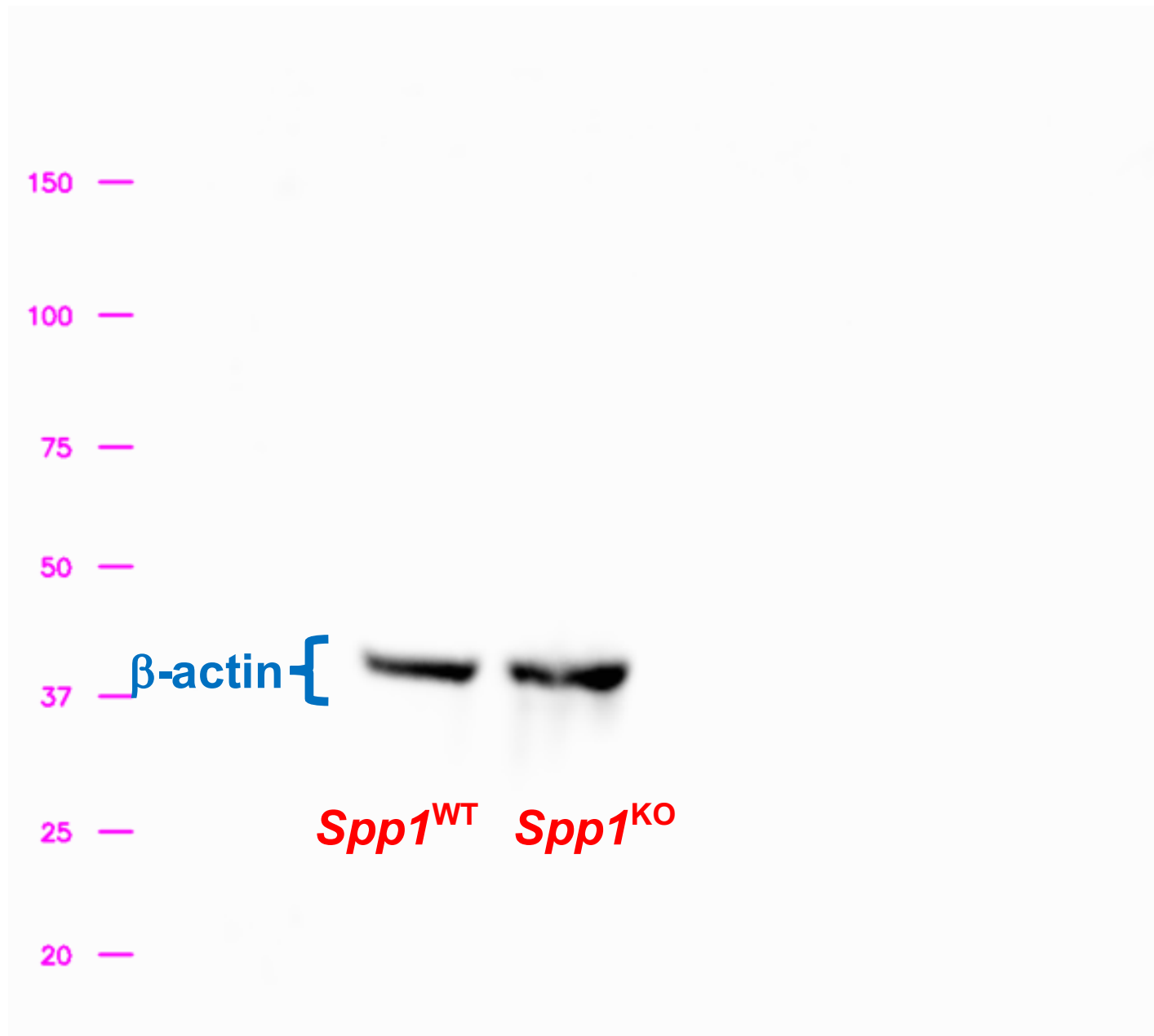

Figure K: Unedited gel of **beta-actin** expression for **Osteopontin (Spp1)** in lysates of wildtype (WT) and *Spp1*-knockout (KO) mouse cranial neural crest cells (NCCs) for **Figure 11C** with the corresponding ladder. Mol. Wt. = 42kDa.

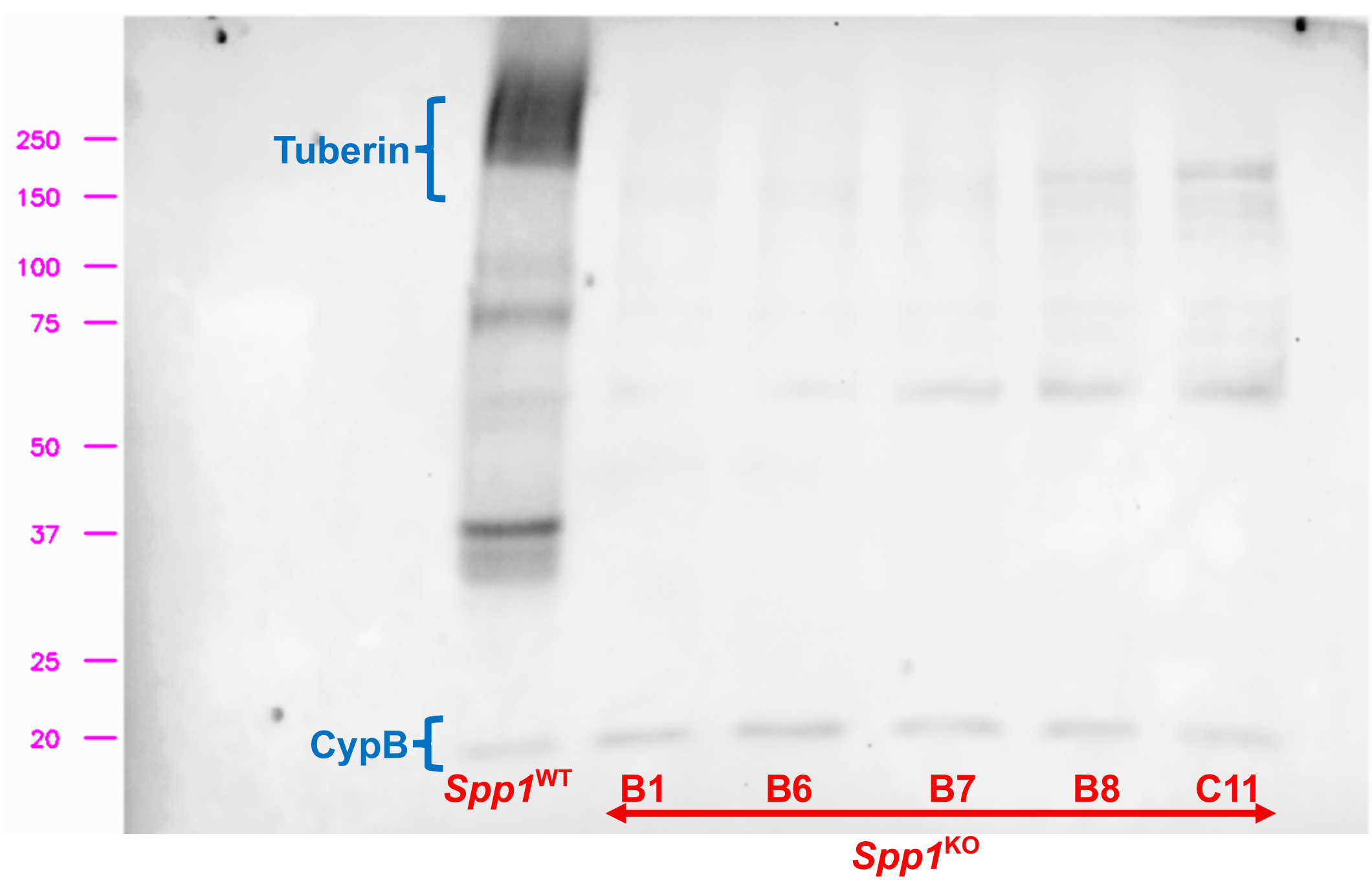

Figure L: Unedited gel of **Tuberin (Tsc2)** (Mol. Wt. = 200kDa) and **cyclophilin-B (Cyp-B)** (Mol. Wt. = 24kDa) expression in lysates of wild-type (WT) and *Tsc2*-knockout (KO) clones B1, of mouse cranial neural crest cell (NCC) for **Figure 11H,** with the corresponding ladder.
